# A Study of the Effects of Hf and Sn on the Microstructure, Hardness and Oxidation of Nb-18Si Silicide-Based Alloys-RM(Nb)ICs with Ti Addition and Comparison with Refractory Complex Concentrated Alloys (RCCAs)

**DOI:** 10.3390/ma15134596

**Published:** 2022-06-30

**Authors:** Eleftherios Zacharis, Claire Utton, Panos Tsakiropoulos

**Affiliations:** Department of Materials Science and Engineering, Sir Robert Hadfield Building, The University of Sheffield, Mappin Street, Sheffield S1 3JD, UK; lefteris.zacharis@alfagro.gr (E.Z.); c.utton@sheffield.ac.uk (C.U.)

**Keywords:** refractory metal intermetallic composites, Nb silicide-based alloys, complex concentrated alloys, alloy design, high-entropy phases, complex concentrated phases, compositionally complex phases

## Abstract

In this paper, we present a systematic study of the as-cast and heat-treated microstructures of three refractory metal intermetallic composites based on Nb (i.e., RM(Nb)ICs), namely the alloys EZ2, EZ5, and EZ6, and one RM(Nb)IC/RCCA (refractory complex concentrated alloy), namely the alloy EZ8. We also examine the hardness and phases of these alloys. The nominal compositions (at.%) of the alloys were Nb-24Ti-18Si-5Hf-5Sn (EZ2), Nb-24Ti-18Si-5Al-5Hf-5Sn (EZ5), Nb-24Ti-18Si-5Cr-5Hf-5Sn (EZ6), and Nb-24Ti-18Si-5Al-5Cr-5Hf-5Sn (EZ8). All four alloys had density less than 7.3 g/cm^3^. The Nb_ss_ was stable in EZ2 and EZ6 and the C14-NbCr_2_ Laves phase in EZ6 and EZ8. In all four alloys, the A15-Nb_3_X (X = Al,Si,Sn) and the tetragonal and hexagonal Nb_5_Si_3_ were stable. Eutectics of Nb_ss_ + Nb_5_Si_3_ and Nb_ss_ + C14-NbCr_2_ formed in the cast alloys without and with Cr addition, respectively. In all four alloys, Nb_3_Si was not formed. In the heat-treated alloys EZ5 and EZ8, A15-Nb_3_X precipitated in the Nb_5_Si_3_ grains. The chemical compositions of Nb_ss_ + C14-NbCr_2_ eutectics and some Nb_5_Si_3_ silicides and lamellar microstructures corresponded to high-entropy or complex concentrated phases (compositionally complex phases). Microstructures and properties were considered from the perspective of the alloy design methodology NICE. The vol.% Nb_ss_ increased with increasing Δχ_Nbss_. The hardness of the alloys respectively increased and decreased with increasing vol.% of A15-Nb_3_X and Nb_ss_. The hardness of the A15-Nb_3_X increased with its parameter Δχ, and the hardness of the Nb_ss_ increased with its parameters δ and Δχ. The room-temperature-specific strength of the alloys was in the range 271.7 to 416.5 MPa cm^3^g^−1^. The effect of the synergy of Hf and Sn, or Hf and B, or Hf and Ge on the macrosegregation of solutes, microstructures, and properties of RM(Nb)ICs/RCCAs from this study and others is compared. Phase transformations involving compositionally complex phases are discussed.

## 1. Introduction

Metallic ultra-high-temperature materials (UHTMs) that are currently under development include refractory metal (RM) intermetallic composites (RMICs) based on Nb (i.e., RM(Nb)ICs), refractory metal high-entropy alloys (RHEAs), and refractory metal complex concentrated alloys (RCCAs) [1,2]. The RM(Nb)ICs are also known as Nb-silicide-based alloys, Nb in situ composites, or Nb in situ silicide composites [1,3], and some of them are also RHEAs or RCCAs, which below are referred to as RM(Nb)ICs/RHEAs or RM(Nb)ICs/RCCAs.

The RHEAs and RCCAs are a subset of high-entropy alloys (HEAs) [2,4,5], the development of which was motivated by [6,7], whereas the development of RM(Nb)ICs was stimulated by research on high-temperature intermetallics [1,8,9]. Currently, metallic UHTMs are being developed for structural engineering applications as potential replacements of Ni-based superalloys in advanced gas turbine engines and must meet specific property goals for fracture toughness, creep, and oxidation [1,3,7]. HEAs target a wider range of potential structural and functional applications [5,10].

A characteristic feature of metallic UHTMs is the fact that they share the same elements [3], which can be simple metal and metalloid elements, rare-earth (RE) elements, transition metals (TMs), and RMs. At least 23 elements and about 16 elements have been used to date, respectively, in RM(Nb)ICs [1,3] and in RHEAs or RCCAs [2], albeit not all in the same metallic UHTM. Chromium, Hf, Mo, Nb, Re, Ta, V, W, and Zr are used in RHEAS and RCCAs, which can also contain Al, Co, Ni, Si, Ti, C, or N [2]. The latter seven elements may form intermetallic phases that may increase the strength and hardness, may improve oxidation, and can decrease the density of RHEAs and RCCAs [2]. The addition of B, Fe, or Y in RCCAs has also been suggested [2].

Currently, Sn is used only in RM(Nb)ICs and RM(Nb)ICs/RCCAs or RM(Nb)ICs/RHEAs [1,3], in which (a) Al, Cr, Hf, Si, Sn, and Ti can significantly improve the oxidation resistance in the range of pest oxidation temperatures and at high temperatures [1,3,11]; (b) Hf, Nb, Si, and Ti are crucial for low- and high-temperature strength and creep properties (note that there is currently only data for the creep of RM(Nb)ICs and RM(Nb)ICs/RCCAs [1,3]); (c) Al, Cr, Hf, Si, and Ti are key for fracture toughness, whereas (d) Al, Hf, Si, Sn, and Ti are key for control of the vol.% of bcc solid solution(s); (e) Al, Cr, Si, Sn, and Ti are key for the vol.% of A15-Nb_3_X compounds or C14-NbCr_2_ Laves phase; and (f) Hf and Ti are key for the type of solid solution that can form [1,3]. In other words, Al, Cr, Hf, Si, Sn, and Ti are key to meeting all three property goals or a balance of properties (together with other alloying additions) [1,3,11].

RM(Nb)ICs and RM(Nb)ICs/RCCAs or RM(Nb)ICs/RHEAs are multiphase alloys, and their microstructures usually consist of bcc solid solution(s) and silicides (M_3_Si, M_5_Si_3_, where M = TM and/or RM) [12]; C14 Laves phase and A15 compounds can also be present [13], in addition to other intermetallics [14,15]. Aluminium, Cr, Hf, Si, Sn, and Ti are found in the chemical composition of all aforementioned phases. Microstructure and properties of RM(Nb)ICs containing these elements have been studied by different groups [16,17,18,19,20,21,22,23,24,25,26,27,28,29,30,31,32,33]. With the exception of one systematic study on the effect of the concentration of Sn (2 to 8 at.%) on the microstructure of as-cast and heat-treated (1200 °C/120 h) MASC-based Nb-25Ti-16Si-8Hf-2Al-2Cr-xSn alloys and their oxidation [16], all other studies and patents have been of Sn-free alloys (e.g., [17,18,19,20]), Hf-free alloys (e.g., [21,22,23]), Al-, Cr-, and Sn-free alloys (e.g., [24,25]), Ti-free alloys [26], or alloys where the elements Al, Cr, Hf, Si, Sn and Ti were in synergy with other TMs or RMs or REs (e.g., [27,28,29,30,31]). (MASC is the RM(Nb)IC Nb-25Ti-16Si-8Hf-2Al-2Cr developed by General Electric [32].)

Aluminium, Cr, Hf, Sn, and Ti partition to the Nb_5_Si_3_ and the bcc Nb_ss_, where their concentrations increase with increasing Ti content (e.g., Figure 20 in [3] and Figure 1 in [34] for Nb_5_Si_3_, and Figure 19 in [3] for the Nb_ss_). Aluminium, Hf, Si, and Ti partition to the C14-NbCr_2_ Laves phase, where the concentrations of Al and Si respectively decrease and increase with increasing Cr concentration (e.g., Figure 21 in [3]); Ti and Hf substitute Nb; and Al, Cr, Hf, Si, Sn, and Ti partition to the A15-Nb_3_X (X = Al,Si,Sn). Thus, two factors make the characterization of microstructures difficult and can mislead phase identification: (a) the chemical inhomogeneity of alloy microstructures, which increases when Al, Cr, Si, and Ti are in synergy with Sn in the absence of Hf [22,23], or with Hf in the absence of Sn [20], or when Al, Cr, Hf, Si, and Sn are in synergy in the absence of Ti [26]; and (b) the similar backscattered electron (BSE) contrast between (i) Nb_ss_ and A15-Nb_3_X, and (ii) the A15-Nb_3_X and Hf-rich Nb_5_Si_3_ (e.g., see [26]). Furthermore, the chemical inhomogeneity can be important vis-à-vis the contamination of RM(Nb)ICs with interstitials [20,22,23]. The contamination of the aforementioned phases with interstitials differs (for example, it is more severe for Nb_ss_ than Nb_5_Si_3_) and depends on their chemical composition (e.g., [20,22,23,31]).

The partitioning of solutes changes the creep properties of the aforementioned phases [11,34]. The steady-state creep rate of RM(Nb)ICs increases with increasing Al or Ti concentration and decreases with increasing Si content in the alloy (Figure 22 in [3]). The synergy of Hf, Nb, and Ti in RM(Nb)ICs is important (i) for their creep properties (for example, the creep deteriorates as the alloy ratio (Nb/Ti+Hf) decreases [12,32,33]); and (ii) for the structure of Nb_5_Si_3_, the likelihood of which being hexagonal increases as the [Nb/(T i+ Hf)]_Nb5Si3_ decreases below one [24].

Even though the importance of the addition of Hf, Sn, or Ti for the properties of RM(Nb)ICs is documented in the literature [32], our understanding of how these three elements “work together” with Al, Cr, and Si is poor, because researchers use all or some of the six elements with other alloying additions in the same alloy. To sort out this problem, our research group did a systematic study of the effect of the synergy of Al, Cr, Hf, Si, Sn, or Ti in the absence of other simple metal and metalloid elements, TMs, RMs, and REs on the microstructure and properties of Nb-24Ti-18Si-based RM(Nb)ICs for alloys without (i) Hf and Sn [21], (ii) Ti [26] and Ti and Sn [25], (iii) Hf [22,23], and (iv) Al, Cr, and Sn [25].

The motivation for the research presented in this paper was to do a systematic study to understand how Hf, Sn, and Ti work in synergy with Al and/or Cr in the microstructure of Nb-24Ti-18Si-based RM(Nb)ICs by expanding our earlier research on Ti-free alloys [26], and to subsequently compare the synergy of Sn or Ge or B with Al, Cr, Hf, Si, and Ti in equivalent RM(Nb)ICs/RCCAs without other alloying addition(s). Four alloys were chosen for this systematic study. Their nominal compositions (at.%) were as follows: Nb-24Ti-18Si-5Hf-5Sn (alloy EZ2), Nb-24Ti-18Si-5Al-5Hf-5Sn (EZ5), Nb-24Ti-18Si-5Cr-5Hf-5Sn (EZ6), and Nb-24Ti-18Si-5Al-5Cr-5Hf-5Sn (EZ8).

The structure of the paper is as follows. After the experimental details, in the results section, the microstructure of each alloy in the as-cast and heat-treated condition is described separately to show how complicated some microstructures can be, and data is given for densities, alloy hardness, and the hardness of phases, as well as the lattice parameter of the solid solution. The discussion covers macrosegregation, then each phase separately, namely Nb_ss_, Nb_5_Si_3_, A15-Nb_3_X, C14-NbCr_2_ Laves, Nb_ss_ + Nb_5_Si_3_ eutectics, Nb_ss_ + C14-NbCr_2_ eutectics, and other lamellar microstructures, then the vol.% of Nb_ss_ and the hardness of alloys and phases, and the specific strength of alloys. Then, we examine properties from the perspective of the alloy design methodology NICE [1,3,11,12]. Finally, the RM(Nb)IC/RCCA alloy EZ8 is compared with the equivalent RM(Nb)ICs/RCCAs alloys ZF9 [35] and TT7 [36], with Ge or B, respectively, in their chemical composition instead of Sn.

## 2. Experimental

The alloys were prepared as large button/ingots of approximately 0.6 kg weight using elements of purity better than 99.99 wt.% and arc melting with a non-consumable tungsten electrode and water-cooled copper crucible in an argon atmosphere. Cubic specimens (2 × 2 × 2 cm^3^) from the bulk of the ingot of each alloy were used for the heat treatments. The cubic specimens were wrapped in Ta foil and placed in a LENTON 1850 high-temperature tube furnace under a constant flow of Ti-gettered argon (10^−5^ m^3^·s^−1^). The microstructures were characterized using X-ray diffraction (XRD) and electron probe microanalysis (EPMA). For the XRD, a Siemens D5000 diffractometer with Cu radiation was used, and X-rays were collected with a step of 0.02 degrees over 2θ range 20 to 90 degrees. Phases were identified using the JCPDS data. The lattice parameter of the Nb_ss_ was determined using the Nelson–Riley function [37]. BSE imaging and quantitative analysis were done in a JEOL 8600 EPMA equipped with energy-dispersive (EDS) and wavelength-dispersive (WDS) spectrometers. Standards of high-purity elements of Nb, Ti, Si, Cr, Al, Hf, and Sn, which had been polished to a finish of 1 µm, were used. The Vickers hardness (HV) of all the alloys in the as-cast and the heat-treated conditions was measured using a CV-430 AAT automatic hardness testing machine with a load of 10 kg (HV10) that was applied for 20 s. At least 10 measurements were taken for each alloy. The hardness of phases in the alloys were measured using a Mitutoyo microhardness testing machine with a load of 0.1 kg that was applied for 20 s. At least 10 measurements were taken for each phase. A Sartorius Masterpro Series electronic analytical balance, along with a Sartorius YDK density determination kit, was used to calculate the density of the alloys. The Archimedean principle was applied for measuring the density of the alloys.

## 3. Results

Data for the densities and hardness of the alloys and the % area of phases in the alloy micro-structures are given in Table 1, which lists the average value, the standard deviation, and the minimum and maximum density and hardness values. The macrosegregation of alloying additions in the cast alloys is given in Table 2. The phases in the microstructures of the as-cast (AC) and heat-treated (HT) alloys are summarized in Table 3. The chemical compositions of different parts of the AC alloys, of the bulk of the HT alloys, and of the phases in all parts of the alloys are given in Tables S1–S4 in the Supplemental Data, which summarize the EPMA data for the whole ingot for the phases that were confirmed using XRD and EPMA. Bottom of the ingot refers to the part of the ingot that was close to the water-cooled Cu crucible during arc melting. These tables give the average value, the standard deviation, and the minimum and maximum analysis values. The XRD data of the alloys for the AC and HT conditions are given in Figures S1–S4 in the Supplemental Data.

### 3.1. As-Cast EZ2

**Figure 1:**
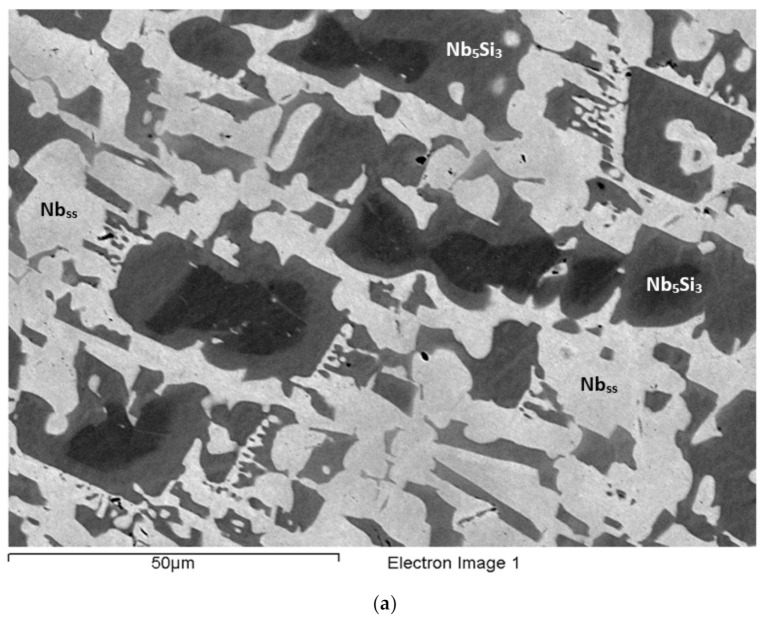

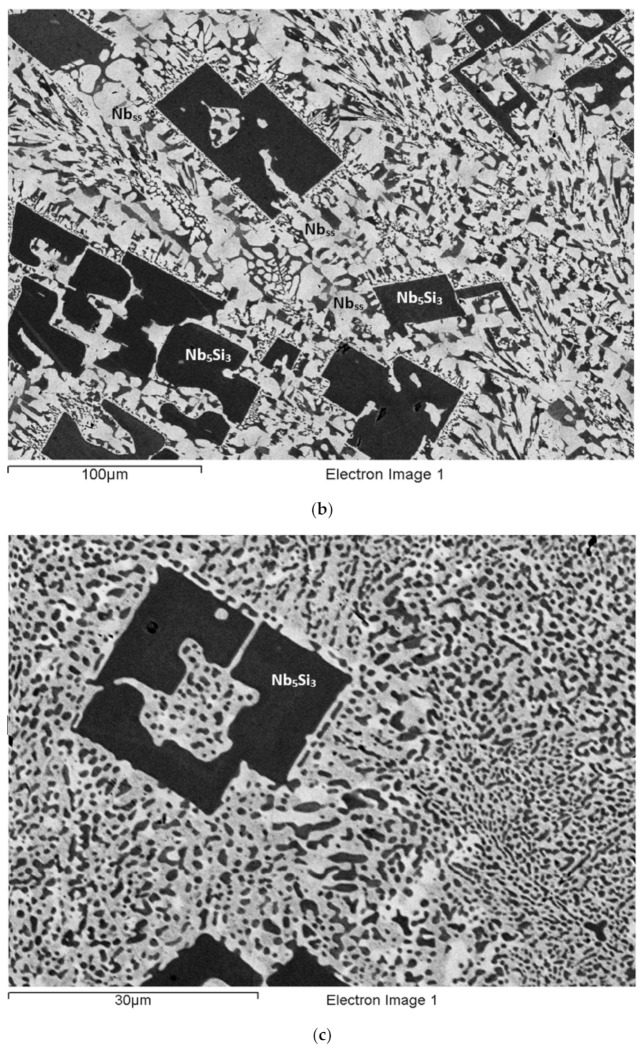

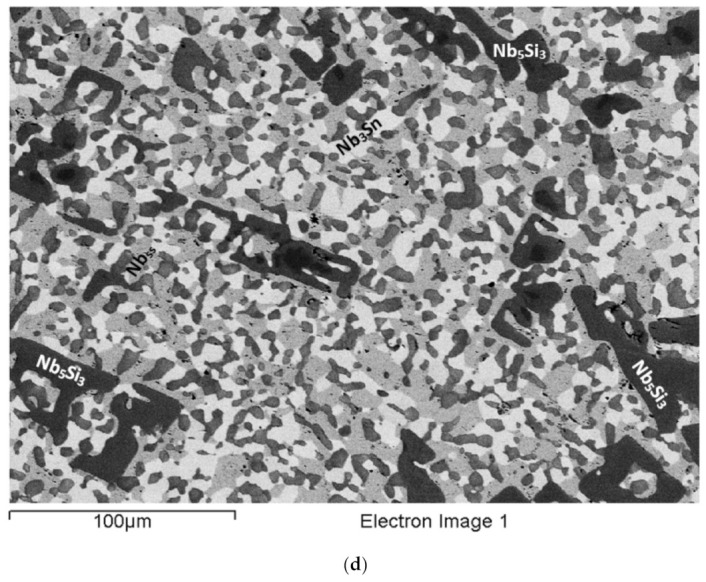
BSE images of EZ2-AC (**a**–**c**) and EZ2-HT (**d**). (**a**) Top, (**b**) bulk, and (**c**) bottom of button/ingot. The A15-Nb_3_X is shown as Nb_3_Sn in (**d**). In (**c**,**d**), the Nb_3_Sn is the phase with brighter contrast than the Nb_ss_ (grey contrast phase). In (**d**), the Hf-rich Nb_5_Si_3_ exhibits contrast slightly darker than the Nb_ss_ and less dark than the large faceted Nb_5_Si_3_.

The actual alloy composition (at.%) was 46Nb-24.1Ti-19.6Si-5.3Hf-5Sn. Compared with the nominal composition, the EZ2-AC was richer in Si. There was macrosegregation of Si and Ti (Table 2). The microstructure consisted of Nb_ss_, Nb_5_Si_3_, and A15-Nb_3_X (X = Si, Sn) phases (Table 3, Figure 1 and Figure S1 in the Supplemental Data). No hafnia particles were observed. There was eutectic of the Nb_ss_ and Nb_5_Si_3_ (Figure 1) and Ti-rich Nb_ss_ and Hf-rich Nb_5_Si_3_ (Table 3 and Table S1 in the Supplemental Data). Note that the latter was also rich in Ti. Eutectic was observed in-between or around faceted Nb_5_Si_3_ grains (Figure 1). The Ti-rich Nb_ss_ exhibited darker contrast under BSE imaging, had Nb/Ti < 1, and was also richer in Hf and Sn compared with the “normal” Nb_ss_ (Table S1 in the Supplemental Data). The Hf-rich Nb_5_Si_3_ exhibited a brighter contrast than the Nb_5_Si_3_. This can be seen in the Figure 1a, where the darker contrast “core” of the silicide grains had average Ti/Hf = 4.3, compared with 2.8 for the surrounding grey contrast Hf-rich (and Ti-rich) Nb_5_Si_3_. The Nb/(Ti + Hf) ratios for the dark and grey contrast areas of the silicide were 1.8 and 1.97, respectively. According to the XRD data (Figure S1 in the Supplemental Data), both αNb_5_Si_3_ and βNb_5_Si_3_ were present. Considering the analyses in all parts of the ingot and the Nb/(Ti + Hf) ratios of the Nb_5_Si_3_ and the Hf-rich Nb_5_Si_3_ (which were 2.3 and 1.6, respectively), the data would suggest that there was no hexagonal Nb_5_Si_3_ in the microstructure, in agreement with the XRD data. The A15-Nb_3_X was confirmed only in the bottom of the ingot using EPMA. It exhibited brighter contrast than the Nb_ss_ (Figure 1c). The microstructure in the bottom of the ingot was similar to that shown in Figure 1b.

**Figure 2:**
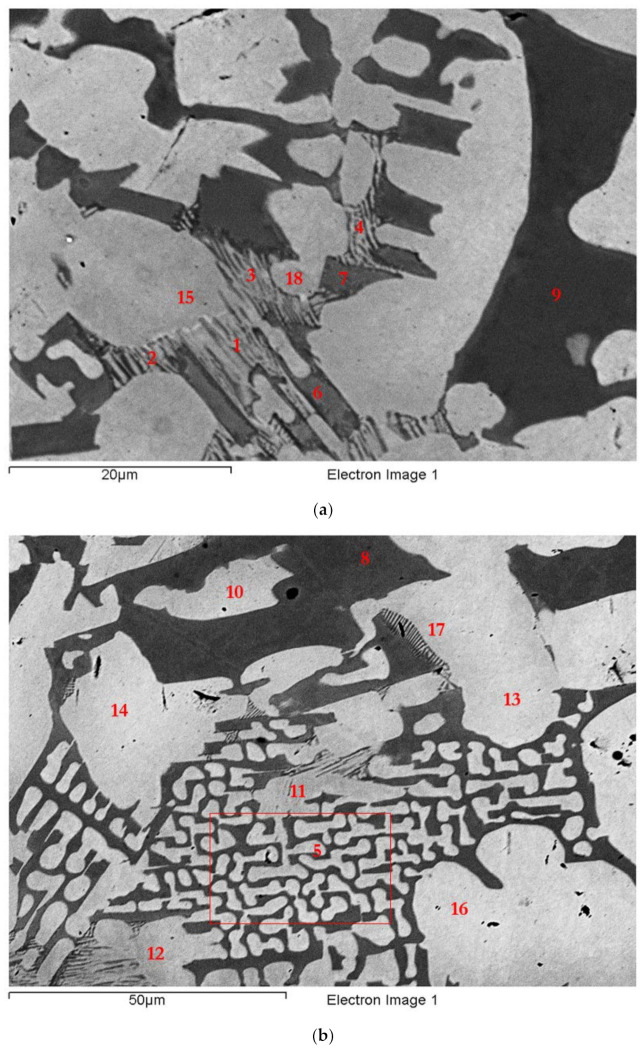
(**a**,**b**) show details of the microstructure in some parts of the bulk of the ingot of EZ2-AC. See text for numbers.

The microstructure in the top and bulk of the ingot was the same and comprised of Nb_ss_ and Nb_5_Si_3_. In some areas of the bulk, the microstructure in the vicinity of blocky faceted Nb_5_Si_3_ was different from that shown in Figure 1. This microstructure, which is shown in Figure 2, was observed near Nb_ss_ + Nb_5_Si_3_ eutectic (see 5 in Figure 2b) and consisted of a lamellar microstructure, which either (i) grew into a blocky faceted Nb_5_Si_3_ grain (see the left-hand side of number 17 in Figure 2b) or (ii) connected adjacent Nb_5_Si_3_ grains (indicated with the numbers 1, 2, 3, and 4 in Figure 2a). The average composition (at.%) of the lamellar microstructure was 28.9Nb-38.9Ti-17.2Si-8.5Hf-6.5Sn, that is, a complex concentrated or compositionally complex (CC) lamellar microstructure [11], with Si + Sn, Si/Sn, Ti/Hf, Ti + Hf and Nb/(Ti + Hf) of about 23.7 at.%, 2.65, 4.6, 47.4 at.%, and 0.6, respectively.

The average composition of the eutectic seen adjacent to the lamellar microstructure (e.g., see 5 in Figure 2b) was 44.1Nb-26.2Ti-18.1Si-5.7Hf-5.9Sn (at.%) and had Si+Sn, Si/Sn, Ti/Hf, Ti+Hf, and Nb/(Ti+Hf) of about 24.1 at.%, 3.1, 4.6, 31.9 at.%, and 1.4, respectively. The average composition of the eutectic surrounding the faceted Nb_5_Si_3_ grains (Figure 1b) was 49.8Nb-22.9Ti-16.9Si-4.8Hf-5.6Sn (at.%) with Si+Sn, Si/Sn, Ti/Hf, Ti+Hf, and Nb/(Ti + Hf) of about 22.5 at.%, 3.1, 4.8, 27.7 at.%, and 1.8, respectively. The average composition of the Nb_ss_+Nb_5_Si_3_ eutectic in the ingot (e.g., see Figure 1c) was 46.8Nb-26.5Ti-15.5Si-5.5Hf-5.7Sn (at.%) with Si+Sn, Si/Sn, Ti/Hf, Ti + Hf, and Nb/(Ti + Hf) of about 21.2 at.%, 2.7, 4.8, 32 at.%, and 1.5, respectively. In other words, the lamellar microstructure shown in Figure 2 had significantly different Ti + Hf sum and Nb/(Ti + Hf) ratio compared with the eutectic in EZ2-AC.

The Nb_5_Si_3_ grains in between the lamellar microstructure (e.g., 6 and 7 in Figure 2a) were rich in Hf and Ti, with average composition 30.5Nb-22.4Ti-36.2Si-8.7Hf-2.2Sn (at.%) and Si+Sn, Si/Sn, Ti/Hf, Ti + Hf, and Nb/(Ti + Hf) of about 38.4 at.%, 16.5, 2.6, 31.1 at.%, and 1, respectively, which corresponds to a complex concentrated or compositionally complex (CC) [11] tetragonal Nb_5_Si_3_ based on the Nb/(Ti + Hf) ratio. The blocky faceted Nb_5_Si_3_ grains near the lamellar microstructure (e.g., 9 in Figure 2a and 8 in Figure 2b) had average composition 43.75Nb-13.5Ti-36.3Si-4.85Hf-1.6Sn (at.%) with Si+Sn, Si/Sn, Ti/Hf, Ti + Hf, and Nb/(Ti + Hf) about 37.9 at.%, 22.7, 2.8, 18.4 at.%, and 2.4, respectively, which (i) is consistent with tetragonal Nb_5_Si_3_ in accordance with its Nb/(Ti + Hf) ratio and (ii) was not significantly different from the average composition of Nb_5_Si_3_ in the whole of the ingot (see Table S1 in Supplemental Data). Hexagonal Nb_5_Si_3_ was not confirmed by XRD. In other words, the Hf (and Ti)-rich Nb_5_Si_3_ that was connected (associated) with the lamellar microstructure (or the Hf (and Ti)-rich Nb_5_Si_3_ from which the lamellar microstructure formed) was CC tetragonal Nb_5_Si_3_ that had higher Ti + Hf sum and lower Nb/(Ti + Hf) ratio than the blocky Nb_5_Si_3_.

The solid solution away from the lamellar microstructure (e.g., 10, 13, 14, and 16 in Figure 2b) had average composition 53Nb-31.9Ti-2.65Si-4.45Hf-8Sn (at.%), not significantly different from the average composition of Nb_ss_ in the whole of the ingot (see Table S1 in the Supplemental Data), and it had Si+Sn, Si/Sn, Ti/Hf, Ti + Hf, and Nb/(Ti + Hf) of about 10.65 at.%, 0.33, 7.2, 36.35 at.%, and 1.46, respectively. The Nb_ss_ close to the lamellar microstructure (e.g., 11, 12, 15, 17, and 18 in Figure 2) had average composition 43.2Nb-39.9Ti-2.4Si-5.7Hf-8.8Sn (at.%), which was different from the average composition of the Ti-rich Nb_ss_ in the whole of the ingot (see Table S1 in the Supplemental Data), and it had Si+Sn, Si/Sn, Ti/Hf, Ti + Hf, and Nb/(Ti + Hf) of about 11.2 at.%, 0.27, 7, 45.6 at.%, and 0.95, respectively. In other words, the Nb_ss_ associated with the lamellar microstructure had higher Ti + Hf sum and lower Nb/(Ti + Hf) ratio compared with the Nb_ss_ away from it.

The microstructure near the bottom of the ingot (Figure 1c) consisted of three phases, namely the Nb_ss_, A15-Nb_3_X, and Nb_5_Si_3_. There was no Ti-rich Nb_ss_, only Hf (and Ti)-rich Nb_5_Si_3_. The Nb_5_Si_3_ and Hf-rich Nb_5_Si_3_ had Nb/(Ti + Hf) of, respectively, 2.4 and 1.5, which would suggest tetragonal Nb_5_Si_3_. The Si+Sn sum and the Si/Sn ratios of the Nb_ss_ and A15-Nb_3_X were 10 at.% and 0.3, and 18.9 at.% and 0.4, respectively.

### 3.2. Heat-Treated EZ2 (1500 °C/100 h)

The XRD (Figure S1 in the Supplemental Data) and EPMA (Table S1 in the Supplemental Data) indicated Nb_ss_, A15-Nb_3_X, and Nb_5_Si_3_ in the microstructure. Hf (and Ti)-rich Nb_5_Si_3_ was also present. The typical microstructure is shown in Figure 1d. The Si+Sn concentration of the Nb_ss_ was 6.6 at.%, compared with 10.3 at.% in EZ2-AC, with the concentrations of Si and Sn about 1.1 at.% and 2.6 at.% lower, but the Si/Sn ratio was the same (0.3). The A15-Nb_3_X had Si + Sn and Si/Sn of, respectively, 18.7 at.% and 0.3, and the Sn concentration had increased by about 1 at.%. The Nb/(Ti + Hf) ratios for the Nb_5_Si_3_ and the Hf-rich Nb_5_Si_3_ were, respectively, 2.1 and 0.7; the latter value would suggest hexagonal Nb_5_Si_3_ (see Figure S1 in the Supplemental Data).

### 3.3. As-Cast EZ5

**Figure 3:**
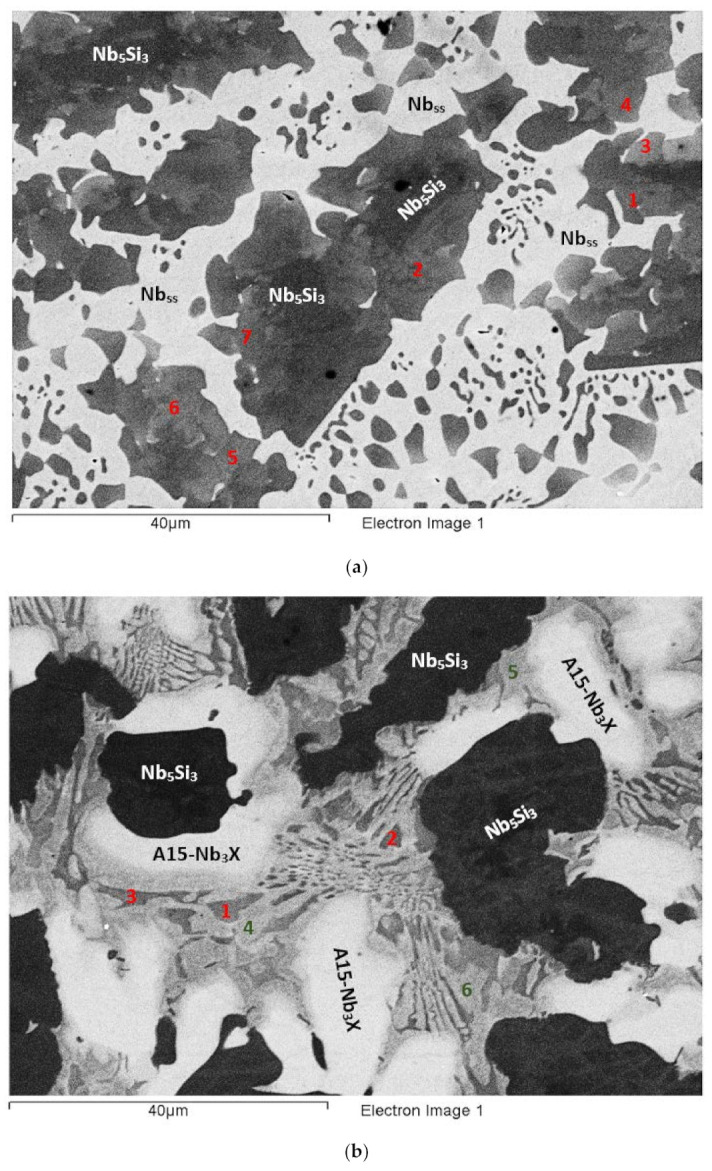

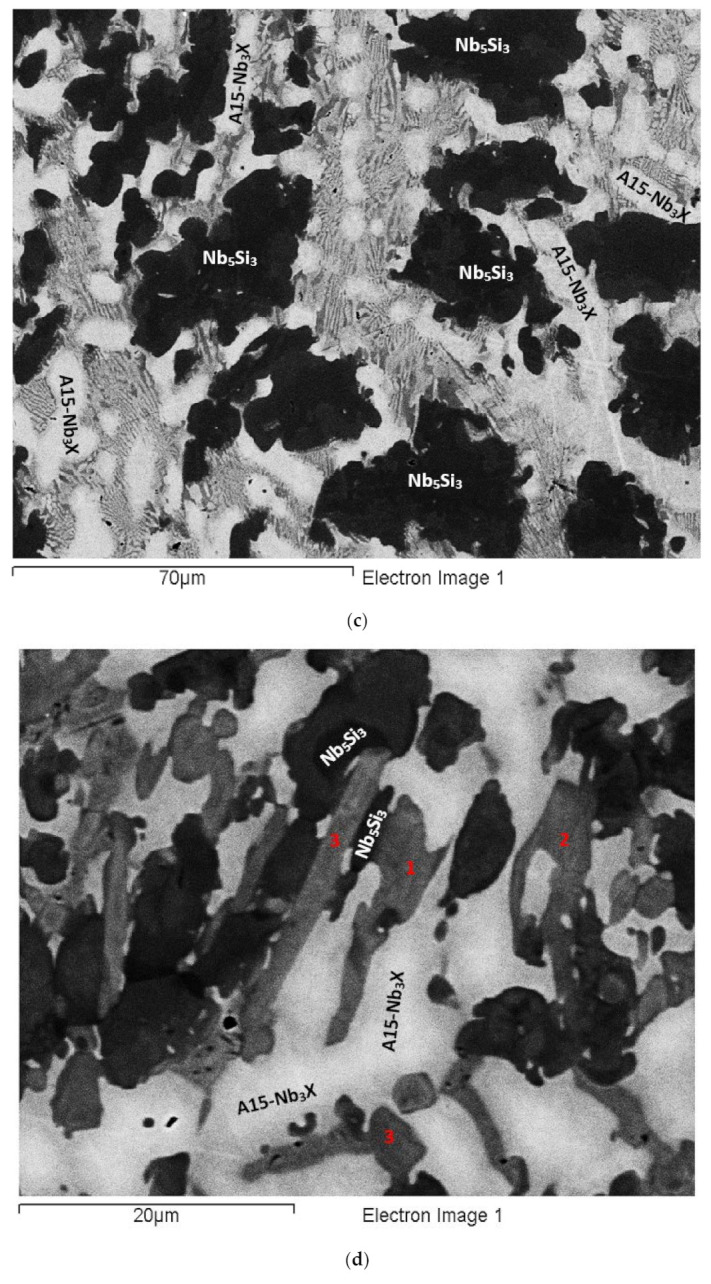

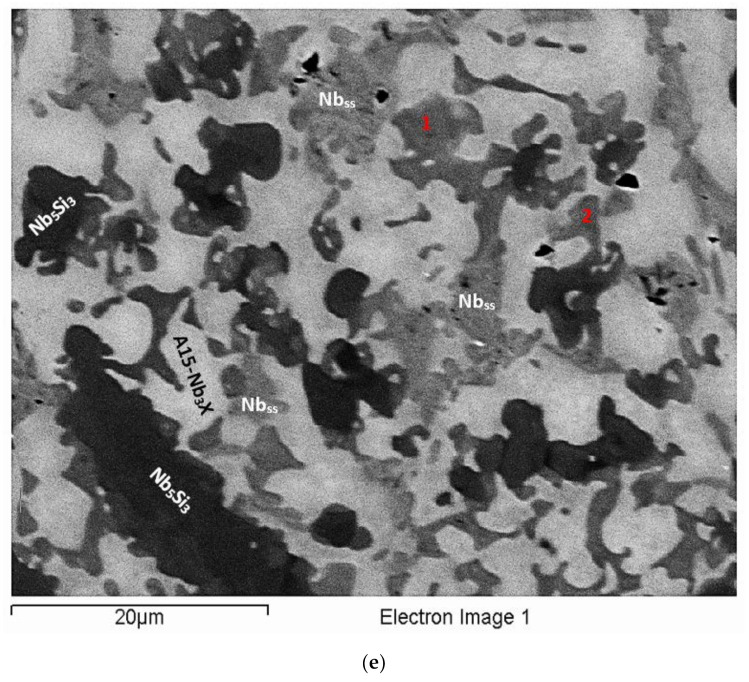
BSE images of EZ5-AC: (**a**) top, (**b**) and (**c**) bulk, and (**d**) and (**e**) bottom of the ingot. Note that contrast has been enhanced in (**a**,**b**,**d**,**e**) to show different phases, in particular the Nb_ss_ and the Hf-rich Nb_5_Si_3_ (numbers 1, 2 in (**e**)). See text for numbers.

The actual composition (at.%) of the alloy was 42.1Nb-23.9Ti-19.3Si-4.7Al-5.1Hf-4.9Sn. Compared with the nominal composition, the EZ5-AC was richer in Si. There was stronger macrosegregation of Si than Ti in the ingot (Table 2). Study of the microstructure was difficult owing to the partitioning of Hf and the presence of the A15-Nb_3_X (X = Al,Si,Sn), the contrast of which was similar to that of the Nb_ss_ (Figure 3). According to the XRD data (Figure S2 in the Supplemental Data), the phases in EZ5-AC were αNb_5_Si_3_, βNb_5_Si_3_, γNb_5_Si_3_, Nb_ss_, and Nb_3_Sn. There was Ti- and Hf-rich Nb_5_Si_3_ (Table 3 and Table S2 in the Supplemental Data). The Nb/(Ti + Hf) ratio of the Hf-rich Nb_5_Si_3_ would suggest the presence of γNb_5_Si_3_, in agreement with the XRD results.

Table S2 in the Supplemental Data gives the compositions of the phases that were confirmed using XRD and EPMA, namely the Nb_5_Si_3_, Nb_ss_, and A15-Nb_3_X (Table 3, Figure 3 and Figure S2 in the Supplemental Data). The latter was not observed in all parts of the ingot. The former two formed a eutectic consisting of the Nb_ss_ and Ti- and Hf-rich Nb_5_Si_3_ (Figure 3). This eutectic was not observed in all parts of the ingot. No HfO_2_ was observed in EZ5-AC. The Ti- and Hf-rich Nb_5_Si_3_ exhibited a brighter contrast than the Nb_5_Si_3_. This can be seen in Figure 3a (see areas indicated with the numbers 1 to 7), in Figure 3b (numbers 1 to 3), in Figure 3d (numbers 1 to 3), and in Figure 3e (numbers 1 and 2). The Ti- and Hf-rich Nb_5_Si_3_ in these areas had average Hf+Sn and Ti/(Hf+Sn) of, respectively, about 9.8 at.% and 2.3, compared with 6.2 at.% and 2.8 for the “normal” Nb_5_Si_3_.

The microstructure in the top of the ingot consisted of large Nb_5_Si_3_ grains surrounded by Nb_ss_ (Figure 3a), but A15-Nb_3_X was not observed. The Hf-rich areas of Nb_5_Si_3_ grains exhibited different contrasts of grey (see 1 to 7 in Figure 3a). The Nb_ss_ had Si/(Sn+Al) of about 0.2. In both the Nb_5_Si_3_ and the Hf-rich Nb_5_Si_3_, the Si + Sn + Al concentration was about 37.8 at.%. In the latter, the Nb/(Ti + Hf) ratio was 1.4, indicative of tetragonal Nb_5_Si_3_.

The microstructure in the bulk of the ingot was different than that in the top and consisted of Nb_5_Si_3_, Ti- and Hf-rich Nb_5_Si_3_, Nb_ss_, and A15-Nb_3_X phases (Figure 3b,c). Large Nb_5_Si_3_ grains were surrounded by a eutectic of the Ti- and Hf-rich Nb_5_Si_3_ and the Nb_ss_ (see Table S2 in Supplemental Data). In the Figure 3b, the Hf-rich Nb_5_Si_3_ in the eutectic is indicated with the numbers 1 to 3. The A15-Nb_3_X compound was adjacent to the eutectic. In more than one case, it grew directly next to Nb_5_Si_3_ (see Figure 3b,c). The Nb_ss_ in the eutectic exhibited a lighter grey contrast compared with the Nb_5_Si_3_ and Hf-rich Nb_5_Si_3_. The Nb_ss_ had Si/(Sn+Al) of about 0.2. The Si + Sn + Al concentration of the eutectic was 23.2 at.%, with Si/(Sn+Al) of about 1.4, and the corresponding values for the A15-Nb_3_X were about 20.6 at.% and 0.3, respectively. In the Nb_5_Si_3_ and the Ti- and Hf-rich Nb_5_Si_3_, the Si + Sn + Al concentration was about 37.6 and 38.5 at.%, respectively. The Ti- and Hf-rich Nb_5_Si_3_ had Nb/(Ti + Hf) of about 0.7, indicative of hexagonal Nb_5_Si_3_.

In the bottom of the ingot, the microstructure consisted of Nb_5_Si_3_ adjacent to or surrounded by Ti- and Hf-rich Nb_5_Si_3_, which was occasionally surrounded by A15-Nb_3_X, and by Nb_ss_ adjacent to or surrounded by the Ti- and Hf-rich Nb_5_Si_3_ (Figure 3d,e). The Ti- and Hf-rich Nb_5_Si_3_ grew from the Nb_5_Si_3_ with a distinct morphology (see Figure 3d). No eutectic was observed in the bottom of the ingot. The Nb_ss_ had Si/(Sn+Al) of about 0.2. The Si + Al + Sn content and the Si/(Sn + Al) ratio of the A15-Nb_3_X were about 20.3 at.% and 0.3, respectively. The Si + Sn + Al concentration in the Nb_5_Si_3_ was about 36.9 at.%, whereas in the Ti- and Hf-rich Nb_5_Si_3_, it was about 37.9 at.%. Furthermore, the Nb/(Ti + Hf) ratio of the Ti- and Hf-rich Nb_5_Si_3_ was about 0.8, which pointed to hexagonal Nb_5_Si_3_.

### 3.4. Heat-Treated EZ5-HT1 (1500 °C/100 h)

**Figure 4:**
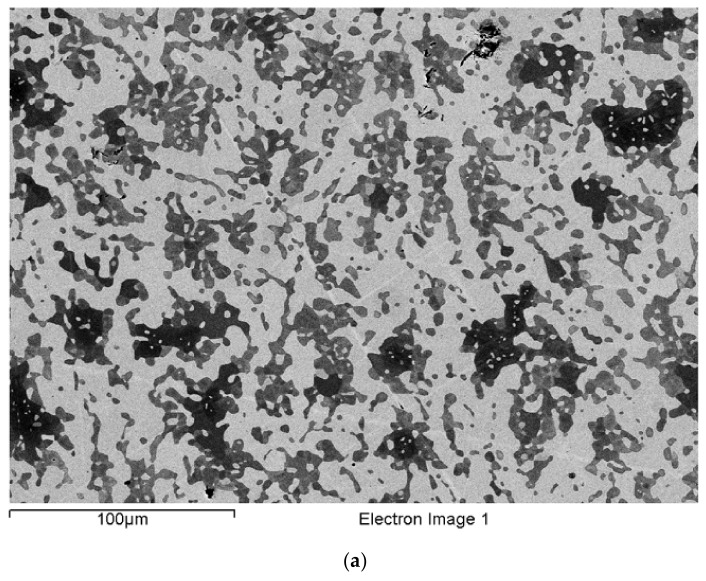

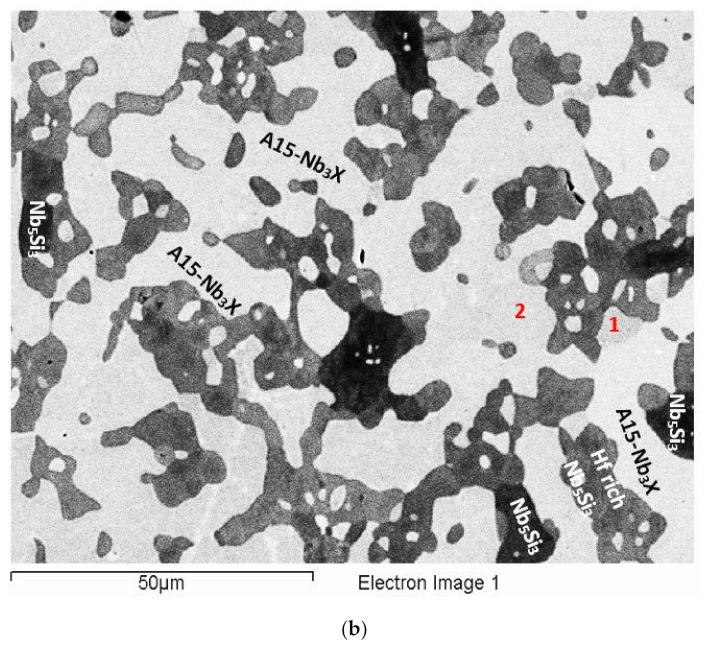
BSE images of the heat-treated alloy EZ5, (**a**) EZ5-HT1, and (**b**) EZ5-HT2. In (**b**), the numbers 1 and 2 indicate A15-Nb_3_X.

According to the XRD data (Figure S2 in the Supplemental Data) the microstructure consisted of the αNb_5_Si_3_, βNb_5_Si_3_, γNb_5_Si_3_ silicides and the Nb_3_Sn compound (Figure 4a). Nb_ss_ and HfO_2_ were not observed. The microstructure consisted of Nb_5_Si_3_ (dark contrast) and Hf-rich Nb_5_Si_3_ (grey contrast) surrounded by the A15-Nb_3_X (brighter contrast). Submicron particles precipitated in the Nb_5_Si_3_ and the Hf-rich Nb_5_Si_3_. The composition of these particles could not be determined owing to their size. The Si + Sn + Al concentration in the A15-Nb_3_X was about 19.7 at.% and the Si/(Sn+Al) ratio was about 0.3. In the Nb_5_Si_3_ and the Ti- and Hf-rich Nb_5_Si_3_ the Si + Sn + Al concentration was about 38.2 at.% and 39.3 at.%, respectively. The Nb/(Ti + Hf) ratio in the Ti- and Hf-rich Nb_5_Si_3_ was about 0.8, indicating hexagonal Nb_5_Si_3_.

### 3.5. Heat-Treated EZ5-HT2 (1500 °C/200 h)

The alloy EZ5 was given a second heat treatment at 1500 °C for an additional 100 h, in order to find out whether equilibrium had been achieved. The same specimen that was first heat treated for 100 h was given another 100 h heat treatment. According to the XRD data, the microstructure consisted of the same phases as in EZ5-HT1, namely αNb_5_Si_3_, βNb_5_Si_3_, γNb_5_Si_3_ silicides, and the Nb_3_Sn compound (Figure S2 in the Supplemental Data), and did not change compared with the EZ5-HT1. In some parts of the microstructure, the A15-Nb_3_X exhibited a small variation in contrast (see numbers 1 and 2 in the Figure 4b). Data for the chemical composition of the phases in EZ-HT2 are given in Table S2 in the Supplemental Data. The precipitates that were observed in both the Nb_5_Si_3_ and the Ti- and Hf-rich Nb_5_Si_3_ in EZ5-HT1 were still present (Figure 4b), but in the EZ5-HT2, the size of these precipitates in some Nb_5_Si_3_ grains made possible their chemical analysis. The results confirmed that the precipitates were indeed the A15-Nb_3_X compound. The Si + Sn + Al concentration of the Nb_3_X was about 19.6 at.% with an Si/(Sn + Al) ratio of about 0.3. The S + Sn + Al concentration of the Nb_5_Si_3_ and the Ti- and Hf-rich Nb_5_Si_3_ was about 37.7 at.% and 38.4 at%, respectively. Both values were lower than the corresponding ones in EZ5-HT1. Similar to the EZ5-HT1, the Nb/(Ti + Hf) ratio of the Ti- and Hf-rich Nb_5_Si_3_ phase was about 0.8, which is indicative of hexagonal Nb_5_Si_3_.

### 3.6. As-Cast EZ6

**Figure 5:**
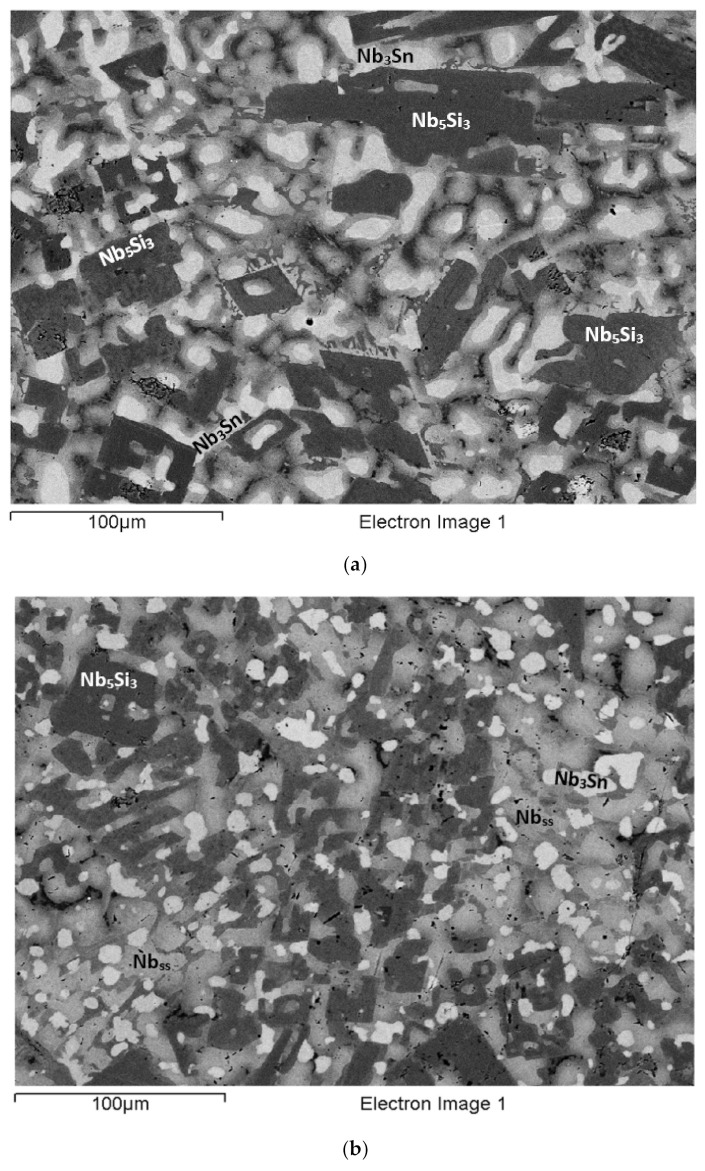
BSE images of the microstructure of EZ6-AC: (**a**) bulk and (**b**) bottom of ingot. Contrast has been enhanced to show all phases. The A15-Nb_3_X compound is shown as Nb_3_Sn. See Figure 6 for further details of the microstructure.

**Figure 6:**
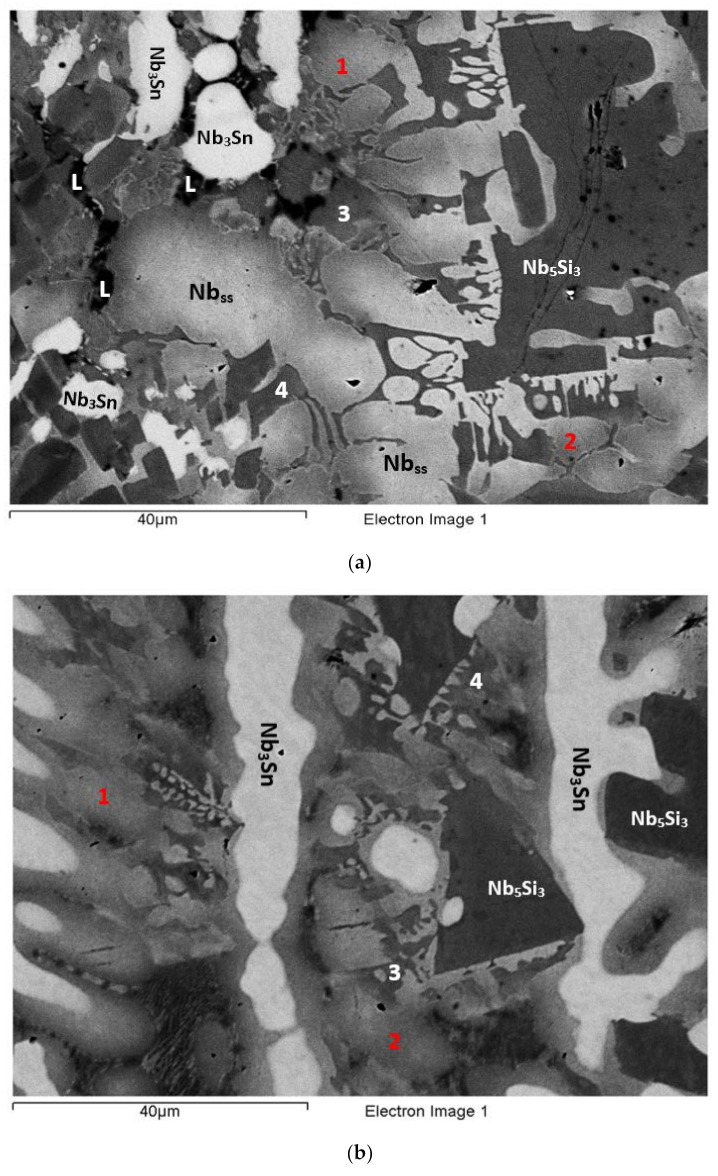

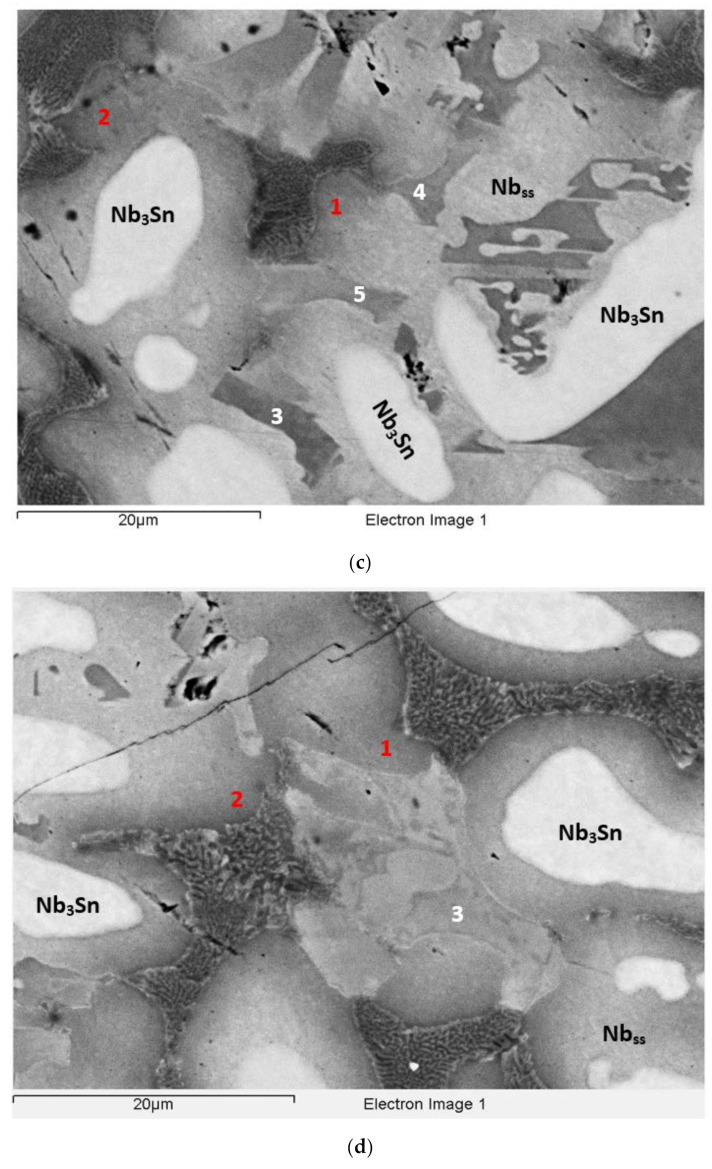

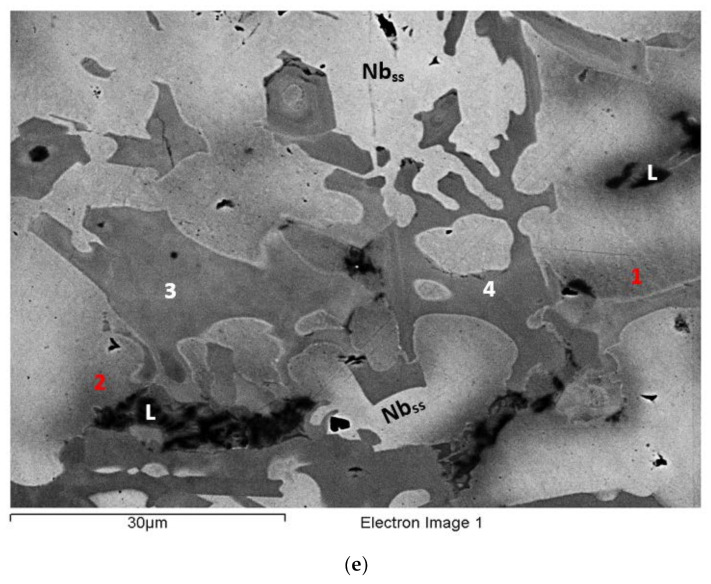
BSE images showing details of the microstructure of EZ6-AC: (**a**) top, (**b**–**d**) bulk, and (**e**) bottom of the ingot. Numbers 1 and 2 show Ti-rich Nb_ss_; numbers 3, 4, and 5 show Ti- and Hf-rich Nb_5_Si_3_. The NbCr_2_ Laves phase is indicated with L, and the A15-Nb_3_X is shown as Nb_3_Sn.

The actual alloy composition (at.%) was 41Nb-24.2Ti-19.7Si-4.8Cr-5.3Hf-5Sn. Compared with the nominal composition, the EZ6-AC ingot was richer in Si. There was macrosegregation of Si, Ti, and Cr (Table 2), which was particularly strong for Si and Ti. Study of the microstructure was difficult owing to the partitioning of Hf and Sn (Figure 5 and Figure 6). The XRD data (Figure S3 in the Supplemental Data) indicated the presence of Nb_ss_, Nb_3_Sn, αNb_5_Si_3_, βNb_5_Si_3_, γNb_5_Si_3_, C14-NbCr_2_ Laves, and HfO_2_. The EPMA confirmed the Nb_ss_, A15-Nb_3_X, Nb_5_Si_3_, and C14-NbCr_2_ Laves phase and eutectic of Nb_ss_ and NbCr_2_ (Table 3).

The microstructure near the top of the ingot (Figure 6a) was similar to that in the bulk (Figure 5a) and consisted of bulky, faceted Nb_5_Si_3_ (dark contrast) surrounded by Nb_ss_ (grey contrast), A15-Nb_3_X (very bright contrast), and NbCr_2_ Laves (very dark contrast). The A15-Nb_3_X was surrounded by the Nb_ss_ or by the Laves phase and Ti- and Hf-rich Nb_5_Si_3_ (Figure 6a). The Laves phase was formed in between A15-Nb_3_X grains or Ti-rich Nb_ss_ and Ti- and Hf-rich Nb_5_Si_3_ (Figure 6a). The Ti-rich Nb_ss_ exhibited a darker contrast compared with the “normal” Nb_ss_ (numbers 1 and 2 in Figure 6a). The Ti- and Hf-rich Nb_5_Si_3_ (numbers 3 and 4 in Figure 6a) was separate from the “normal” Nb_5_Si_3_.

In the top of the ingot, the Si+Sn concentration in the Nb_ss_ was about 8.9 at.%, and the Si/Sn ratio was about 0.3, while the corresponding values for the Ti-rich Nb_ss_ were about 9.0 at.% and 0.2, respectively. In the A15-Nb_3_X, the Si+Sn content and Si/Sn ratio was about 18.6 at.% and 0.4. The Si+Sn concentration in the Nb_5_Si_3_ and the Ti- and Hf-rich Nb_5_Si_3_ was about 37.4 at.% and 38.3 at.%, respectively, and the Nb/(Ti + Hf) ratio of the latter was about 0.7, which corresponds to hexagonal γNb_5_Si_3_. The Si + Sn + Cr concentration of the Laves phase was about 58.9 at.%. The Laves phase formed a very fine eutectic with the Nb_ss_. In this part of the ingot, the average composition of the eutectic was 23.3Nb-24.7Ti-7.4Si-35.7Cr-6.0Hf-2.9Sn (at.%), with Si+Sn+Cr of about 46.0 at.%.

In the bulk, the microstructure consisted of the same phases as near the top (Figure 5a and Figure 6b–d), but the vol.% of the A15-Nb_3_X was higher. The Si+Sn concentration in both the Nb_ss_ and the Ti-rich Nb_ss_ was slightly reduced compared with the top (about 8.4 at.%), and both had the same Si/Sn ratio (about 0.3). In the A15-Nb_3_X, the Si+Sn content was about 18.7 at.%, and Si/Sn about 0.4. The Si+Sn concentration in the Nb_5_Si_3_ and the Ti- and Hf-rich Nb_5_Si_3_ was the same (about 38.1 at.%), but the Nb/(Ti + Hf) ratio of the latter silicide was about 1.4. The Si + Sn + Cr concentration of the Laves phase was about 51.1 at.%. There was also a Nb_ss_+NbCr_2_ eutectic, with average composition 21.2Nb-32.6Ti-11.6Si-22.6Cr-8.2Hf-3.8Hf (at.%), and Si+Sn+Cr of about 38.0 at.%. The eutectic was formed near Ti- and Hf-rich Nb_5_Si_3_. Some eutectic areas contained chunks of the Laves phase (e.g., see eutectic on the left-hand side of number 1 in Figure 6c).

The microstructure in the bottom of the ingot consisted of the same phases as near the top and bulk (Figure 5b and Figure 6e), but the vol.% of the A15-Nb_3_X was significantly reduced. Furthermore, there was no evidence of the NbCr_2_ + Nb_ss_ eutectic. The Si+Sn concentration and Si/Sn ratio in the Nb_ss_ and Ti rich Nb_ss_ were about 8.6 at.% and 0.3, and 8.8 at.% and 0.2, respectively. The Si+Sn content and the Si/Sn ratio of the A15-Nb_3_X were about 18.4 at.% and 0.4, respectively. In the Nb_5_Si_3_ and the Ti- and Hf-rich Nb_5_Si_3_, the Si+Sn concentration was essentially the same (about 38.2 at.%), and the Nb/(Ti + Hf) ratio in the latter was about 0.8, which corresponds to hexagonal γNb_5_Si_3_, as was the case near the top of the ingot. The Cr + Si + Sn concentration in the Laves phase was about 56.8 at%. The Laves phase was surrounded by either Ti-rich Nb_ss_ or Ti- and Hf-rich Nb_5_Si_3_.

### 3.7. Heat-Treated EZ6-HT1 (1500 °C/100 h)

**Figure 7:**
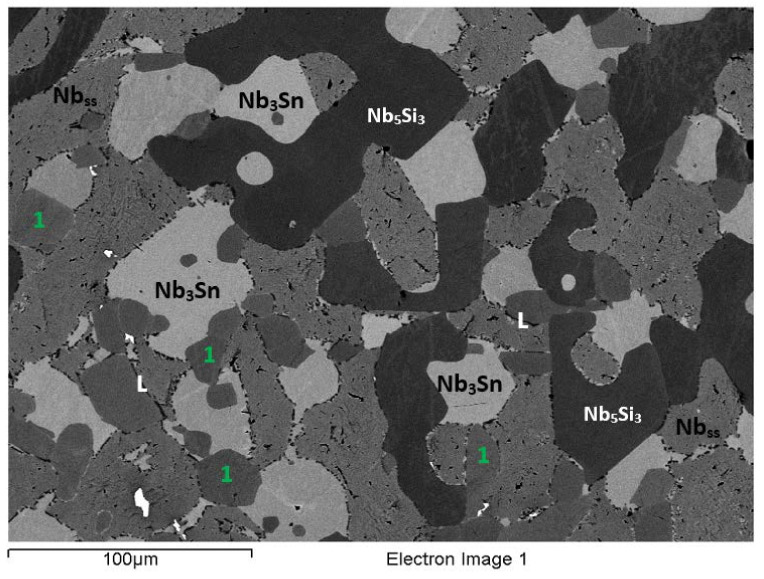
BSE image of the microstructure of EZ6-HT1. The Hf-rich Nb_5_Si_3_ is indicated with the number 1, and thin strips of Laves phase at the interface between Nb_ss_ and the aforementioned silicide are shown with L. Bright phase on the left-hand side of number 1 in the bottom left-hand corner is HfO_2_. The A15-Nb_3_X is shown as Nb_3_Sn.

After the heat treatment at 1500 °C for 100 h, there was liquation in EZ-HT1. The microstructure had significantly coarsened compared with EZ6-AC (compare Figure 7 with Figure 5a) and exhibited features similar to those reported for the heat-treated alloy Nb-24Ti-18Si-8Cr-4Al (alloy KZ2-HT1 (1500 °C/100 h), in [21]), which also had undergone liquation.

Data for the microstructure of EZ6-HT1 is given in Table S3 and in Figure S3 in the Supplemental Data, and in Figure 7. According to the XRD data, αNb_5_Si_3_, βNb_5_Si_3_, γNb_5_Si_3_, Nb_3_Sn, Nb_ss_, HfO_2_, and C14-NbCr_2_ Laves phase were present. There were no Ti-rich areas in the Nb_ss_, but there was still Ti- and Hf-rich Nb_5_Si_3_. Precipitation of the Laves phase had occurred not only at the interfaces of the Nb_ss_ with the A15-Nb_3_X or Hf-rich Nb_5_Si_3_, but also within the Nb_ss_ grains, as shown in Figure 7. The needle-like Laves precipitates that were formed within and around the grains of the Nb_ss_ exhibited similar morphology to those observed in the KZ2-HT1 alloy that had experienced liquation [21]. After this heat treatment, a very small volume fraction of HfO_2_ was formed. The Si+Sn content and Si/Sn ratio of the Nb_ss_ were about 7.3 at.% and 0.1, respectively. The corresponding values for the A15-Nb_3_X were about 18.5 at.% and 0.3, respectively. The Si+Sn concentration in the Nb_5_Si_3_ and the Ti- and Hf-rich Nb_5_Si_3_ was about 38.1 at.% and 38.8 at.%, respectively. The Nb/(Ti + Hf) ratio in the Ti- and Hf-rich Nb_5_Si_3_ was about 0.7, indicating hexagonal γNb_5_Si_3_. The Cr+Si+Sn concentration in the Laves phase was about 54.1 at.%. The latter value should be viewed with caution owing to the size of the Laves phase in EZ6-HT1.

### 3.8. Heat-Treated EZ6-HT2 (1200 °C/100 h)

Given the liquation that occurred in EZ6-HT1, the alloy was heat treated at 1200 °C for 100 h. According to the XRD data (Figure S3 in the Supplemental Data), the microstructure consisted of αNb_5_Si_3_, βNb_5_Si_3_, γNb_5_Si_3_, Nb_3_Sn, Nb_ss_, HfO_2_, and C14-NbCr_2_ Laves phase. Table S3 in the Supplemental Data summarizes the EPMA data for the phases that were confirmed with XRD and EPMA. In other words, the microstructure of EZ6-HT2 contained Nb_ss_, A15-Nb_3_X, Nb_5_Si_3_, HfO_2_, and C14-NbCr_2_ Laves phase (Table 3).

**Figure 8:**
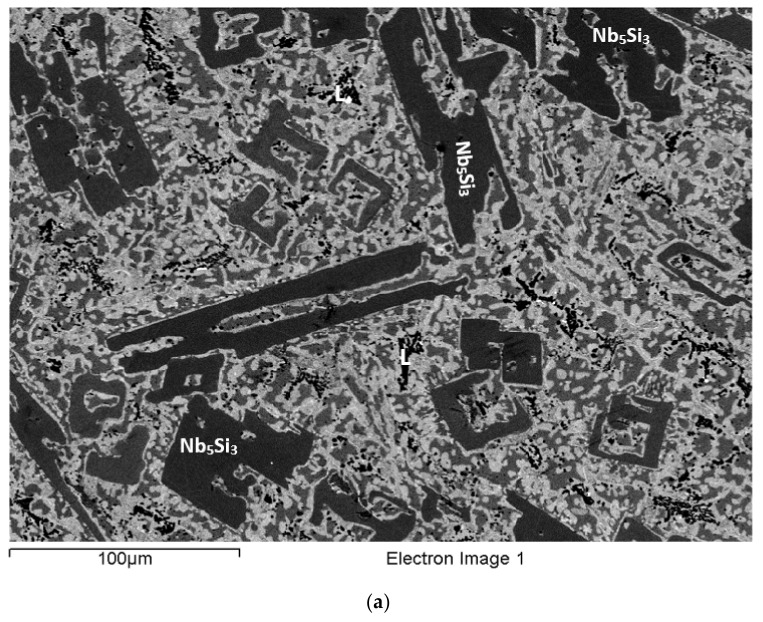

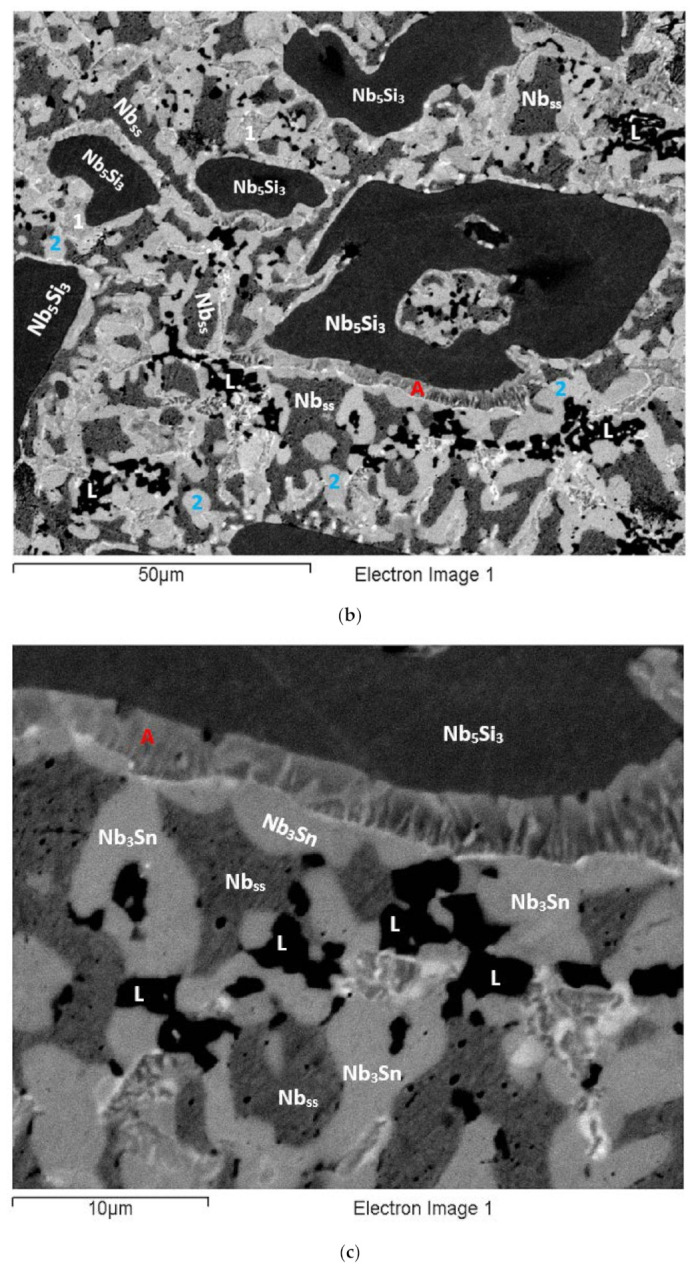

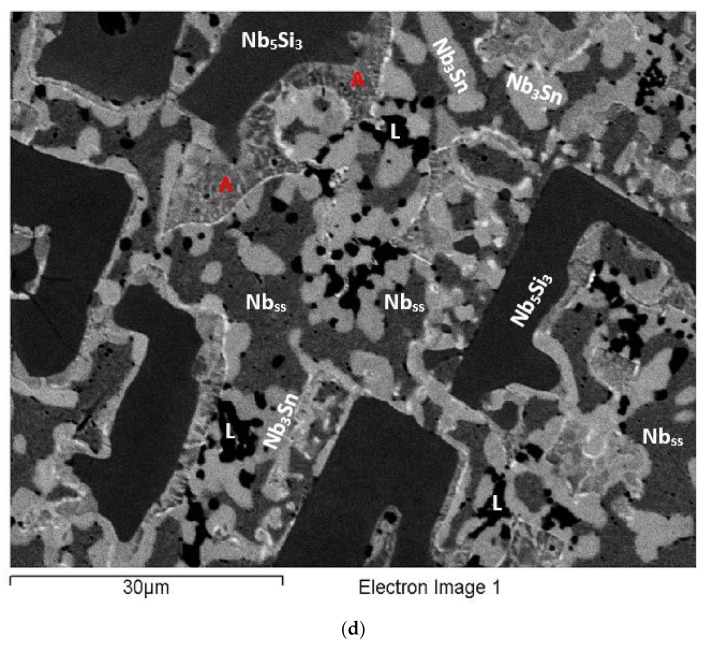
(**a**–**d**) BSE images of the microstructure of EZ6-HT2. In (**a**–**d**), the Laves phase is indicated with L. In (**b**), the Hf-rich Nb_5_Si_3_ and the A15-Nb_3_X are indicated with the numbers 1 and 2, respectively, while (**c**) shows details of the microstructure near the large Nb_5_Si_3_ grain in (**b**). For A in (**b**–**d**), see text. In (**b**–**d**), the contrast has been enhanced to show the microstructure A.

The microstructure of EZ6-HT2 was significantly different compared with EZ6-HT1 (Figure 8a). There was no Ti-rich Nb_ss_, but Ti- and Hf-rich Nb_5_Si_3_ was still present. The vol.% of the Nb_ss_ was significantly reduced compared with EZ6-AC (Table 1). The Nb_5_Si_3_ was still facetted (Figure 8a). The average Si+Sn concentration and the Si/Sn ratio of the Nb_ss_ were about 4.6 at.% and 0.2, and the corresponding values for the A15-Nb_3_X were about 18.2 at.% and 0.2, respectively. There was growth of the Laves phase (Figure 8), of which the average Cr + Si + Sn concentration was about 59.7 at.%. The Si+Sn concentration in Nb_5_Si_3_ was about 37.4 at.%. The Ti- and Hf-rich Nb_5_Si_3_ was distinct from the “normal” Nb_5_Si_3_, and its contrast was very close to that of the A15-Nb_3_X (Figure 8b).

Figure 8b,c,d shows that adjacent to “normal” Nb_5_Si_3_ silicide grains, but not surrounding the whole Nb_5_Si_3_ grain, there was a microstructure that had formed after this heat treatment (indicated with A in Figure 8, and referred to below as microstructure A). The average composition of the “normal” Nb_5_Si_3_ grains, next to which the microstructure A was formed, was 42.5Nb-13.9Ti-36.7Si-0Cr-5Hf-1.8Sn (at.%), with Si + Sn = 38.5, Si/Sn = 20.8, Ti/Hf = 2.8, Ti + Hf = 19 at.%, and Nb/(Ti + Hf) = 2.25 (i.e., tetragonal silicide). Note that there was no Cr in these silicide grains. The phases in microstructure A exhibited different contrasts, and possibly formed a “lamellar” microstructure (Figure 8d). The contrast of the phases was similar to that of the phases in the microstructure of EZ6-HT2. Separate analysis of the composition of each of the phases was not possible owing to their size. Instead, large area analysis of microstructure A gave its average composition as 25Nb-27.1Ti-33.8Si-1.2Cr-11.2Hf-1.7Sn (at.%), with Si + Sn = 35.5 at.%, Si/Sn = 19.9, Ti/Hf = 2.4, Ti + Hf = 38.3 at.%, and Nb/(Ti + Hf) = 0.66.

The interface of microstructure A with the A15-Nb_3_X was often decorated with fine particles exhibiting white contrast, that is, a bright contrast phase (BCP) (Figure 8b–d). The average composition of the BCP was 18.6Nb-25.7Ti-39.8Si-0.6Cr-14.7Hf-0.65Sn (at.%), with Si + Sn = 40.45 at.%, Si/Sn = 61.9, Ti/Hf = 1.75, Ti + Hf = 40.4 at.%, and Nb/(Ti + Hf) = 0.5. Next to the BCP particles, the A15-Nb_3_X (Figure 8c) had average composition 45.6Nb-31.1Ti-2.7Si-3.1Cr-2Hf-15.6Sn (at.%), with Si + Sn = 18.3 at.%, Si/Sn = 0.2, Ti/Hf = 15.9, Ti + Hf = 33 at.%, and Nb/(Ti + Hf) = 1.4; the average composition of the Nb_ss_ was 55.1Nb-31.1Ti-0.5Si-7.1Cr-2.3Hf-3.9Sn (at.%), with Si + Sn = 4.4 at.%, Si/Sn = 0.3, Ti/Hf = 13.3, Ti + Hf = 33.4 at.%, and Nb/(Ti + Hf) = 1.65; and the average composition of the Laves phase was 22.3Nb-12.5Ti-10.1Si-48.7Cr-6Hf-0.4Sn (at.%), with Si + Sn = 10.5 at.%, Si/Sn = 25.3, Ti/Hf = 2.1, Ti + Hf = 18.6 at.%, Nb/(Ti + Hf) = 1.2, and Cr + Si + Sn = 59.2 at.%.

### 3.9. As-Cast EZ8

**Figure 9:**
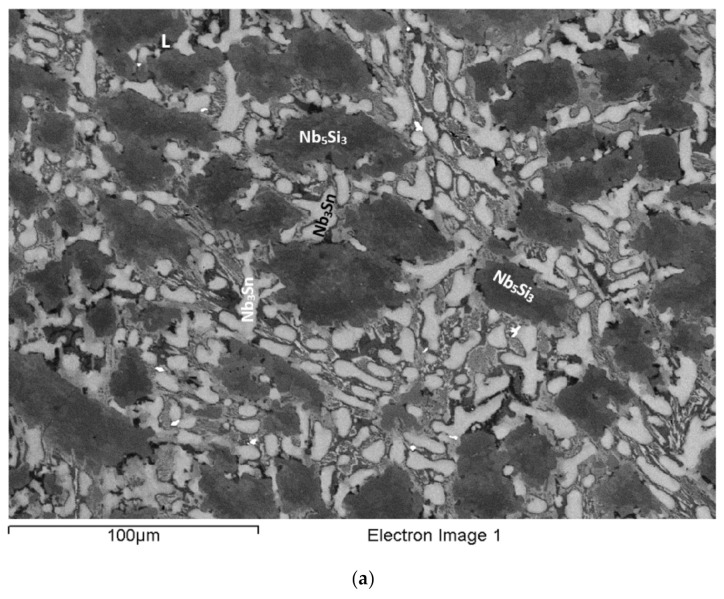

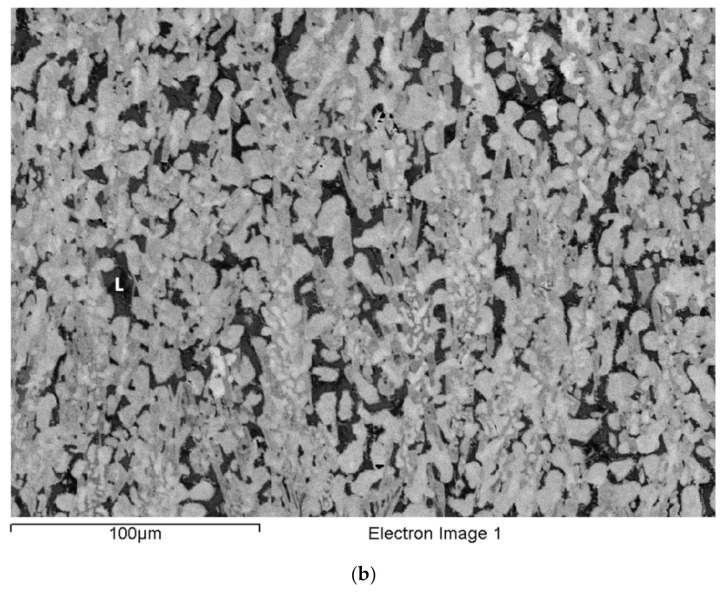
BSE images (**a**) of the bulk and (**b**) of the bottom of the as cast ingot of EZ8. Laves phase is indicated with L.

**Figure 10:**
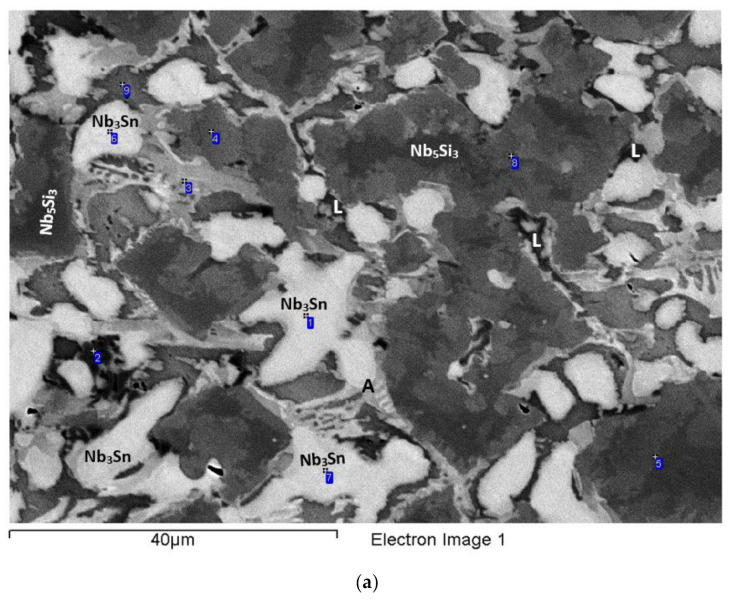

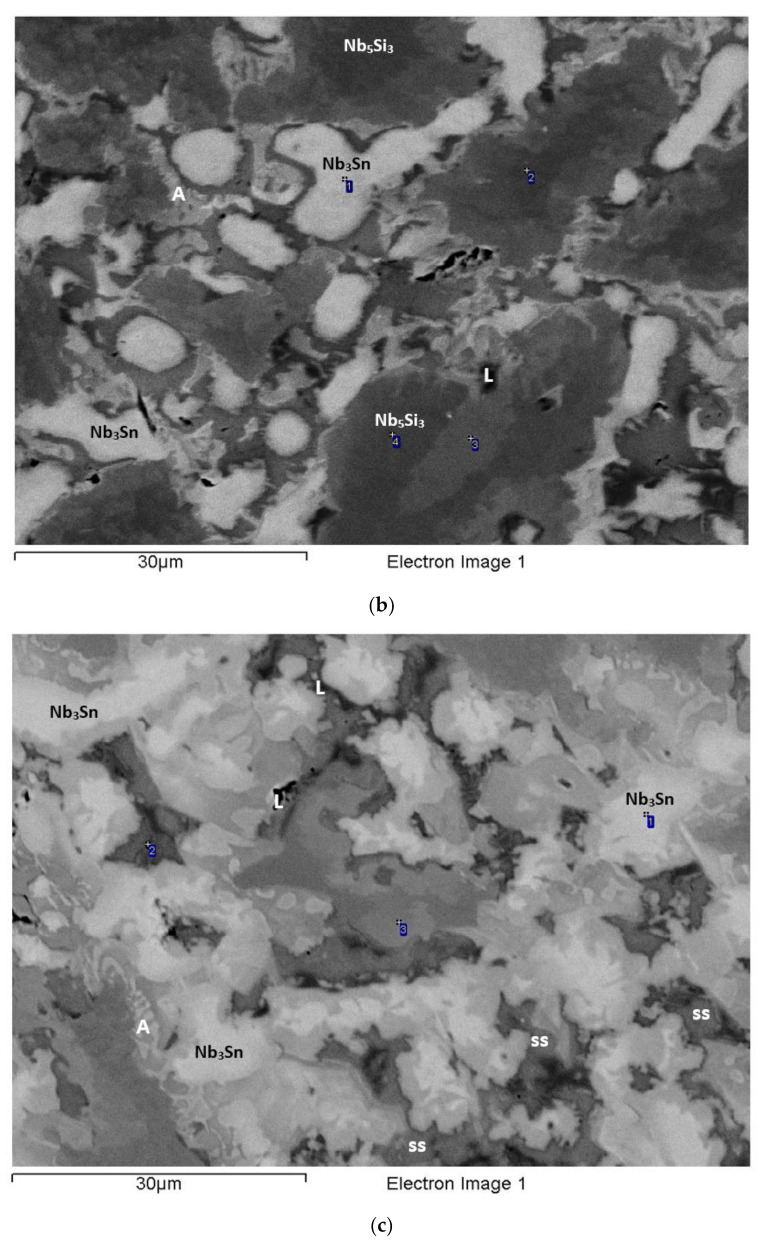

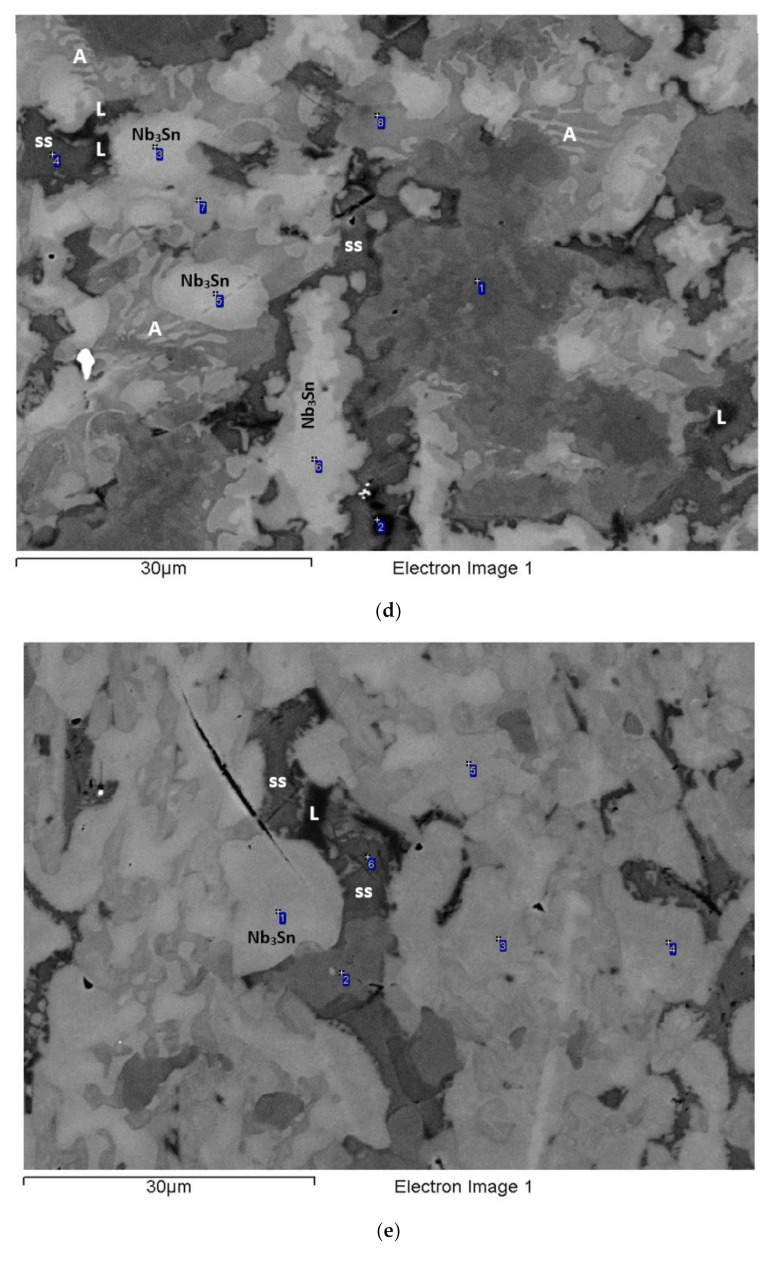

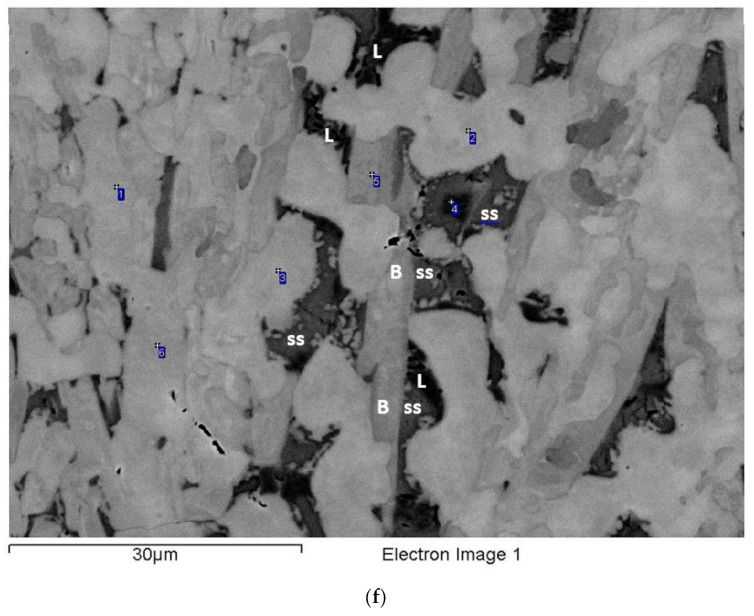
BSE images of the top (**a**,**b**), bulk (**c**,**d**), and bottom (**e**,**f**) of the ingot of EZ8-AC showing details of the microstructure. The Laves phase is indicated with L, and the Nb_ss_ with ss. The A15-Nb_3_X is shown as Nb_3_Sn. In (**a**), the Nb_3_Sn is shown with the numbers 1, 6, and 7; the Ti- and Hf-rich Nb_5_Si_3_ with the numbers 3, 4, 5, 8, and 9; and the Laves phase with 2. In (**b**), the Nb_3_Sn is shown with the number 1, and the Ti- and Hf-rich Nb_5_Si_3_ with 3. In (**c**), the Nb_3_Sn is shown with the number 1, and the Ti- and Hf-rich Nb_5_Si_3_ with 3. In (**d**), the Nb_ss_ is shown with the number 4; Nb_3_Sn with 3, 5, and 6; Nb_5_Si_3_ with 1; Ti- and Hf-rich Nb_5_Si_3_ with 7 and 8; the Laves phase with 2; and HfO_2_ is the very bright phase on the left-hand side of the number 5. In (**e**), Nb_3_Sn is shown with the numbers 1, 3, 4, and 5; the Nb_ss_ with 6; and the Nb_5_Si_3_ with 2. In (**f**), the Nb_3_Sn is shown with the numbers 1, 2, 3, and 6; the Laves phase with 4; and the Ti- and Hf-rich Nb_5_Si_3_ with 5. For the areas A in (**b**–**d**) and the area B in (**f**), see text.

The actual composition (at.%) of the alloy was 36.9Nb-24.6Ti-17.8Si-4.9Al-5.1Cr-5.4Hf-5.3Sn. The macrosegregation of Si was the highest of all the alloys. In addition, there was macrosegregation of Cr and Ti (Table 2). Typical microstructures are shown in Figure 9 and Figure 10. Study of the microstructure of EZ8 was difficult owing to the partitioning of Hf and the formation of A15-Nb_3_X, the contrast of which was similar to that of the Nb_ss_. According to the XRD data (Figure S4 in Supplemental Data), the microstructure consisted of αNb_5_Si_3_, βNb_5_Si_3_, γNb_5_Si_3_, Nb_3_Sn, Nb_ss_, HfO_2_, and C14-NbCr_2_ Laves phase. The analysis data (Table S4 in Supplemental Data) supports the presence of γNb_5_Si_3_, as the Nb/(Ti + Hf) ratio of the silicide was less than one. The HfO_2_ was observed only in the bulk of the ingot, where it had formed at a very small vol.%.

The microstructure in the top of the ingot was similar to that in the bulk. It consisted of large Nb_5_Si_3_ grains that were surrounded by Ti- and Hf-rich Nb_5_Si_3_. There was strong microsegregation of Hf in the Nb_5_Si_3_ that exhibited different contrasts (Figure 10a,b). Adjacent to the Ti- and Hf-rich Nb_5_Si_3_, Nb_ss_ was formed. The A15-Nb_3_X was formed adjacent to the Nb_ss_, and it was often completely surrounded by it. In many parts of the microstructure, a complicated network of Ti- and Hf-rich Nb_5_Si_3_ and A15-Nb_3_X had grown adjacent to Nb_5_Si_3_. The microstructure in these areas (denoted as A in Figure 10a,b) seemed to have grown directly from the Nb_5_Si_3_, and even though it was coarser, it resembled a lamellar microstructure.

The Laves phase was often observed at the interface of the Nb_ss_ with the A15-Nb_3_X. The average Si + Al + Sn concentration in the Nb_ss_ was about 13.1 at.% with an Si/(Sn + Al) ratio of about 0.2. The corresponding values for the A15-Nb_3_X were about 20.9 at.% and 0.3, respectively. In the Nb_5_Si_3_, the Si + Al + Sn concentration was about 35.8 at.%, and it was about 37.2 at.% for the Ti- and Hf-rich Nb_5_Si_3_. The Nb/(Ti + Hf) ratio of the latter was about 0.7, indicating γNb_5_Si_3_. The Si + Sn + Al + Cr concentration of the Laves phase was about 47.9 at.%. There was a Nb_ss_+NbCr_2_ eutectic in the areas close to the top of the ingot.

The microstructure in the bulk of the ingot was similar to the one observed in the top (see Figure 9a and Figure 10c,d). The areas between the A15-Nb_3_X and the Ti- and Hf-rich Nb_5_Si_3_ (denoted as A) that were observed in the top of EZ8-AC were also present in this part of the ingot. The average Si + Al + Sn concentration in the Nb_ss_ was about 12.9 at.%, the Si/(Sn+Al) ratio was about 0.2, and the corresponding values for the A15-Nb_3_X were about 21.1 at.% and 0.3, respectively. In the Nb_5_Si_3_, the Si + Al + Sn concentration was about 38.3 at.%. The respective concentration in the Ti- and Hf-rich Nb_5_Si_3_ was about 37.2 at.%, with Nb/(Ti + Hf) of about 0.7. The average Si + Al + Sn + Cr concentration in the Laves phase was about 50 at.%. There was Nb_ss_ + NbCr_2_ eutectic in some areas of the bulk of EZ8-AC.

The microstructure in the bottom of the ingot was different compared with the top and bulk. The vol.% of the Nb_5_Si_3_ was significantly decreased. The Si concentration in the bottom of the ingot was about 14.7 at.%, that is, significantly lower than the nominal composition of Si in the alloy (Table S4 in Supplemental Data). Furthermore, the microstructure shown in Figure 9b and Figure 10e,f was distinctively finer compared with that observed in the top and bulk, and consisted of Nb_5_Si_3_, Ti- and Hf-rich Nb_5_Si_3_, A15-Nb_3_X, Nb_ss_, and the C14-NbCr_2_ Laves phase. A sharp (flat) interface was formed between the Nb_5_Si_3_ and Nb_ss_. The areas where this morphology was evident are denoted as B in Figure 10f. The average Si + Sn + Al concentration in the Nb_ss_ was about 13.0 at.%, with Si/(Sn + Al) of about 0.1. The corresponding values for the A15-Nb_3_X were about 20.6 at.% and 0.3, respectively. The Si + Sn + Al concentration in the Nb_5_Si_3_ and the Ti- and Hf-rich Nb_5_Si_3_ was about 37.0 at.% and 38.5 at.%, respectively. As was the case in other areas of EZ8-AC, the Ti- and Hf-rich Nb_5_Si_3_ had Nb/(Ti + Hf) of about 0.7, indicating hexagonal γNb_5_Si_3_. The Si + Sn + Al + Cr concentration in the Laves phase was about 59.6 at.%. The Nb_ss_ + NbCr_2_ eutectic was also observed.

### 3.10. Heat-Treated EZ8 (EZ8-HT)

**Figure 11:**
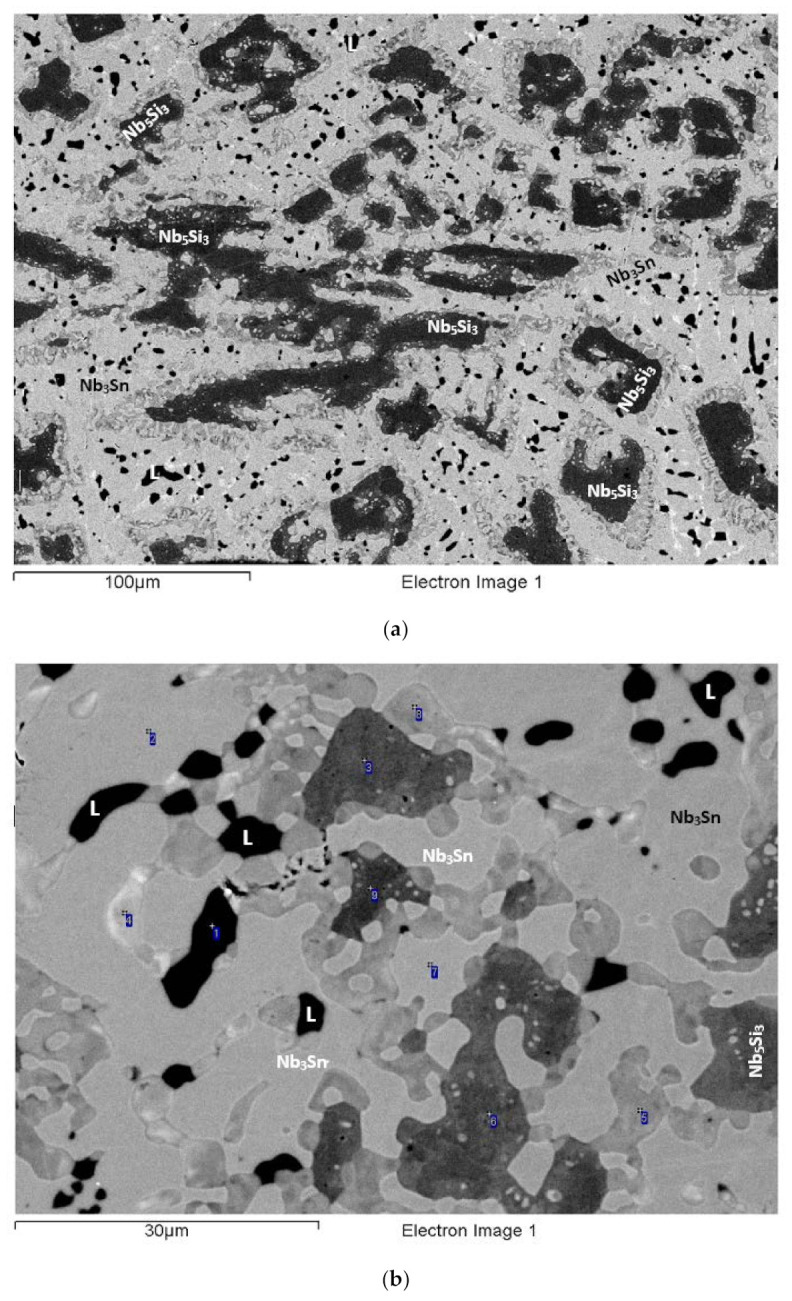
BSE images of the microstructure of EZ8-HT. In (**a**,**b**) the Laves phase is indicated with L. The A15-Nb_3_X is shown as Nb_3_Sn. In (**b**) the Laves phases is shown with the number 1, the Nb_3_Sn with 2, 7, the Nb_5_Si_3_ with 3, 6, 9, the Hf-rich Nb_5_Si_3_ with 4, 5, 8. The brighter contrast areas to the left of number 4 are Hf-rich Nb_5_Si_3_ with Nb/(Ti + Hf) = 0.6.

After heat treatment at 1300 °C for 100 h, the micro-structure of EZ8-HT consisted of αNb_5_Si_3_, βNb_5_Si_3_, γNb_5_Si_3_ (Nb/(Ti + Hf) about 0.7), A15-Nb_3_X, C14-NbCr_2_ Laves phase, and HfO_2_ (Figure S4 in the Supplemental Data). The microstructure is shown in Figure 11 and consisted of large Nb_5_Si_3_ grains surrounded by a network of interpenetrating Ti- and Hf-rich Nb_5_Si_3_ and A15-Nb_3_X. Nb_ss_ was not observed. The C14-NbCr_2_ Laves phase had grown significantly larger and formed distinct areas at the boundaries of either the Ti- and Hf-rich Nb_5_Si_3_ or the A15-Nb_3_X phase. The average Si + Sn + Al concentration in the A15-Nb_3_X was about 19.7 at%, with Si/(Sn + Al) of about 0.2. In the Nb_5_Si_3_ and the Ti- and Hf-rich Nb_5_Si_3_ phases, the Si + Sn + Al concentration was about 36.7 at.% and 38.1 at.%, respectively. The Laves phase had average Si + Sn + Al + Cr of about 62 at.%. A15-Nb_3_X had precipitated in the Nb_5_Si_3_.

### 3.11. Hardness

The hardness of the alloys and the hardness of the A15-Nb_3_X, Nb_ss_, and Nb_5_Si_3_ phases are given in Table 1 and Table 4, respectively. The hardness of the alloy EZ2 did not change after the heat treatment, whereas the hardness of the alloys EZ5, EZ6, and EZ8 increased. In the case of the alloy EZ2, there was a slight reduction in the hardness of Nb_5_Si_3_ and a more significant reduction in the hardness of Nb_ss_. The hardness of Nb_ss_ in EZ6 also decreased after heat treatment. Regarding the alloys EZ5, EZ6, and EZ8, the hardness of A15-Nb_3_X increased after heat treatment. However, the hardness of Nb_5_Si_3_ increased in EZ5 and decreased in EZ6 and EZ8.

### 3.12. Lattice Parameter of Nb_ss_

The lattice parameter of the bcc Nb_ss_ in the alloys of this work is given in Table 5, where data for other comparable alloys is included (see discussion).

## 4. Discussion

### 4.1. Macrosegregation

Macrosegregation of solute additions is common in cast RM(Nb)ICs [40]. The macrosegregation of Si (MACSi) in Nb-18Si silicide-based alloys with/without the addition of Al, Cr, Hf, Sn, or Ti is compared in Table 6. The data show the following:

(i) In the absence of Sn,
  (a) the synergy of Ti with simultaneous additions of Al and Cr decreased MACSi (compare the alloys KZ7, KZ4 and KZ5);
  (b) the effect of the synergy of Ti with Hf on the increase of MACSi was stronger than the synergy of Ti with Cr or Al individually (compare the alloys YG3, KZ7, KZ4), and the same was the case when Ti was in synergy with Al, Cr, and Hf simultaneously (compare the alloys KZ5 and JN1);
(ii) In the absence of both Ti and Sn,
  (c) the synergy of Hf with Al slightly increased MACSi compared with the synergy of Hf with Cr (alloys YG2 and YG1);
(iii) With the addition of 5 at.% Sn but with no Ti in the alloy,
  (d) the MACSi increased when Hf and Sn were in synergy with Cr or Al (compare the alloys EZ3, EZ4, EZ1, EZ7, NV9);
(iv) With the simultaneous addition of 24 at.% Ti and 2 at.% Sn,
  (e) the MACSi decreased when said elements were in synergy with Al and/or Cr (alloys ZX7, ZX3, ZX5), a similar trend as with (ia);
(v) With the simultaneous addition of 24 at.% Ti and 5 at.% Sn but in the absence of Hf,
  (f) the MACSi increased significantly when Al and/or Cr were in synergy (alloys ZX8, ZX4, ZX6), which is the opposite behaviour compared with the 2 at.% Sn addition, see (ive);
(vi) With the simultaneous addition of 24 at.% Ti and 5 at.% Sn and 5 at.% Hf,
  (g) the MACSi increased significantly when Al and/or Cr were in synergy with the said elements (alloys EZ8, EZ6, ZX6, EZ2, EZ5).

In other words, (1) the synergy of 5 at.% Hf with 5 at.% Al, 5 at.% Cr, 5 at.% Sn, and 24 at.% Ti slightly reduced MACSi, compared with the Hf-free alloy ZX8; and (2) the synergy of Hf and Sn with the addition of Ti, Al, and Cr increased MACSi in EZ8, compared with the alloys EZ2, EZ5, and EZ6. However, compared with the Ge addition in equivalent Nb-18Si silicide-based alloys, where the synergy of 5 at.% Hf with 5 at.% Al, 5 at.% Cr, 5 at.% Ge, and 24 at.% Ti reduced MACSi in the alloy ZF9 (MACSi = 3.1 at.%, ZF9 = Nb-24Ti-18Si-5Al-5Cr-5Hf-5Ge, nominal [35]) but increased MACSi in the Hf-free alloy ZF6 (MACSi = 4.3 at.%, ZF6 = Nb-24Ti-18Si-5Al-5Cr-5Ge, nominal [35]), the synergy of Hf and Sn had a similar but weaker effect on MACSi (compare ZX8 and EZ8 (10 versus 7.7 at.%) with ZF6 and ZF9 (4.3 at.% versus 3.1 at.%)). To put it another way, MACSi is less of an issue when Al, Cr, Hf, Si, and Ti are simultaneously in synergy with Ge than with Sn, but given that all the aforementioned elements are key for the oxidation resistance of RM(Nb)ICs, and that the simultaneous presence of Ge and Sn in RM(Nb)ICs suppressed pest oxidation and scale spallation at high temperatures [41] (see Section 4.2.14), it is unlikely that MACSi-free and oxidation-resistant metallic UHTMs can be produced using cold-hearth processing.

Comparison of the alloys EZ2, EZ5, EZ6, and EZ8 with regard to the macrosegregation of Ti (MACTi) (Table 2) shows (a) that Al, when it was in synergy with Hf and Sn, decreased MACTi (compare the alloys EZ2 and EZ5); (b) that Cr, when it was in synergy with Hf and Sn, had a very strong effect on MACTi (alloys EZ2 and EZ6); and (c) that in the presence of Al, the effect of Cr, Hf, and Sn on MACTi was reduced (alloys EZ6 and EZ8). The macrosegregation of Cr (MACCr) that was observed in the alloy EZ6 (Table 2) was slightly reduced with the addition of Al in the alloy EZ8, which would suggest that the effect of the synergy of Ti, Hf, and Sn on MACCr was not annulled by the addition of Al.

### 4.2. Microstructures

#### 4.2.1. Suppression of Nb_3_Si

The alloying of Nb-18Si with 5 at.% Sn suppressed the formation of Nb_3_Si during solidification [39]. This effect of Sn was not “eliminated” when Sn was in synergy (a) with Ti [39], even though Ti stabilizes the Nb_3_Si [21,42,43,44,45]; (b) with Hf (alloy EZ1 [26]); or (c) with Ti and Hf (alloy EZ2), even though the synergy of the latter two elements in the absence of Sn furthered the formation of Nb_3_Si in the alloy Nb-24Ti-18Si-5Hf [25]. Thus, it was concluded that when Ti and/or Hf were in synergy with Sn, the latter element was still “in charge” of the suppression of Nb_3_Si.

The Nb_3_Si silicide was destabilized in RM(Nb)ICs (i) by Al (alloy KZ7, [21]); and (ii) by the synergy (a) of Hf and Al (alloy YG2, [25]), (b) of Sn and Al in the absence of Hf (alloy EZ7 [26]), and (c) of Sn with Al and Hf (alloy EZ4 [26], for the nominal compositions of alloys see the Table 6). Thus, in selecting the alloy EZ5 for this study, it was expected that the Nb_3_Si would be suppressed by the synergy of Hf and Sn with Al and Ti. This was confirmed by the experimental results. Furthermore, it has also been shown that in the alloy Nb-18Si-5Hf-5Cr (alloy YG1, [25]), the synergy of Hf and Cr destabilized the Nb_3_Si either via enhancing the transformation of the latter to Nb_ss_ and αNb_5_Si_3_ or by rendering the formation of Nb_3_Si sensitive to the cooling rate, so that it could not form during solidification with the high cooling rates prevailing in the bottom of the ingot. In other words, the synergy of Cr with Sn and Hf in EZ3-AC [26] further strengthened the destabilizing effect of Sn on the Nb_3_Si. This effect was not cancelled out by the addition of Ti in the alloy EZ6. Thus, it was expected that in EZ8-AC, the formation of Nb_3_Si would be suppressed by the synergy of Sn and Hf with Ti, Al, and Cr. This also was confirmed by the experimental results.

The absence of Nb_3_Si in the microstructures of the alloys EZ2, EZ5, EZ6, and EZ8 suggested that αNb_5_Si_3_ or γNb_5_Si_3_ could not be attributed to the phase transformations tP32 Nb_3_Si + βNb_5_Si_3_ → αNb_5_Si_3_ and tP32 Nb_3_Si → (Nb) + αNb_5_Si_3_ [38] or the solidification path L → tP32 Nb_3_Si → tP32 Nb_3_Si + Nb →(γNb_5_Si_3_ + Nb)_eutectic_ [24], where tP32 Nb_3_Si is the tetragonal Nb_3_Si and (Nb) the solid solution in the Nb-Si binary [38]. Furthermore, the data for the Ti-free alloys EZ1, EZ3, EZ4, and EZ7 in [26]; the Ti-containing alloys ZX3, ZX5, and ZX7 with 2 at.% Sn [22], and ZX4, ZX6, and ZX8 with 5 at.% Sn addition [23] (see Table 6 for nominal compositions); and the alloys of this work, taken together, warn that in RM(Nb)ICs and RM(Nb)ICs/RCCAs with Al, Cr, Hf, Si, Sn, and Ti addition, phase transformations that use the Nb_3_Si silicide to engineer the microstructure of the metallic UHTM cannot be used.

#### 4.2.2. Nb_ss_ + Nb_5_Si_3_ Eutectic

The suppression of Nb_3_Si in Nb-18Si-5Sn (alloy NV9, [39]) was accompanied with the stabilization of the Nb_ss_+Nb_5_Si_3_ eutectic, the formation of which was attributed to the addition of Sn given that such a eutectic does not exist in the equilibrium Nb-Si binary system [38]. The Nb_ss_+Nb_5_Si_3_ eutectic was not destabilized by the synergy of Sn with Hf in EZ1-AC [26].

The vol.% of the Nb_ss_ + Nb_5_Si_3_ eutectic was high in the alloy NV9, but with the addition of Ti in the alloy NV6, the vol.% of the eutectic was reduced [39]. Comparison of the microstructures of the alloys YG3-AC [25] and KZ3-AC (Nb-24Ti-18Si [21]) confirmed that the addition of Hf in the former stabilized the Nb_5_Si_3_, but the vol.% of Nb_5_Si_3_ was very low, as was the Nb_ss_+Nb_5_Si_3_ eutectic. The synergy of Sn and Hf with Ti in the alloy EZ2 reduced the vol.% of the eutectic compared with the alloy EZ1-AC [26]. Thus, it was concluded that when the alloying elements Ti, Hf, and Sn are in synergy in Nb-18Si based alloys, the vol.% of the Nb_ss_+Nb_5_Si_3_ eutectic is controlled by Sn, as the synergy of Ti and Hf favors Nb_3_Si selection and the Nb_ss_+Nb_3_Si eutectic (alloy YG3, [25]).

The effect of different alloying elements on the formation of the Nb_ss_+Nb_5_Si_3_ eutectic is summarized in Table 7. The addition of Al in the alloy YG2 [25] stabilized an Nb_ss_+Nb_5_Si_3_ eutectic in all parts of the as-cast ingot (Table 7). However, the addition of Al in the alloy EZ4 [26] made the formation of the Nb_ss_+Nb_5_Si_3_ eutectic susceptible to solidification conditions, as the eutectic was not formed in the bottom of the ingot (Table 7). Comparison of the alloys EZ1, EZ4, and YG2 would suggest that it is most likely the synergy of Al with Sn in the presence of Hf that makes the formation of the Nb_ss_+Nb_5_Si_3_ eutectic sensitive to cooling rate. This effect was accentuated in the alloy EZ5, in which the synergy of Al with Ti rendered the formation of the Nb_ss_+Nb_5_Si_3_ eutectic sensitive to cooling rate during solidification (Table 7). Comparison of EZ7-AC [26], in which a eutectic between Nb_3_Sn and Nb_5_Si_3_ was formed, with the alloys EZ4-AC [26] and EZ5-AC would suggest that it was Hf that destabilized the above eutectic, and that the addition of Ti in the alloy EZ5 enhanced the role of Hf in the formation of the Nb_ss_+Nb_5_Si_3_ eutectic. The <Si> = Si + Sn + Al content of the Nb_ss_+Nb_5_Si_3_ eutectic was in agreement with the <Si> of eutectics in RM(Nb)ICs and RM(Nb)ICs/RCCAs [13].

#### 4.2.3. Nb_ss_ + C14-NbCr_2_ Eutectic

In the alloy EZ3 [26], the synergy of Cr with Sn and Hf destabilized the Nb_ss_ + Nb_5_Si_3_ eutectic and instead resulted in the formation of a Nb_ss_ + C14-NbCr_2_ Laves phase eutectic (Table 7). In accordance with the results for the alloy EZ3 [26], no Nb_ss_ + Nb_5_Si_3_ eutectic was formed in EZ6-AC, in which the addition of Ti did not suppress the Nb_ss_ + C14-NbCr_2_ eutectic that had formed in the Ti-free alloy EZ3 [26]. Aluminum stabilizes the C14-NbCr_2_ Laves phase in the Nb-Cr-Al ternary system [46]. The addition of Al in Ti-containing RM(Nb)ICs did not suppress the formation of the C14-NbCr_2_ Laves phase (e.g., compare the alloy KZ4-AC with KZ5-AC in [21]), but the addition of Hf did (compare JN1-AC in [20] with KZ5-AC), and in both these alloys (i.e., KZ5 and JN1), the Nb_ss_+Nb_5_Si_3_ eutectic formed. The additions of Hf and Sn in EZ6 did not suppress the Laves phase (compare the alloy KZ4-AC [21] with EZ6-AC), in which the Nb_ss_ + NbCr_2_ eutectic formed. Similarly, the addition of Al in EZ8 did not suppress the Laves phase and the Nb_ss_ + NbCr_2_ eutectic, and shifted its composition closer to the eutectic in EZ3-AC [26] (Table 7). In the alloys EZ6-AC and EZ8-AC, the composition of the Nb_ss_+NbCr_2_ eutectic corresponded to that of a high-entropy eutectic (EZ6) or a complex concentrated eutectic (EZ8) [11,12]. It was concluded that in Ti-containing RM(Nb)ICs and RM(Nb)ICs/RCCAs, the synergy of Cr, Hf, and Sn promotes the stability of the C14-NbCr_2_ Laves phase and the formation of Nb_ss_+NbCr_2_ eutectic in the cast alloys, where the latter is a high-entropy eutectic or a complex concentrated eutectic that forms together with “conventional” phases (see [11]).

#### 4.2.4. The Nb_5_Si_3_ Silicide

Compared with the as-cast Nb-18Si-5Sn (alloy NV9 in [39]), alloying with Hf (alloy EZ1) or with Al (alloy EZ7) or with Hf and Cr (alloy EZ3, see Table 6 for the nominal compositions) shifted the composition of the Nb_5_Si_3_ away from the Nb-rich corner (also see Table 2 in [34]). This effect was reduced when Hf was in synergy with Ti (alloy EZ2) or with Al (alloy EZ4) or with both Ti and Cr (alloy EZ6) and was further reduced when Hf was in synergy with both Ti and Al (alloy EZ5) or with Ti and Al and Cr (alloy EZ8) (see Table 8). In EZ6-AC, the average concentrations of alloying elements in Nb_5_Si_3_ were similar to those of the same elements in the as-cast alloys EZ2, EZ3 [26], YG1, YG3 [25], and KZ4 [21]. The same was the case for Nb_5_Si_3_ in EZ6-HT2 compared with the aforementioned alloys. However, in EZ6-HT2, the range of Si concentrations in Nb_5_Si_3_ was wider and varied from 30.2 to 36.9 at%. In the Hf-rich Nb_5_Si_3_, the Ti and Hf concentrations were higher than those in the as-cast alloys EZ2, EZ3 [26], and YG1 [25]. After heat treatment, the Ti and Hf concentrations in the Hf-rich Nb_5_Si_3_ increased compared with EZ6-AC and were higher compared with the heat-treated alloys EZ2, EZ3 [26], and YG3 [25]. It is suggested that in the presence of Hf, the dominant elements controlling the partitioning of the different solutes between the Nb_5_Si_3_ phase and the melt were Ti and Al, with the latter being the most potent.

**Figure 12:**
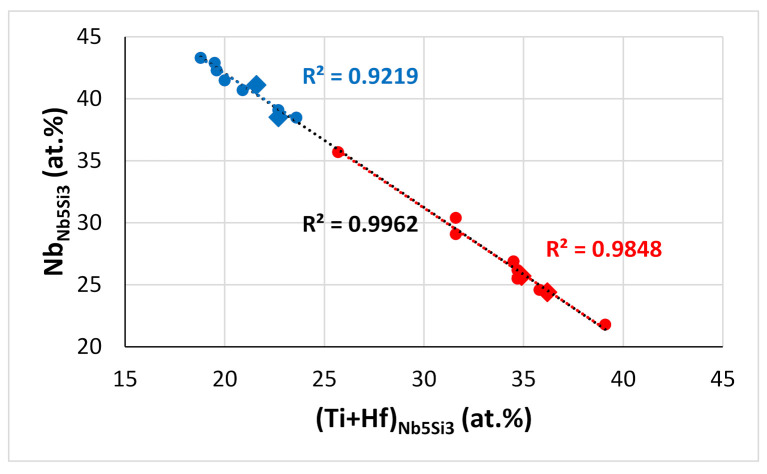
The Nb versus Ti + Hf map for the Nb_5_Si_3_ in the alloys of this work. Blue data for “normal” Nb_5_Si_3_, red data for Hf-rich Nb_5_Si_3_. For all data R^2^ = 0.9962. Diamonds for the RM(Nb)IC/RCCA alloy EZ8.

Note that complex concentrated silicide [11] co-existed with “conventional” phases in EZ2-AC (see 6 and 7 in the Figure 2a). Hexagonal Nb_5_Si_3_ was stable in all the alloys of this work after the heat treatment(s) (Table 3). All the data for the Nb_5_Si_3_ for all the alloys of this work gives Nb/(Ti + Hf) = 0.92, whereas this ratio is 0.97 and 0.93, respectively, for the “normal” Nb_5_Si_3_ and the Hf-rich Nb_5_Si_3_ (Figure 12). This is considered to indicate that the synergy of Sn with Hf and Ti in the absence of other TMs, RMs, and metalloid elements encourages the stability of the hexagonal γNb_5_Si_3_ in RM(Nb)ICs and RM(Nb)ICs/RCCAs.

**Figure 13:**
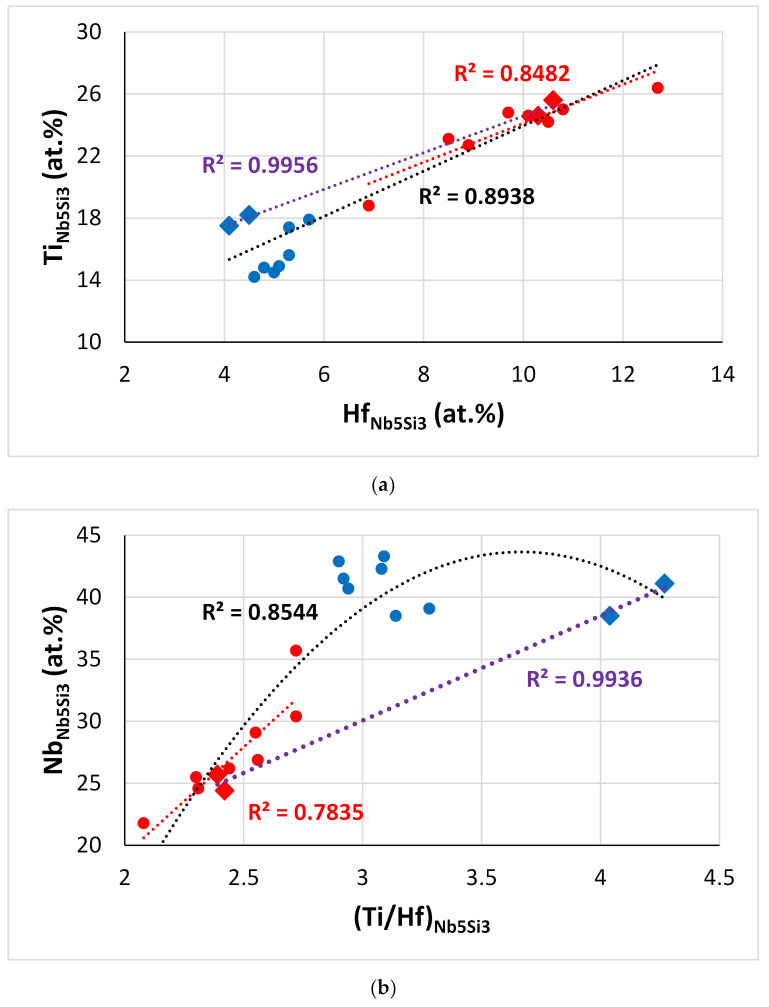
(**a**) The Ti versus Hf map and (**b**) the Nb versus Ti/Hf map for the Nb_5_Si_3_ in the alloys of this work. Blue data for “normal” Nb_5_Si_3_, red data for Hf-rich Nb_5_Si_3_. Diamonds for the RM(Nb)IC/RCCA alloy EZ8. In (**a**,**b**), the data for EZ8 have, respectively, R^2^ = 0.9956 and R^2^ = 0.9936. For all data in (**a**,**b**), the R^2^ value is 0.8938 and 0.8544, respectively.

The Ti versus Hf and the Nb versus Ti/Hf maps for the Nb_5_Si_3_ in the alloys of this work are shown in Figure 13. The Ti/Hf ratio was 1.46 when all the data for Nb_5_Si_3_ were taken into account (1.2 for the Nb_5_Si_3_ in EZ8), and it was 1.25 for the Hf-rich Nb_5_Si_3_. The maximum value of the Ti/Hf ratio was 3.67 for Nb = 43.65 at.%.

#### 4.2.5. The Nb_ss_ Solid Solution

Compared with EZ1-AC [26], in EZ2-AC the synergy of Ti with Hf and Sn did not affect the solubility of Si in the Nb_ss_ and Ti-rich Nb_ss_, but it increased the concentration of Sn in the Nb_ss_ by about 1.8 at.%. Thus, the Si+Sn concentration in Nb_ss_ and Ti-rich Nb_ss_ was about 10.3 at.% and 11.7 at.%, respectively, compared with 8.2 at.% in EZ1-AC [26]. In EZ2-HT, the Si concentration in Nb_ss_ was reduced to the same value as in EZ1-HT1 [26].

Compared with the alloy EZ7-AC [26], in which the Nb_ss_ was not stable, the data for the alloys EZ2, EZ4 [26], and EZ5 would suggest that the synergy of Hf with Ti (alloy EZ2), or with Al (alloy EZ4 [26]), or with Ti and Al (alloy EZ5) did not suppress the formation of Nb_ss_ during solidification that was controlled by the Si/Sn or Si/(Sn + Al) ratios (see Table 8). However, compared with the alloys EZ2-AC and EZ4-AC [26]—in which the Si+Sn and Si + Sn + Al concentrations in Nb_ss_ were, respectively, 10.3 at.% and 10.9 at.%—in EZ5-AC, the corresponding concentration was higher (about 15 at.%; see Table S2 in the Supplemental Data).

Compared with the alloy EZ2-AC, the Nb/Ti and Nb/(Ti + Hf) ratios in the Ti-rich Nb_ss_ in EZ6-AC were greater than 1, and in both alloys, the Hf and Sn content increased with the Ti concentration. However, for both solutes (i.e., Hf and Sn), the increase was more significant in EZ2-AC (no Cr present). The Si content of the Nb_ss_ was higher in EZ2-AC than in EZ6-AC, where it was the same as in EZ3-AC [26], KZ4-AC [21], and YG1-AC [25]. Thus, the data for the as-cast alloys YG1, YG3 [25], EZ2, EZ3 [26], and EZ6 would suggest that the synergy of Ti and Hf increased the Si concentration in the Nb_ss_. After heat treatment, there was no Ti-rich Nb_ss_, as was the case in the alloys EZ2, KZ4, and YG3. In EZ6-HT2, the Hf content of the Nb_ss_ decreased, as was the case in YG3-HT and EZ3-HT [26]. Furthermore, the Sn content of the Nb_ss_ decreased, as was the case in EZ3-HT. The concentration of Si in the Nb_ss_ was the same as in other RM(Nb)ICs but lower than that in EZ2-HT. Compared with the alloys EZ1 [26], EZ2, and EZ3 [26], the data for the alloy EZ6 would support the conclusion that the Nb_ss_ (and A15-Nb_3_X, see below) formation during solidification was controlled by the Si/Sn ratio (about 0.3) and the Si+Sn concentration (about 18 at.%), respectively, for the two phases (see Table 8).

The Nb_ss_ was not stable in the Al-, Hf-, and Ti-containing alloys EZ5 and EZ8 after heat treatment(s), even though it had formed in their cast microstructures (Table 3). Furthermore, in the Ti- and/or Hf-free alloys Nb-18Si-5Al-5Sn (EZ7) and Nb-18Si-5Al-5Hf-5Sn (EZ4) [26], the Nb_ss_ did not form during solidification and was not stable in EZ7 after the heat treatment (1500 °C/100 h), and also was not stable in EZ4 after the prolonged heat treatment (1500 °C/300 h). In all the aforementioned alloys (i.e., EZ4, EZ5, EZ7, EZ8), the concentration of Sn was 5 at.% (nominal). The Nb_ss_ was stable in the Hf-free but Al- and Ti-containing alloys ZX5 (Nb-24Ti-18Si-5Al-2Sn [22]) and ZX7 (Nb-24Ti-18Si-5Al-5Cr-2Sn [22]), and in the alloy ZX8 (Nb-24Ti-18Si-5Al-5Cr-5Sn [23]), but not in the alloy ZX6 (Nb-24Ti-18Si-5Al-5Sn [23]). Note that in the latter two alloys, the Sn concentration was 5 at.% (nominal), compared with 2 at.% in the former two alloys.

The data for the Ti-free alloys EZ4 and EZ7 suggested that the stability of Nb_ss_ was controlled by the synergy of 5 at.% Al with 5 at.% Sn. However, the Nb_ss_ was stable in the alloys EZ6, ZX5, ZX7, and ZX8 but not in the alloys ZX6, EZ5, and EZ8. The data would suggest (i) that Hf does indeed play a role in the stability of Nb_ss_; (ii) that the concentration of Sn in the alloy is important for the stability of Nb_ss_ in RM(Nb)ICs where Al, Sn, and Ti are in synergy; and (iii) that the effect of the synergy of Al, Hf, and Sn on the stability of Nb_ss_ in the absence/presence of Ti in the alloy is very strong and cannot be annulled with the addition of Cr.

Given that Al, Hf, and Sn are key alloying additions in RM(Nb)ICs and RM(Nb)ICs/RCCAs to obtain a balance of properties [3], alloy design must aim to optimize the concentrations of these elements to control (a) the vol.% of Nb_ss_ and (b) the stability of Nb_ss_ in the alloy, due to the fact that the Nb_ss_ is important for all three property goals, namely fracture toughness, creep, and oxidation. The alloy design methodology NICE [12] can take care of (a) and (b).

The lattice parameters of the Nb_ss_ in the alloys of this work were given in Table 5, where data is also included for comparable alloys. The lattice parameter of the Nb_ss_ was lower than that of pure Nb (3.303 Å), with the exception of EZ1-HT. Changes of the lattice parameter of Nb_ss_ were attributed to the size effect of solutes (1.429, 1.462, 1.153, 1.578, 1.62, 1.432, and 1.249 Å, respectively, for Nb, Ti, Si, Hf, Sn, Al, and Cr) and to changes in the chemical composition of the Nb_ss_ after heat treatment.

**Figure 14:**
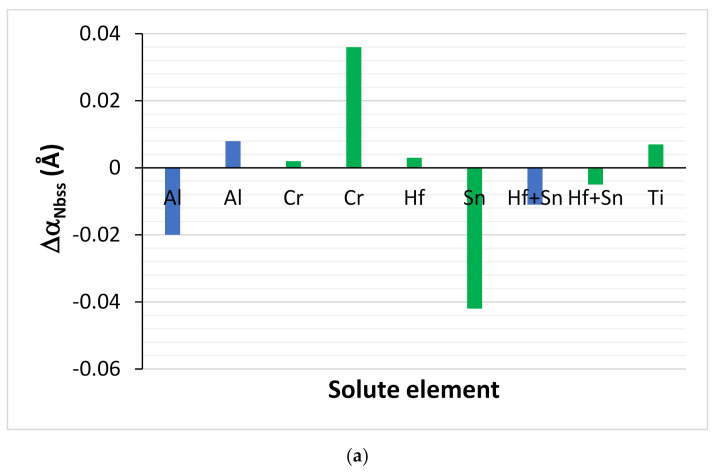

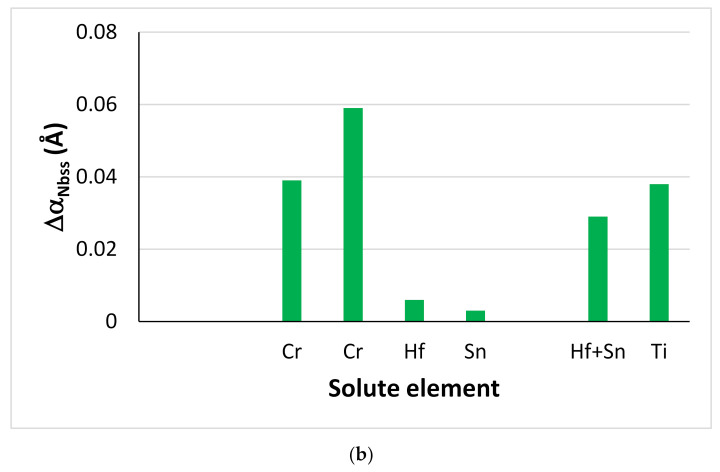
Change of lattice parameter of Nb_ss_ in (**a**) AC and (**b**) HT Nb-18Si-based RM(Nb)ICs. Note that Ti was present in all alloys with the exception of the alloy EZ1. For nominal alloy compositions and the lattice parameter data, see the Table 5. Green color is used for the Δα_Nbss_ of alloys for which there is data for the AC and HT conditions. In (**a**), from left to right, alloy comparisons are as follows: KZ3-AC vs. KZ7-AC (i.e., Δα_Nbss_ = α_KZ3_ − α_KZ7_), EZ2-AC vs. EZ5-AC, KZ3-AC vs. KZ4-AC, EZ2-AC vs. EZ6-AC, NV6-AC vs. EZ2-AC, KZ3-AC vs. NV6-AC, KZ7-AC vs. EZ5-AC synergy with Al, KZ4-AC vs. EZ6-AC synergy with Cr, and EZ1-AC vs. EZ2-AC. In (**b**), from left to right, alloy comparisons are as follows: KZ3-HT vs. KZ4-HT, EZ2-HT vs. EZ6-HT, NV6-HT vs. EZ2-HT, KZ3-HT vs. NV6-HT, KZ4-HT vs. EZ6-HT synergy with Cr, and EZ1-HT vs. EZ2-HT.

In cast alloys free of Hf and Sn addition, with the addition of Al or Cr, the lattice parameter (α_Nbss_) respectively decreased (Δα_Nbss_ < 0) and increased (Δα_Nbss_ > 0), whereas with the simultaneous addition of Al or Cr with Hf and Sn, the lattice parameter increased, most significantly in the case of Cr (Figure 14a). In heat-treated alloys with/without Hf and Sn in synergy with Cr, the lattice parameter increased (Figure 14b). With the addition of Hf, the Δα_Nbss_ was positive in as-cast and heat-treated alloys with Sn, whereas in Hf-free alloys, the addition of Sn resulted to Δα_Nbss_ < 0 in as-cast and Δα_Nbss_ > 0 in heat-treated alloys (Figure 14). The synergy of Hf and Sn simultaneously with Al or Cr decreased α_Nbss_ in cast alloys (Figure 14a) and increased α_Nbss_ in heat-treated alloys (Figure 14b). The synergy of Ti simultaneously with Hf and Sn in Al- and Cr-free alloys resulted in Δα_Nbss_ > 0, and the increase was more significant in the heat-treated condition.

**Figure 15:**
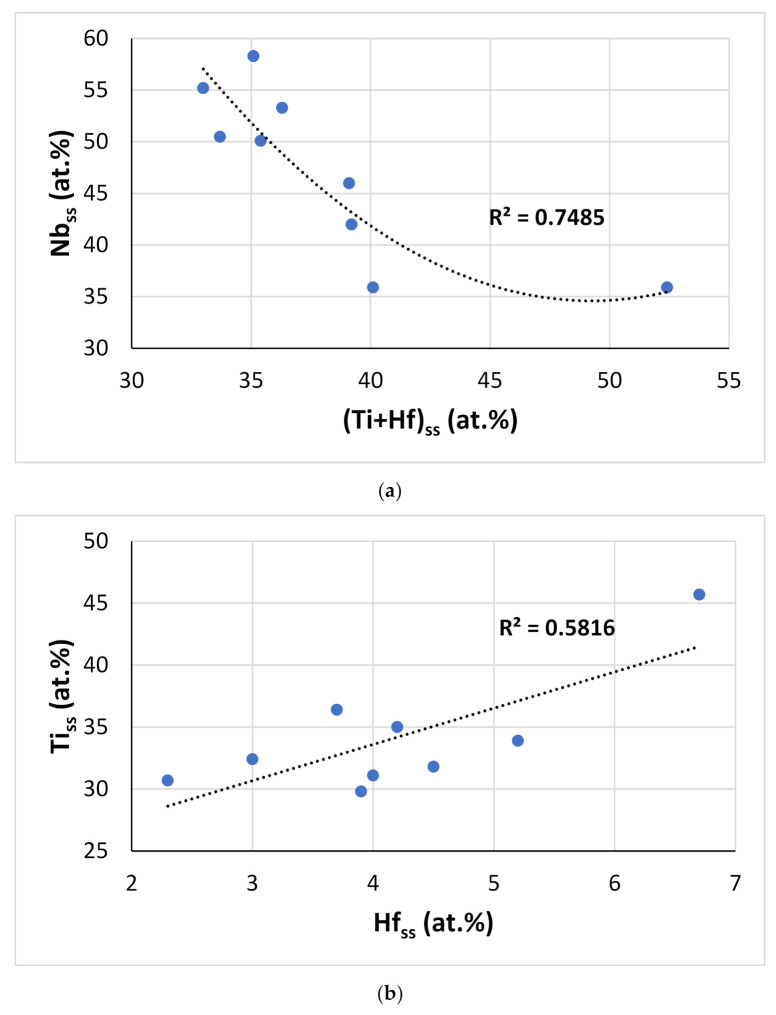

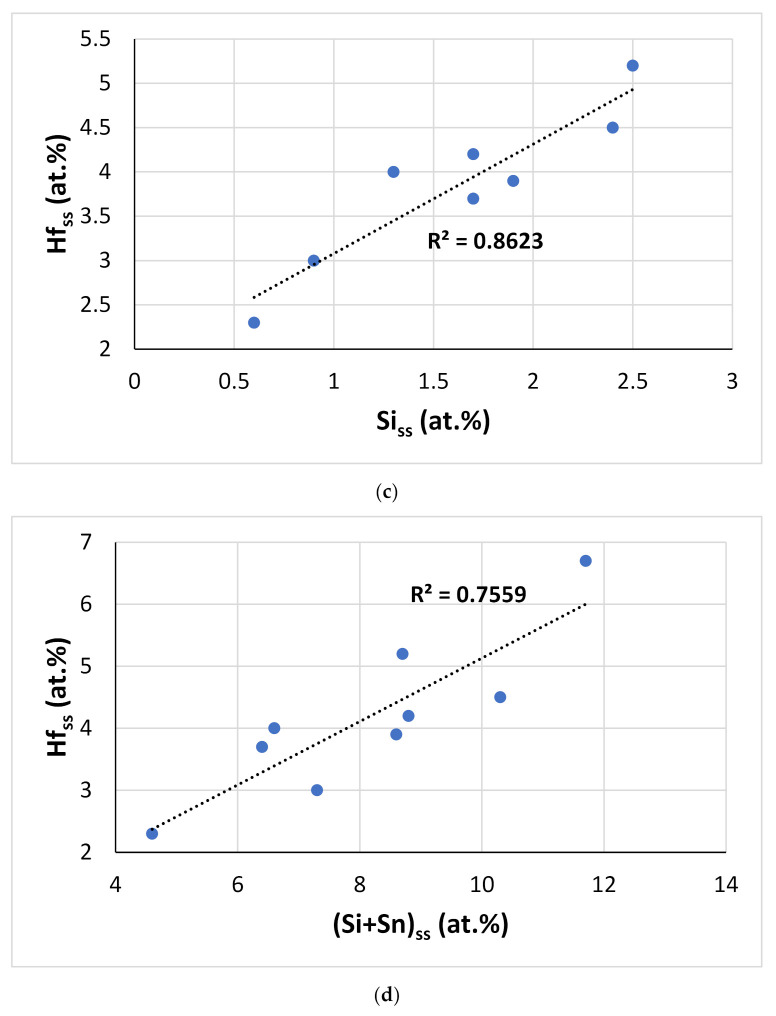
(**a**) Nb versus Ti + Hf, (**b**) Ti versus Hf, (**c**) Hf versus Si, and (**d**) Hf versus Si + Sn concentration in the Nb_ss_ in the alloys of this work.

It should be noted that the Nb_ss_ of the alloys of this work was Ti-rich (or in other words, the synergy of Hf with Sn and Ti resulted in Ti-rich Nb_ss_), with a minimum Ti concentration of about 31.4 at.%, for which the corresponding concentrations of Si, Sn, and Hf, respectively, were 0.8 at.%, 5.6 at.%, and 3.1 at.% (that is to say, the chemical composition of the Nb_ss_ with the minimum Ti content was 59.1Nb-31.4Ti-0.8Si-3.1Hf-5.6Sn, with Si + Sn and Ti + Hf sum, and Si/Sn, Ti/Hf, and Nb/(Ti + Hf) ratio, respectively, of 6.4 at.%, 34.5 at.%, 0.14, 10.1, and 1.71). The data also indicated Nb/(Ti + Hf) = 0.7 for minimum concentrations of Nb and Ti + Hf in the Nb_ss_ of, respectively, 34.6 at.% and 49.2 at.% (Figure 15a). Note that the Nb_ss_ associated with the lamellar microstructure in EZ2-AC had similar Ti + Hf sum and Nb/(Ti + Hf) ratio (respectively 45.6 at.% and 0.95). Increasing Ti concentration in the Nb_ss_ also increased the Hf concentration (Figure 15b), and with increasing Hf content in the Nb_ss_, the concentrations of Si and Sn increased by, respectively, 0.8 at.%/at.%Hf and 1.6 at.%/at.%Hf, as did the Si/Sn ratio and the Si+Sn sum, the latter by 1.96/at.%Hf (Figure 15c,d).

Considering that Hf, Si, Sn, and Ti are key elements for oxidation resistance and that refractory metals, in particular Mo and W, are key for strength and creep [1,3,11,12,33], the synergy of Hf, Sn, and Ti that promotes Ti rich Nb_ss_ (i) improves the oxidation resistance of the Nb_ss_ but (ii) reduces the strength and creep of the Nb_ss_ in alloys with RM addition, owing to the relationship between the concentration of Ti and RMs in the Nb_ss_ in RM(Nb)ICs and RM(Nb)ICs/RCCAs (e.g., see Figure S4 in the Supplemental Data in [47], Figure 12 in [48], and Figure 12 in [49]). Thus, a challenge for alloy design is to “balance” these effects. This is achievable with the alloy design methodology NICE [3,11,12].

#### 4.2.6. The A15-Nb_3_X Compound

The addition of Sn promoted the formation of Nb_3_Sn in the alloys Nb-18Si-5Sn (NV9) and Nb-24Ti-18Si-5Sn (NV6) [39], where the Nb_3_Sn was present in all parts of the ingot of each alloy. However, the addition of Hf in the alloys EZ1 [26] and EZ2, despite the fact that it did not completely destabilize the A15-Nb_3_X, essentially rendered its formation sensitive to cooling rate, probably due to the fact that Hf affected the Si+Sn and Si/Sn values that control its formation. On the other hand, Al addition not only promoted the formation of the A15-Nb_3_X (EZ1 vs. EZ7 [26]) but actually reversed the effect that Hf had on it, by stabilizing the formation of the A15-Nb_3_X to most parts of the as-cast ingots and also by increasing the vol.% of this phase (EZ1 vs. EZ4 [26], EZ2 vs. EZ5), indicating that its effect on the partitioning of Sn (and thus the Si + Sn + Al and Si/(Sn + Al) values) between the solid phases and the melt was stronger than that of Hf. As a matter of fact, the Si + Sn + Al concentration in the Al-containing alloys EZ4, EZ5, and EZ7 was higher by about 2 at.% (see Table 8 and Table 9). A similar effect to that of Al was seen with the addition of Cr in the alloys Nb-18Si-5Cr-5Hf-5Sn (EZ3 vs. EZ1 [26]) and Nb-24Ti-18Si-5Cr-5Hf-5Sn (EZ6 vs. EZ2), the presence of which resulted in the stabilization of the A15-Nb_3_X in all areas of the as-cast ingots (Table 9). Thus, it was expected that when Al and Cr were present simultaneously, as in the case of the alloy EZ8, the A15-Nb_3_X would be stabilized in all parts of the ingot. This was confirmed by the experimental results for EZ8-AC. Furthermore, the increased vol. % of A15-Nb_3_X in EZ8-AC was in agreement with the results for the alloys EZ1 to EZ7 [26].

It should be noted that for the alloys of this work, the minimum concentration of Ti in the A15-Nb_3_X (about 24.9 at.%) corresponded to Si = 4.9 at.%, Sn = 11.26 at.%, that is, the chemical composition of the A15-Nb_3_X with the minimum Ti content was 58.9Nb-24.9Ti-4.9Si-11.3Sn, with Si+Sn sum and Si/Sn ratio of, respectively, 15.2 at.% and 0.43, in good agreement with the Si, Sn, and Ti concentrations in the cast alloys EZ5, EZ6, and EZ8.

**Figure 16:**
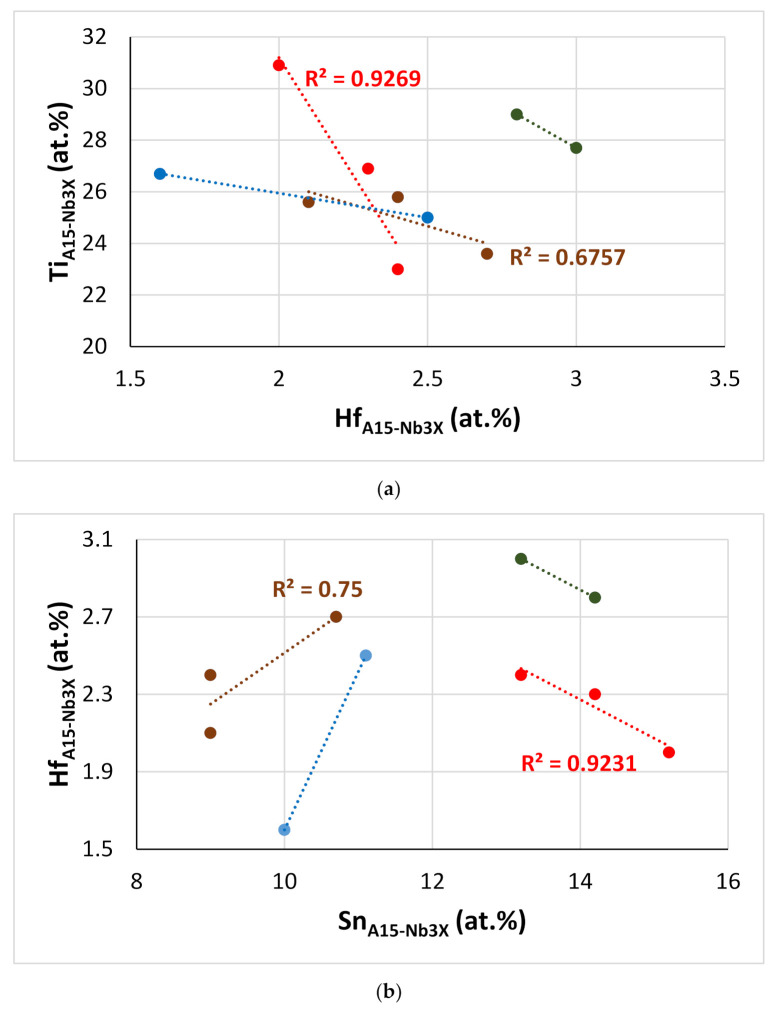

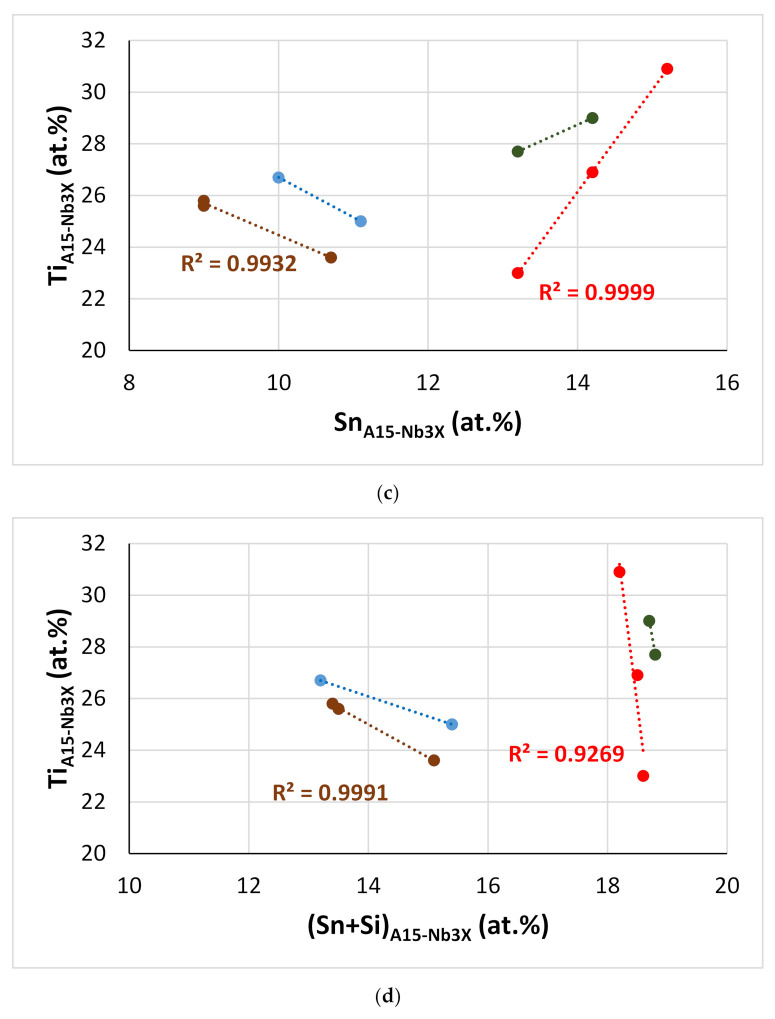

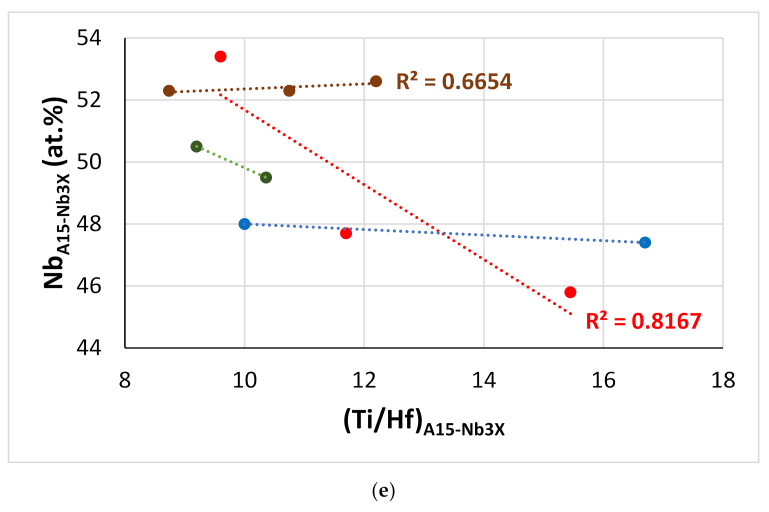
Trends in solute addition concentrations in A15-Nb_3_X in the alloys of this work: (**a**) Ti vs. Hf, (**b**) Hf vs. Sn, (**c**) Ti vs. Sn, (**d**) Ti vs. Si+Sn, and (**e**) Nb vs. Ti/Hf. Colors are as follows: EZ2 green, EZ5 brown, EZ6 red, EZ8 blue.

Figure 16 shows correlations between Hf, Si, Sn, and Ti concentrations in A15-Nb_3_X compounds in the alloys of this work. Note the similar trends exhibited by the alloys EZ2 and EZ6 (green and red data) and the alloys EZ5 and EZ8 (brown and blue data), and remember the addition of Cr in EZ6, and that Al was present in both EZ5 and EZ8. In all the alloys, as the Hf content in A15-Nb_3_X increased, that of Ti decreased (Figure 16a), but the trends of Hf vs. Sn and Ti vs. Sn were opposite for the alloys EZ5 and EZ8 (as Hf increased, the Sn content increased too (Figure 16b), and as Ti decreased, the Sn content increased (Figure 16c)) and the alloys EZ2 and EZ6 (Figure 16b,c). Note also that the Sn and Si+Sn content of the A15-Nb_3_X in the latter alloys was higher than in the former (Figure 16b–d), but with the Si+Sn concentration in a very narrow range (18.2 to 18.8 at.%, Figure 16d). Furthermore, the Nb concentration of the A15-Nb_3_X decreased significantly with increasing Ti/Hf ratio in the Al-free alloys (Figure 16e).

#### 4.2.7. C14-NbCr_2_ Laves Phase

**Figure 17:**
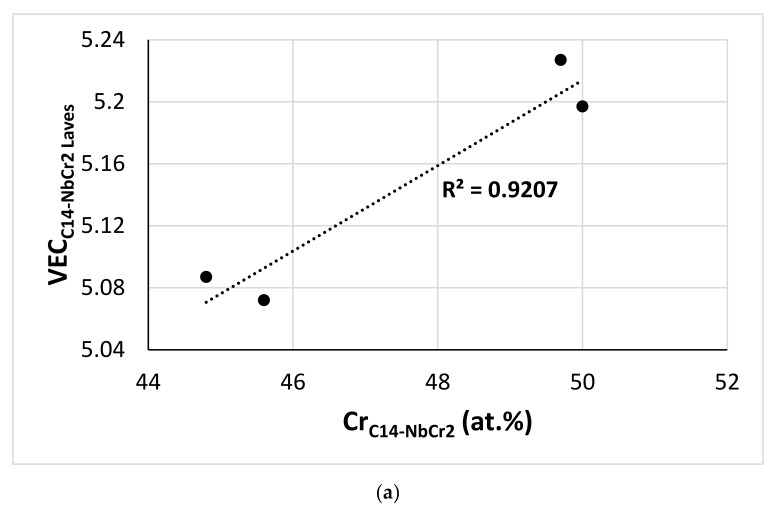

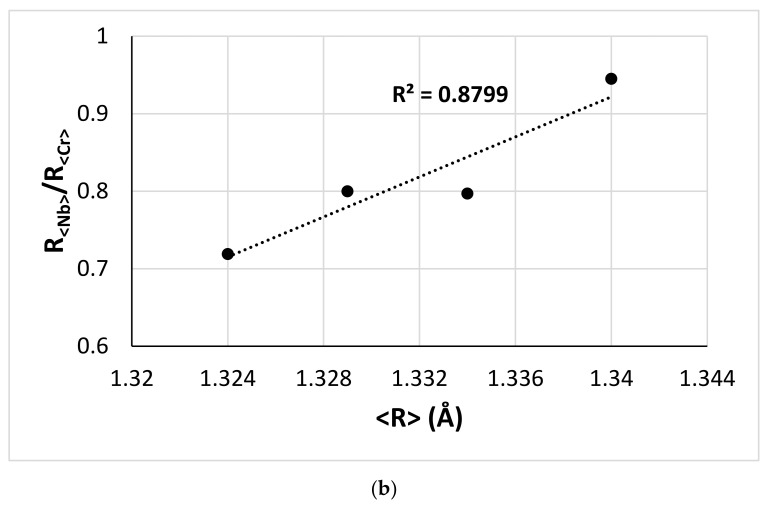
Data for the C14-NbCr_2_ Laves phase in the alloys of this work: (**a**) VEC versus Cr content and (**b**) R_<Nb>_/R_<Cr>_ versus <R> = R_<Nb>_ + R_<Cr>_, where R_<Nb>_ = ∑_i_^n^C_i_(r_<Nb>_)_i_, where C_i_ and (r_<Nb>_)_i_ are, respectively, the concentration (at.%) and atomic radius of Nb and element i substituting Nb in the Laves phase; and R_<Cr>_ = ∑_i_^n^C_i_(r_<Cr>_)_i_, where C_i_ and (r_<Cr>_)_i_ are, respectively, the concentration (at.%) and atomic radius of Cr and element i substituting Cr in the Laves phase.

The <Cr> = Cr + Al + Si + Sn content of the C14-NbCr_2_ Laves phase in the cast and heat-treated alloys of this work was in agreement with other work on RM(Nb)ICs [13,50]. Figure 17 shows the correlation between VEC and Cr content of the C14-NbCr_2_ Laves phase and the average atomic size <R>, with the ratio of the average atomic sizes of elements that substitute Nb or Cr in the Laves. The two correlations are in agreement with [13].

#### 4.2.8. Vol.% of Phases

**Figure 18:**
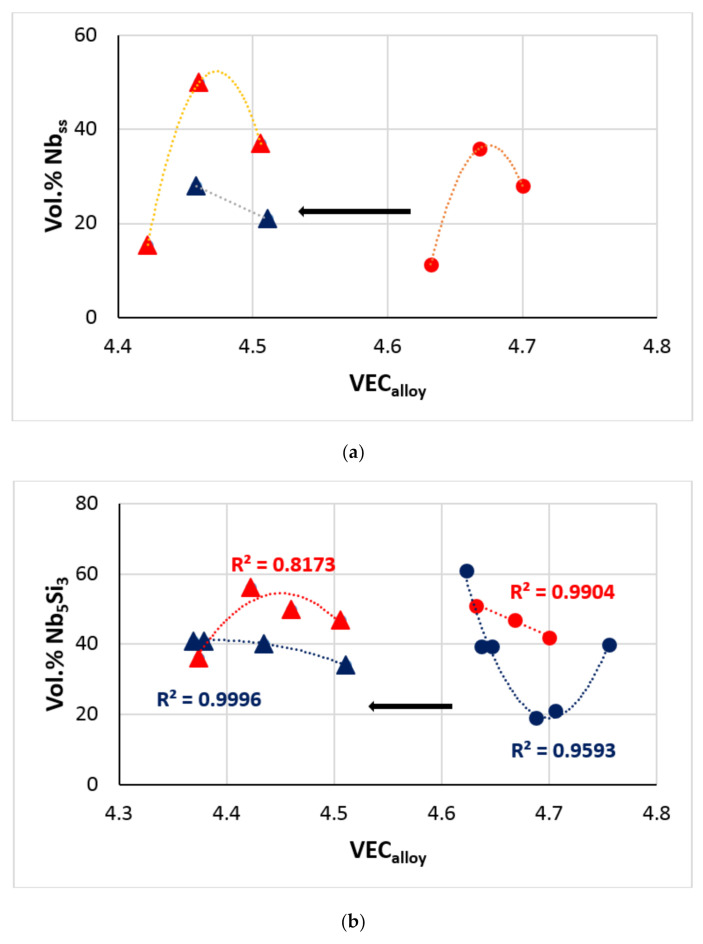

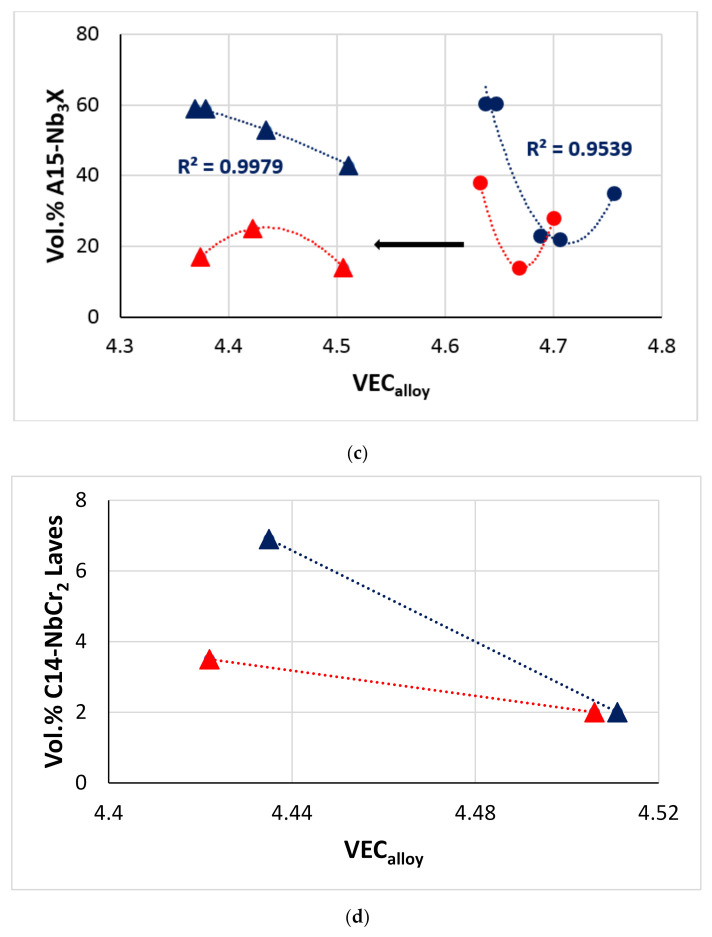
Correlation of the volume fraction of the phases in RM(Nb)ICs with/without Ti addition with the parameter VEC of the alloys that were studied in [26] and this work: (**a**) Nb_ss_, (**b**) Nb_5_Si_3_, (**c**) A15-Nb_3_X, and (**d**) C14-NbCr_2_ Laves phase. Colors and symbols as follows: red for as-cast alloys, blue for HT alloys, circles for Ti-free alloys, and triangles for Ti-containing alloys. In (**a**–**c**), the black arrow indicates the effect of Ti addition. In (**a**,**d**), the dotted lines are given to “guide the eye”.

Correlations of the vol.% of phases in RM(Nb)ICs with/without Ti addition with VEC_alloy_ are shown in the Figure 18. Notice (i) that Al, Cr, Hf, Si, Sn, and Ti are key alloying additions in RM(Nb)ICs for improving oxidation and for controlling strength, fracture toughness, and creep (e.g., [32,33,51,52,53,54]); (ii) that the vol.% of Nb_ss_ and Nb_5_Si_3_ is key for fracture toughness, creep, and oxidation resistance, of which the former and the latter two have a tendency to increase and decrease with increasing vol.% Nb_ss_, respectively, and to decrease and increase with increasing vol.% of Nb_5_Si_3_ (e.g., [12,32,55]); (iii) that the C14-NbCr_2_ Laves phase can improve the oxidation of RM(Nb)ICs but at the expense of their fracture toughness (e.g., [32,56]); (iv) that the A15-Nb_3_X is key to improving the oxidation of RM(Nb)ICs in the range of temperatures of pest oxidation and at high temperatures [22,23,27]; and (v) that according to NICE, in order to meet the oxidation or creep goal, VEC_alloy_ should decrease and increase, respectively [3,12]. In addition, note that the black arrow in the Figure 18a–c shows that the Ti effect is consistent with the improvement of oxidation resistance of RM(Nb)ICs owing to the corresponding reduction of VEC_alloy_ [1,3,11,12,50].

Notice (a) that the vol.% of Nb_ss_, Nb_5_Si_3_, A15-Nb_3_X, and C14-NbCr_2_ Laves decreased and increased after heat treatment for the former two and the latter two phases, respectively (Figure 18a–d); (b) that similar trends were exhibited by the data for Nb_5_Si_3_ and A15-Nb_3_X in the alloys with/without Ti addition; (c) that up to about (i) 30% Nb_ss_ could be stable without Al and Cr addition in the alloy EZ2 (Figure 18a), and (ii) 40% Nb_5_Si_3_ and 7% C14-NbCr_2_ Laves could be stable in the alloy EZ8 with simultaneous Al and Cr addition (Figure 18b,d); (d) that the vol% of A15-Nb_3_X significantly increased after heat treatment in Cr-free alloys with Al addition and with/without Ti addition (Figure 18c), and that the aforementioned volume fractions correlate with low VEC_alloy_ values, which is an essential requirement for improved oxidation resistance according to NICE [1,3,12]. In other words, the data would suggest that with Ti, Hf, Si, Sn, and Al or Cr additions, the oxidation of RM(Nb)ICs and RM(Nb)ICs/RCCAs (note that the alloy EZ8 is also a RM(Nb)IC/RCCA alloy) can be improved owing to the decrease of VEC_alloy_. It should be noted (i) that improved oxidation resistance in the pest temperature range was confirmed for all the heat-treated alloys of this work, which did not pest and did not suffer from scale spallation; and (ii) that in oxidation at 1200 °C of the alloys with Al and/or Cr addition, their scales spalled off, similarly to other Sn-containing RM(Nb)ICs [22,23] (oxidation data is not included in this paper).

#### 4.2.9. Partitioning of Solutes and Solidification of the Alloys

The synergy of Hf and Sn in RM(Nb)ICs with/without Ti addition not only made the characterization of the microstructures difficult (this work and [26]) but also promoted (i) different types of Nb_5_Si_3_ in the microstructure (meaning tetragonal α or βNb_5_Si_3_ or hexagonal γNb_5_Si_3_, Table 3) and/or (ii) microstructures that arise from phase transformations that involve the two key phases, namely Nb_5_Si_3_ and Nb_ss_. The melt solidified following different solidification paths in different parts of the ingot.

Three different microstructures were observed in EZ5-AC owing to the sensitivity of the solidification of this alloy to the partitioning of solutes in different parts of the solidifying melt. The βNb_5_Si_3_ was the primary phase. The microstructure in the bulk of EZ5-AC consisted of primary βNb_5_Si_3_ surrounded by Nb_ss_+Nb_5_Si_3_ eutectic and the A15-Nb_3_X. As the primary βNb_5_Si_3_ formed, the melt became poorer in Si and richer in Ti, and when the melt concentration reached Si + Al + Sn of about 23 at.%, the eutectic formed. As the melt became poorer in Si, the Si + Al + Sn concentration reached about 20.6 at.%, and the A15-Nb_3_X formed. It is suggested that the solidification path in the bulk of EZ5-AC was L → L + βNb_5_Si_3_ → L + βNb_5_Si_3_ + γNb_5_Si_3_ + [Nb_ss_ + Nb_5_Si_3_]_eutectic_ → βNb_5_Si_3_ + γNb_5_Si_3_ + [Nb_ss_ + Nb_5_Si_3_]_eutectic_ + A15-Nb_3_X + αNb_5_Si_3_. The Si+Al+Sn concentration of the Nb_5_Si_3_ and the Hf-rich Nb_5_Si_3_ was about 37.6 at% and 38.5 at.%, respectively. These values were very close to the ones observed in the bulk of the alloy EZ4-AC [26]. The Ti addition in EZ5 had a significant effect on the partitioning of Hf, and the concentrations of Ti and Hf increased by, respectively, about 8.1 at.% and 6.3 at.% in the Hf-rich Nb_5_Si_3_, leading to Nb/(Ti + Hf) about 0.7, which indicated hexagonal γNb_5_Si_3_ [24]. The Ti addition in EZ5 also affected the composition of the Nb_ss_, in which the average Si + Al + Sn concentration was about 15.9 at.%, that is, about 5 at.% higher than in the Nb_ss_ in the alloy EZ4-AC [26], and Si/(Al + Sn) about 0.2 (the same as in the alloy EZ4-AC, see Table 8). The Sn content in A15-Nb_3_X was also affected by the addition of Ti, and in the bulk of EZ5-AC, it was about 4 at.% higher than in the alloy EZ4-AC.

In the top of EZ5-AC, the microstructure consisted of primary Nb_5_Si_3_ surrounded by the Nb_ss_. There were Hf-rich areas in the βNb_5_Si_3_, with the core exhibiting a darker contrast owing to the lower Hf concentration. As the primary βNb_5_Si_3_ formed, the melt became poorer in Si + Al + Sn, and when the latter concentration reached about 16 at.% and Si/(Al + Sn) about 0.2, the Nb_ss_ formed. It is suggested that the solidification path in the top of EZ5-AC was L → L + βNb_5_Si_3_ → βNb_5_Si_3_ + Nb_ss_ → βNb_5_Si_3_ + Nb_ss_ + αNb_5_Si_3_. In the bottom of EZ5-AC, the microstructure consisted of the Nb_5_Si_3_, Nb_ss_, and A15-Nb_3_X phases. The βNb_5_Si_3_ was the primary phase. It is suggested that the solidification path in this part of the ingot was L → L + βNb_5_Si_3_ + γNb_5_Si_3_ → L + βNb_5_Si_3_ + γNb_5_Si_3_ + A15-Nb_3_X + Nb_ss_ → βNb_5_Si_3_ + γNb_5_Si_3_ + αNb_5_Si_3_ + A15-Nb_3_X + Nb_ss_.

Two different microstructures were observed in EZ8-AC owing to the sensitivity of the solidification of this alloy to the partitioning of the solutes in the different areas of the ingot. The alloy EZ8 was hypereutectic in the top and bulk of the ingot and solidified with the βNb_5_Si_3_ as its primary phase. As the primary βNb_5_Si_3_ formed, the melt became richer in Ti and Hf, and Hf-rich Nb_5_Si_3_ formed. The solubility of Cr and Sn in particular, and of Al in the Hf-rich Nb_5_Si_3_, was small (Table S4 in the Supplemental Data). Thus, the melt surrounding the βNb_5_Si_3_ became richer in Al, Cr, and Sn and leaner in Si and Hf. When the composition of the melt reached Si + Al + Sn about 20.9 at.%, the A15-Nb_3_X formed. The Hf and Si were rejected into the melt, which became rich in these elements. When the Si/(Sn+Al) ratio in the melt reached about 0.2, the Nb_ss_ formed. Then, the melt became richer in Si, and when its composition reached Cr + Si + Al + Sn about 49 at.%, a eutectic between the NbCr_2_ Laves phase and the Nb_ss_ grew. The aforementioned eutectic formed in between these phases as the solutes partitioned between the solidifying intermetallics and the solid solution. The presence of Nb_ss_, αNb_5_Si_3_, γNb_5_Si_3_, and Hf-rich Nb_5_Si_3_ in EZ8-AC is in agreement with [16]. The C14-NbCr_2_ Laves phase was not observed in [16] owing to the low Cr concentration in the Nb-25Ti-16Si-8Hf-2Al-2Cr-xSn (x = 2 to 8 at.%) alloys. The A15-Nb_3_X was not observed in the alloy with x = 5 at.% Sn, but was stable after the heat treatment [16].

According to the XRD data (Figure S4 in the Supplemental Data), both αNb_5_Si_3_ and βNb_5_Si_3_ were present in EZ8-AC, which suggests that during the cooling down of the ingot, some βNb_5_Si_3_ transformed to αNb_5_Si_3_. In addition, during the cooling down of the EZ8-AC ingot, a lamellar microstructure formed between the Hf-rich Nb_5_Si_3_ silicide and the A15-Nb_3_X (see A in Figure 10). It is suggested that the solidification of EZ8 was L → L + βNb_5_Si_3_ → L + βNb_5_Si_3_ + γNb_5_Si_3_ → L + βNb_5_Si_3_ + γNb_5_Si_3_ + A15-Nb_3_X → L + βNb_5_Si_3_ + γNb_5_Si_3_ + A15-Nb_3_X + Nb_ss_ → L + βNb_5_Si_3_ + γNb_5_Si_3_ + A15-Nb_3_X + Nb_ss_ + [NbCr_2_ + Nb_ss_]_eutectic_ → βNb_5_Si_3_ + γNb_5_Si_3_ + A15-Nb_3_X + Nb_ss_ + [NbCr_2_ + Nb_ss_]_eutectic_ + αNb_5_Si_3_.

In EZ2-AC, the primary phase was the βNb_5_Si_3_, and as the liquid became poorer in Si, the Si+Sn concentration reached about 22 at.%, and the eutectic formed around the primary βNb_5_Si_3_. It is suggested that the solidification path of EZ2-AC in the top and bulk of the ingot (where the A15-Nb_3_X was not observed) was L → L + βNb_5_Si_3_ → L + βNb_5_Si_3_ + [Nb_ss_ + Nb_5_Si_3_]_eutectic_ → βNb_5_Si_3_ + [Nb_ss_ + Nb_5_Si_3_]_eutectic_ + αNb_5_Si_3_. In the bottom of the ingot the solidification path was L → L + βNb_5_Si_3_ → L + βNb_5_Si_3_ + A15-Nb_3_X → Nb_5_Si_3_ + A15-Nb_3_X + [Nb_ss_ + Nb_5_Si_3_]_eutectic_.

The EZ6-AC solidification started with the βNb_5_Si_3_ silicide, which was subsequently surrounded by Ti-rich Nb_ss_. The Hf-rich Nb_5_Si_3_ was in the parts with the higher Ti concentration. The A15-Nb_3_X was formed next to the Nb_ss_, while the C14-NbCr_2_ Laves phase grew in between the A15-Nb_3_X and the Nb_ss_ or near the Nb_ss_. It is suggested that the solidification path of EZ6-AC was L → L + βNb_5_Si_3_ → L + βNb_5_Si_3_ + Nb_ss_ → L + βNb_5_Si_3_ + γNb_5_Si_3_ + Nb_ss_ + A15-Nb_3_X → L + βNb_5_Si_3_ + γNb_5_Si_3_ + Nb_ss_ + A15-Nb_3_X + [NbCr_2_ + Nb_ss_]_eutectic_ → βNb_5_Si_3_ + αNb_5_Si_3_ + γNb_5_Si_3_ + A15-Nb_3_X + Nb_ss_ + [NbCr_2_ + Nb_ss_]_eutectic_.

Owing to the partitioning of solutes, a lamellar microstructure was formed in the bulk of EZ2-AC and microstructure A in EZ6-HT2. The chemical composition of the former corresponded to high-entropy or complex concentrated lamellar microstructure (see Section 3.1) and coexisted with “conventional” phases [11]. The chemical composition of microstructure A and the bright contrast phase (BCP, see Section 3.8) also corresponded to complex concentrated microstructure [11]. These microstructures are considered further in the next two sections.

#### 4.2.10. Eutectic and Lamellar Microstructures in EZ2-AC

The case of the Nb_ss_ + Nb_5_Si_3_ eutectic and the lamellar microstructures observed only in the bulk of EZ2-AC (Figure 1 and Figure 2) requires attention. The chemical composition of said phases will be taken into account. In EZ2-AC, there was Ti-rich Nb_ss_, similarly to the alloy YG3-AC (see Table 6 for nominal composition), but in EZ2-AC, the concentration of Ti in the Nb_ss_ was higher than that of Nb (i.e., the Ti-rich Nb_ss_ in EZ2 had Nb/Ti < 1), and the concentrations of Sn and Hf in the Nb_ss_ increased with the Ti concentration. It should be noted that Ti-rich Nb_ss_ was not observed in the alloy NV6-AC (Nb-24Ti-18Si-5Sn [39]), which would suggest that in the presence of Sn, the synergy of Ti and Hf had a strong effect on the partitioning of Ti to the Nb_ss_.

In EZ2-AC, the Ti and Hf concentrations in the Nb_5_Si_3_ were similar to those in YG3-AC [25]. In the latter alloy, the Hf-rich Nb_5_Si_3_ corresponded to hexagonal γNb_5_Si_3_ according to its Nb/(Ti + Hf) ratio, whereas in EZ2-HT, the Hf- and Ti-rich tetragonal Nb_5_Si_3_ that was already present in EZ2-AC became richer in both Ti and Hf after the heat treatment, and according to its Nb/(Ti + Hf) ratio (0.7), it was hexagonal γNb_5_Si_3_ [24]. In EZ2-AC, the average Si+Sn concentration in Nb_5_Si_3_ was 38.0 at% and did not change significantly in EZ2-HT (38.5 at.%), similarly with EZ1-HT [26]. The Ti addition in EZ2 did not change the solubility of Sn in the silicide, which was similar to that in EZ1-AC, but increased the Hf concentration. In EZ2-AC, in the Hf-rich Nb_5_Si_3_, the Ti concentration increased by about 4.6 at.% compared with the “normal” Nb_5_Si_3_, but the Si+Sn, Si, and Sn concentrations did not change.

It was suggested [25] that the Hf-rich Nb_5_Si_3_ that formed in YG3-HT was the product of the eutectoid transformation tP32 Nb_3_Si → Nb_ss_ + Hf-rich (hP16) Nb_5_Si_3_ and that the synergy of Ti and Hf in YG3 led to the replacement of the eutectoid transformation tP32 Nb_3_Si → Nb_ss_ + (tI32) αNb_5_Si_3_ with the alternative eutectoid phase transformation given above, in which the Nb_5_Si_3_ had the hexagonal (hP16) structure instead of the tetragonal (tI32) one [24]. As the Nb_3_Si was not formed in EZ2, the above phase transformations cannot account for the presence of γNb_5_Si_3_ and αNb_5_Si_3_.

In EZ2-AC, there was tetragonal βNb_5_Si_3_ and αNb_5_Si_3_ according to the XRD data (Figure S1 in the Supplemental Data). The synergy of Ti and Sn in the alloy NV6 (Nb-24Ti-18Si-5Sn) enhanced the transformation βNb_5_Si_3_ → αNb_5_Si_3_ [39]. The formation of αNb_5_Si_3_ in EZ2-AC was attributed to the presence of both Ti and Sn in the alloy. Furthermore, the addition of Ti in EZ2 decreased the liquidus of the alloy, and thus, as the homologous temperature increased, the solute diffusivities increased during solid-state cooling. In other words, in EZ2-AC, the βNb_5_Si_3_ was the primary phase and the βNb_5_Si_3_ → αNb_5_Si_3_ phase transformation had started during the cooling of the ingot.

Figure 2 shows (a) a lamellar microstructure that grew into a blocky faceted Nb_5_Si_3_ grain (see microstructure on the left of number 17 in Figure 2b, and note that outer parts of some Nb_5_Si_3_ were richer in Hf with lower Ti/Hf ratio compared with the inner darker contrast parts, Figure 1a,b), with a lamellar microstructure (numbers 1, 2, 3, and 4 in Figure 2a) that connected with (was adjacent to) Nb_5_Si_3_ grains (numbers 6 and 7 in Figure 2a).

The lamellar microstructure (i) was significantly richer in Ti and Hf than the Nb_ss_+Nb_5_Si_3_ eutectic in EZ2-AC and (ii) had higher and lower Ti + Hf sum and Nb/(Ti + Hf) ratio, respectively, than the eutectic. Furthermore, the Nb_5_Si_3_ associated with the lamellar microstructure (e.g., numbers 6 and 7 in Figure 2a) was (iii) richer in Ti and Hf and had tetragonal structure like the tetragonal, and poorer in Ti and Hf, “normal” Nb_5_Si_3_. In addition, the lamellar microstructure and the Nb_5_Si_3_ associated with it had chemical composition consistent with high-entropy or complex concentrated phases [11]. Moreover, the Nb_ss_ near the lamellar microstructure (e.g., numbers 11, 12, 15, 17, and 18 in Figure 2) was (iv) richer in Ti and Hf compared with the Nb_ss_ away from the lamellar microstructure, and (v) had a Nb/(Ti + Hf) ratio close to that corresponding to the minimum Ti + Hf and Nb concentrations in the Nb_ss_ (Figure 15a). Chemical analysis of the individual phases in the lamellar microstructure was not possible.

**Figure 19:**
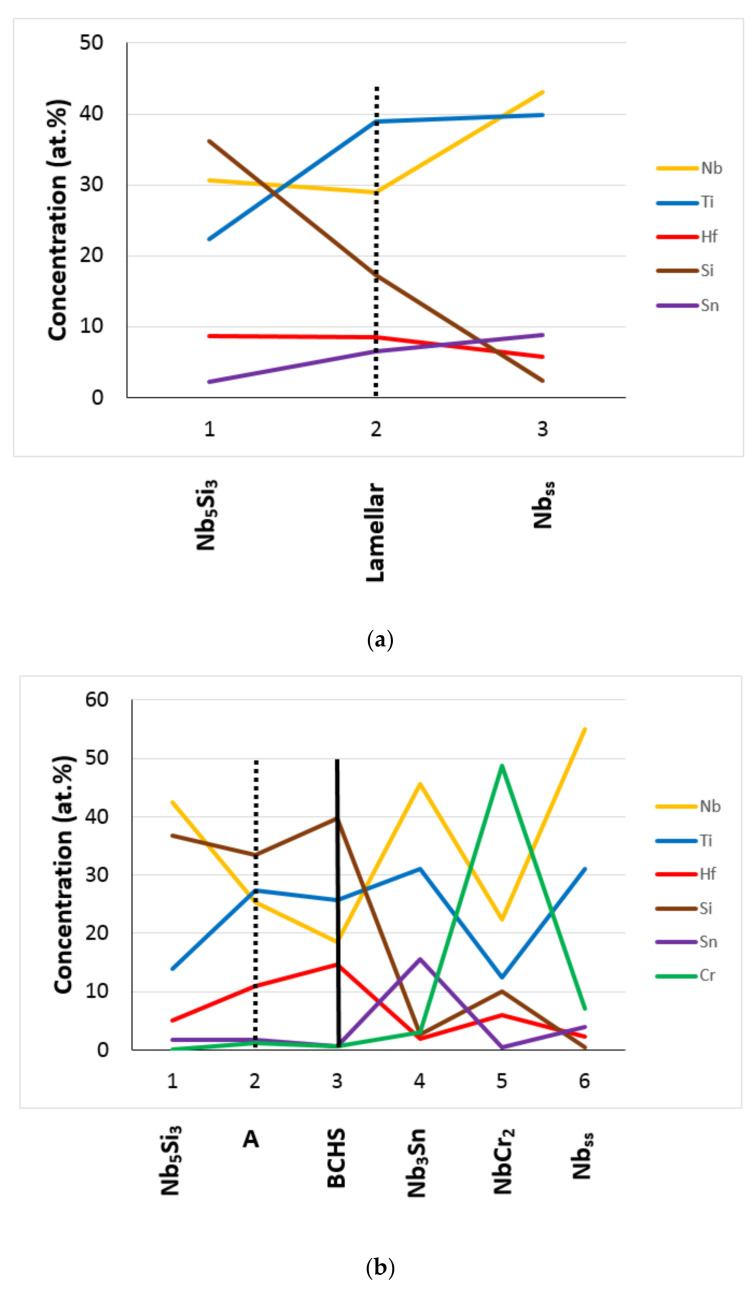
Average solute concentrations from Nb_5_Si_3_ towards the Nb_ss_ (**a**) for the lamellar microstructure in EZ2-AC and (**b**) the microstructure A in EZ6-HT2. In (**b**), BCHS = bright contrast hexagonal silicide. The data in this figure is not from a line scan; instead, it is quantitative analysis data from microstructures like those in the Figure 2 and Figure 8. The A15-Nb_3_X is shown as Nb_3_Sn.

**Figure 20:**
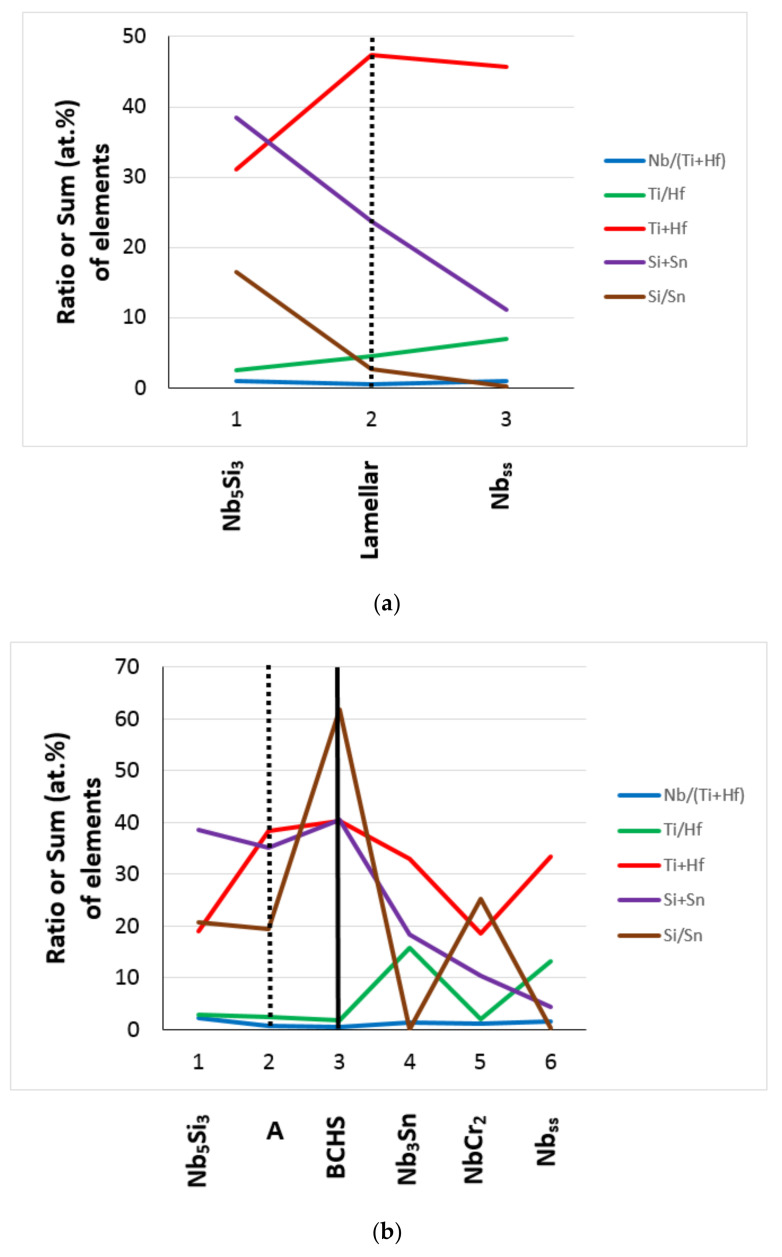
Ratios or sums of solutes based on average solute concentrations from Nb_5_Si_3_ towards the Nb_ss_ (**a**) for the lamellar microstructure in EZ2-AC and (**b**) the microstructure A in EZ6-HT2. In (**b**), BCHS = bright contrast hexagonal silicide. The data in this figure is not from a line scan; instead, it is quantitative analysis data from microstructures like those in the Figure 2 and Figure 8. The A15-Nb_3_X is shown as Nb_3_Sn.

Figure 19a shows the average solute concentrations from Nb_5_Si_3_ to the lamellar microstructure to the Nb_ss_ and Figure 20a shows the ratios or sums of solutes from Nb_5_Si_3_ to the lamellar microstructure to the Nb_ss_. From the tetragonal Nb_5_Si_3_ to the lamellar microstructure, (a) the Ti concentration increased, the Hf essentially did not change, and thus the Ti + Hf and Ti/Hf increased; (b) the Nb decreased slightly, and thus the Nb/(Ti + Hf) essentially did not change; and (c) the Si content decreased significantly, and the Sn increased slightly, and thus the Si+Sn and Si/Sn decreased. From the lamellar microstructure to the Nb_ss_, (d) the Ti and Hf increased and decreased slightly, respectively, while the Ti + Hf decreased slightly but the Ti/Hf continued to increase; (e) the Nb increased significantly, but the change of Nb/(Ti + Hf) was marginal; and (f) the increase of Sn and decrease of Si continued, and thus the Si + Sn sum and the Si/Sn ratio continued to decrease, the latter less than the former.

In other words, owing to the partitioning of solutes between the Nb_ss_ and Nb_5_Si_3_, in particular the partitioning of Ti and Hf, a phase transformation started from the Ti- and Hf-rich complex concentrated tetragonal Nb_5_Si_3_ that resulted in a complex concentrated lamellar microstructure, namely the eutectoid transformation tetragonal Nb_5_Si_3_ → Nb_ss_ + [Nb_5_Si_3_]_Hf and Ti rich_. The key solutes in this transformation were Hf and Ti. In the lamellar microstructure (i) the contrast of the Nb_ss_ was similar to that of the nearby solid solution, meaning the solid solution was Ti and Hf rich, and (ii) the contrast of the Nb_5_Si_3_ was similar or slightly brighter than that of the silicide adjacent to the lamellar microstructure. Brighter contrast Nb_5_Si_3_ means that it is richer in Hf silicide, which could be hexagonal γNb_5_Si_3_ depending on its Nb/(Ti + Hf) ratio [24]. The synergy of Hf and Ti with Sn in the alloys of this work promoted the γNb_5_Si_3_ (Figure 12). Thus, it is suggested that the Hf and Ti silicide in the lamellar microstructure was hexagonal γNb_5_Si_3_, that is, the aforementioned eutectoid phase transformation was Hf- and Ti-rich tetragonal αNb_5_Si_3_ → Nb_ss_ + γNb_5_Si_3_ with orientation relationships between Nb and αNb_5_Si_3_ and Nb and γNb_5_Si_3_ [29,57].

Now consider the lamellar microstructure on the left of number 17 in Figure 2b. It is suggested that the transformation nucleated at the interface between the Hf- and Ti-rich Nb_5_Si_3_ and the Ti- and Hf-rich Nb_ss_ (note that the Ti-rich Nb_ss_ was also richer in Hf than the “normal” Nb_ss_). The transformation front moved in a direction from the top right-hand corner to the bottom left-hand corner, and it would have stopped when the chemical composition of the Nb_5_Si_3_ grain was similar to that of the dark blocky Nb_5_Si_3_.

The lamellar microstructures were not stable in EZ2-HT, which would suggest that the proposed phase transformation had been completed after 100 h at 1500 °C and the hexagonal Hf-rich Nb_5_Si_3_ was a stable phase in EZ2, in agreement with the XRD data (Figure S1 in the Supplemental Data).

Eutectic and lamellar microstructure similar to those in EZ2-AC were also observed in the alloy NV1-AC (Nb-23Ti-5Si-5Al-5Hf-5V-2Cr-2Sn, [31]). In NV1-AC, the Nb_5_Si_3_ that was associated with the lamellar Nb_ss_ + Nb_5_Si_3_ had Ti + Hf = 35 at.% and Nb/(Ti + Hf) = 0.67, and was CC hexagonal Nb_5_Si_3_, whereas the lamellar microstructure, the composition of which corresponded to a CC lamellar microstructure [11], had Ti + Hf about 30 at.%, and Nb/(Ti + Hf) about 1.3. In EZ2-AC, the Nb_5_Si_3_ with Nb/(Ti + Hf) = 1 and Ti + Hf = 31.1 at.% (CC tetragonal Nb_5_Si_3_) was associated with a lamellar microstructure that was very Ti + Hf-rich (= 47.4 at.%) and had Nb/(Ti + Hf) = 0.6. Thus, comparison of the cast alloys NV1 and EZ2 shows that the Nb_5_Si_3_ silicides associated with the lamellar microstructure were different, CC hexagonal in NV1-AC and CC tetragonal in EZ2-AC, and the chemical compositions of the CC lamellar microstructures were also different. The eutectic in EZ2-AC has Ti + Hf = 31.9 at.% and Nb/(Ti + Hf) = 1.4, essentially the same as the lamellar in NV1-AC. The lamellar microstructure in NV1 was the product of a eutectic reaction plus some eutectoid transformation [31].

#### 4.2.11. Microstructure A in EZ6-HT

In EZ6-AC, the Nb_5_Si_3_ was surrounded by Nb_ss_. The contrast of the Hf-rich Nb_5_Si_3_ and the A15-Nb_3_X in EZ6-HT2 was very similar. The microstructure A formed adjacent to some Nb_5_Si_3_ grains but did not surround the whole grain (Figure 8b–d). The Nb_5_Si_3_ adjacent to microstructure A had Ti + Hf = 19 at.% and Nb/(Ti + Hf) = 2.25 (i.e., was tetragonal Nb_5_Si_3_), and microstructure A had Ti + Hf = 38.3 at.% and Nb/(Ti + Hf) = 0.66. A very thin bright contrast phase (BCP) was formed at the interface of microstructure A with A15-Nb_3_X (Figure 8c). This was actually bright contrast hexagonal silicide (BCHS). After the BCHS was the A15-Nb_3_X, and then the C14-NbCr_2_ Laves and the Nb_ss_ (Figure 8b–d). In the EZ6-HT2, the Hf-rich Nb_5_Si_3_ formed separate (distinct) grains from the “normal” Nb_5_Si_3_ (Figure 8b). The Hf-rich Nb_5_Si_3_ had Ti + Hf = 39.1 at.%, and Nb/(Ti + Hf) = 0.56, which were similar to those (a) of the BCHS of microstructure A (40.4 at.% and 0.46, respectively) and (b) the microstructure A (38.3 at.% and 0.66, respectively). The chemical compositions of microstructure A and the BCHS corresponded to complex concentrated phases.

Figure 19b shows the average solute concentrations, and Figure 20b shows the ratios or sums of solutes from Nb_5_Si_3_ to the microstructure A, to the BCHS, to the Nb_3_Sn, to the NbCr_2_, and to the Nb_ss_. From the tetragonal Nb_5_Si_3_, (a) the concentration of Hf increased to A, and then to the BCHS, whereas the concentration of Ti increased to A and then decreased slightly, and the concentration of Nb decreased to A and then to the BCHS, an thus the Nb/(Ti + Hf) and Ti/Hf ratios decreased to A and then to the BCHS, whereas the Ti + Hf sum increased markedly to A and then slightly to the BCHS; (b) the Si concentration decreased to A and then increased to the BCHS, that is, it exhibited the opposite trend compared with Ti, and the Sn concentration decreased after A to the BCHS, and thus both the Si+Sn sum and the Si/Sn ratio decreased to A and increased to the BCHS, with a remarkable increase of the Si/Sn ratio. The tetragonal Nb_5_Si_3_ was Cr free, and the Cr concentration increased slightly to A and then decreased to the BCHS. Thus, as microstructure A formed and the solutes partitioned between the Nb_ss_ and Nb_5_Si_3_, the microstructure became rich in Hf, Si, and Ti, and the BCHS formed.

From the BCHS to the A15-Nb_3_X, (c) the concentrations of Nb, Ti, Sn, and Cr increased, and those of Hf and Si decreased, and thus the Ti + Hf and Si + Sn sums and the Si/Sn ratio decreased, and the Ti/Hf and Nb/(Ti + Hf) ratios increased. On the other hand, (d) from the A15-Nb_3_X to the C14-NbCr_2_ Laves phase, the concentrations of Cr, Si, and Hf increased, and those of Nb, Ti, and Sn decreased, and thus the Ti + Hf and Si + Sn sums and the Ti/Hf ratio decreased, the Si/Sn ratio increased, but the Nb/(Ti + Hf) essentially did not change. In addition, (e) from the Laves phase to the Nb_ss_, the concentrations of Nb, Ti, and Sn increased, and those of Cr, Si and Hf decreased, and thus the Si/Sn ratio and the Si + Sn sum decreased, but the Ti + Hf sum and the Ti/Hf ratio increased, and the Nb/(Ti + Hf) ratio increased slightly. In other words, where the A15-Nb_3_X was stable, the microstructure was poorer in Hf and Si and richer in Nb, Sn, and Ti, but still poor in Cr compared with the BCHS. In the Cr-, Hf-, and Si-rich areas of the microstructure, the Laves phase was stable, and in the Nb-, Sn-, and Ti-rich areas, the Nb_ss_ was stable.

The microstructure A formed in areas of EZ6-HT2 where the Nb_ss_ was adjacent to the tetragonal Nb_5_Si_3_ prior to the heat treatment. The average composition of this Nb_ss_ in EZ6-AC was 45.6Nb-32.4Ti-1.8Si-8.7Cr-4.6Hf-6.9Sn, with Si + Sn = 8.7 at.%, Si/Sn = 0.26, Ti/Hf = 7, Ti + Hf = 37 at.%, and Nb/(Ti + Hf) = 1.23, compared with Si + Sn = 8.6 at.%, Si/Sn = 0.28, Ti/Hf = 7.6, Ti + Hf = 33.7 at.%, and Nb/(Ti + Hf) = 1.5 for the Nb_ss_ far away from the Nb_5_Si_3_ in EZ6-AC. In other words, the Nb_ss_ around tetragonal Nb_5_Si_3_ grains on some parts of which the microstructure A had formed was rich in Ti + Hf at the start of HT. In EZ6-HT2, the average chemical composition of the Nb_ss_ away from microstructure A was 55.1Nb-31.1Ti-0.5Si-7.1Cr-2.3Hf-3.9Sn, that is, the solid solution was poor in Si, and its Hf concentration was half that in the cast alloy, and it had Si + Sn = 4.4 at.%, significantly lower than the Nb_ss_ in the cast alloy; Si/Sn = 0.28, essentially the same with the cast alloy; Ti/Hf = 13.3, significantly higher compared with the cast alloy; Ti + Hf = 33.4 at.%, essentially the same as in the cast alloy; and Nb/(Ti + Hf) = 1.65, higher than the cast alloy. The data would suggest that partitioning of Hf, Si, Sn, and Ti was essential in the transformation that occurred in EZ6-HT2 and led to the formation of microstructure A. It is suggested (a) that the microstructure A was the product of the phase transformation [Nb_ss_]_Ti+Hf rich, Si+Sn rich_ → [Nb_ss_]_Si+Sn poor, Hf poor_ + [Nb_5_Si_3_]_Ti and Hf rich_ and (b) that owing to the promotion of the γNb_5_Si_3_ by the synergy of Hf and Ti with Sn in the alloys of this work (Figure 12), the [Nb_5_Si_3_]_Ti and Hf rich_ in the above phase transformation was γNb_5_Si_3_.

Comparison of the solute concentrations from the tetragonal Nb_5_Si_3_ to the lamellar microstructure in EZ2-AC and microstructure A in EZ6-HT2 shows similar trends for Nb, Si, and Ti and opposite trends for Hf and Sn. The lamellar microstructure in EZ2-AC and microstructure A in EZ6-HT2 were rich in Ti + Hf (47.4 at.% in EZ2-AC and 38.3 at.% in EZ6-HT2) and essentially had the same Nb/(Ti + Hf) ratio (0.6 in EZ2-AC and 0.66 in EZ6-HT2) (note also that the Ti + Hf sum and the Nb/(Ti + Hf) ratio of Hf-rich Nb_5_Si_3_ in NV1-AC and EZ6-HT2 were similar (35 at.% and 0.67 in NV1-AC [31] and 39.1 and 0.56 in EZ6-HT2)).

In EZ6-HT2, the growth of microstructure A was away from the silicide, and was “stopped” by the very Hf-rich BCHS (18.6Nb-25.7Ti-39.8Si-0.6Cr-14.7Hf-0.65Sn, with Si + Sn = 40.45 at.%, Si/Sn = 61.9, Ti/Hf = 1.75, Ti + Hf = 40.4 at.%, and Nb/(Ti + Hf) = 0.5), and the A15-Nb_3_X (45.6Nb-31.1Ti-2.7Si-3.1Cr-2Hf-15.6Sn, with Si + Sn = 18.3 at.%, Si/Sn = 0.2, Ti/Hf = 15.9, Ti + Hf = 33 at.%, and Nb/(Ti + Hf) = 1.4.

#### 4.2.12. Precipitation in Nb_5_Si_3_

In the heat-treated alloys EZ5 and EZ8, in which the Nb_ss_ was not a stable phase (Table 3), the A15-Nb_3_X precipitated in Nb_5_Si_3_ grains (Figure 4 and Figure 11). Both alloys contained Al and Ti. In the Ti-free and Al-containing alloys EZ4 and EZ7 [26], where the Nb_ss_ also was not a stable phase, no precipitates were observed in Nb_5_Si_3_. Thus, the precipitation of A15-Nb_3_X in EZ5-HT and EZ8-HT was attributed to the synergy of Al and Ti in said alloys. Precipitation of Nb_ss_ in Nb_5_Si_3_ has been reported by our research group in the Al- and Ti-containing but Sn-free heat-treated RM(Nb)ICs KZ2 (Nb-24Ti-18Si-8Cr-4Al), KZ5, and KZ7 [21], where it was attributed to the phase transformation βNb_5_Si_3_ → Nb_ss_ + αNb_5_Si_3_.

Modelling of the properties of metallic UHTMs, for example their toughness and creep (e.g., [56,58,59]), should not ignore the presence of microstructure A and/or precipitates of a second phase that can form respectively around or inside the Nb_5_Si_3_ after exposure to high temperature(s), as well as the change of properties at the interface between Nb_ss_ and Nb_5_Si_3_ [31].

#### 4.2.13. Hardness

**Figure 21:**
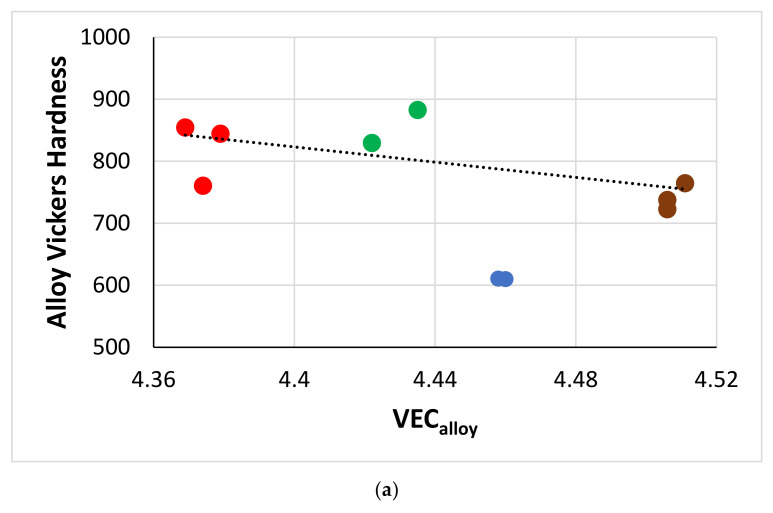

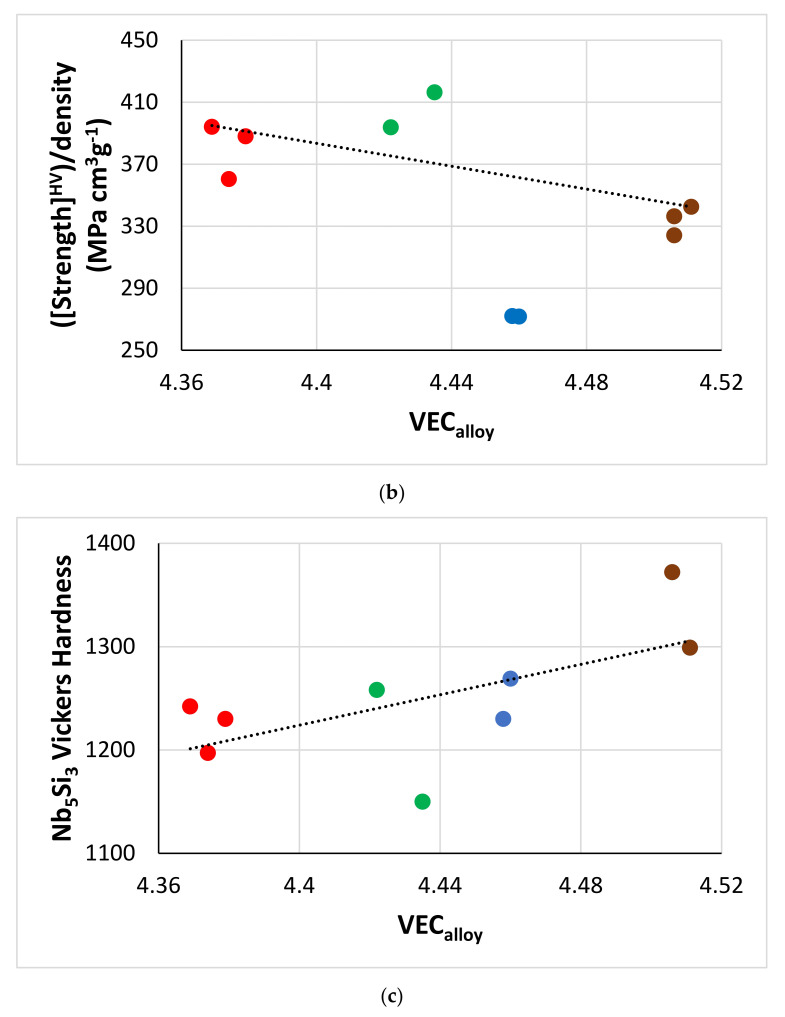

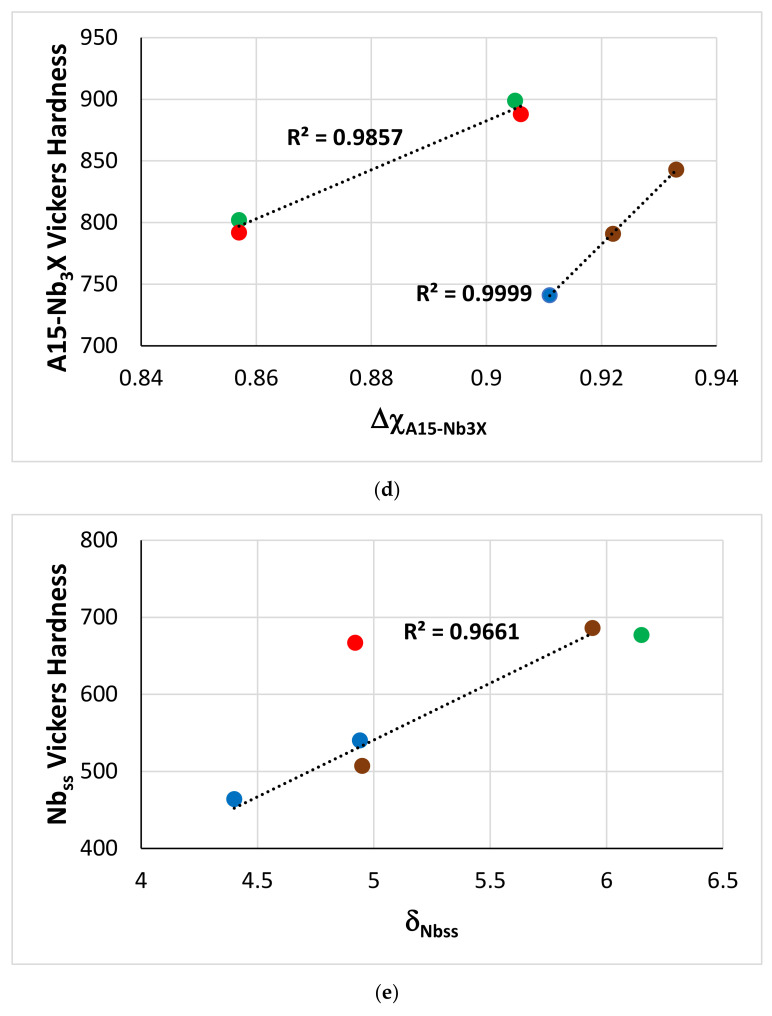

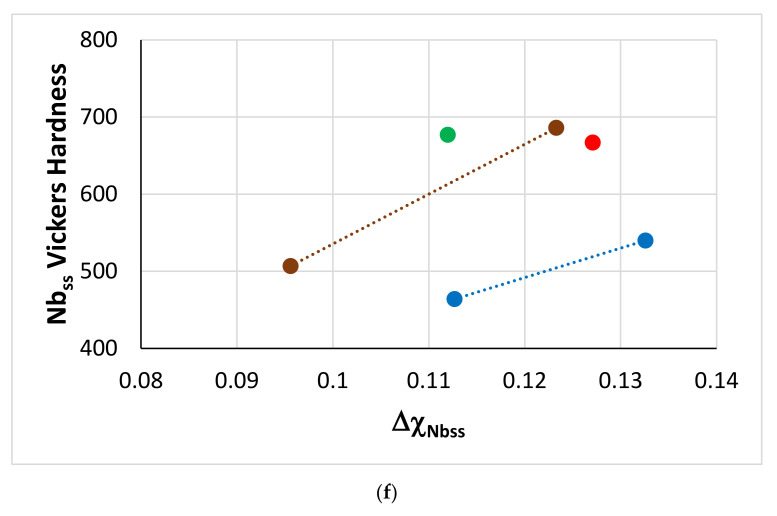
Hardness of (**a**) alloys, (**c**) Nb_5_Si_3_, (**d**) A15-Nb_3_X, (**e**) and (**f**) Nb_ss_, and (**b**) room-temperature-specific strength of the alloys of this work versus (**a**–**c**) the alloy parameter VEC, (**d**) the parameter Δχ of the A15-Nb_3_X, and (**e**) the parameter δ and (**f**) the parameter Δχ of Nb_ss_. In (**e**), the R^2^ value is for the data for the alloys EZ2 and EZ6, where the Nb_ss_ is a stable phase. Colors as follows: blue alloy EZ2, red EZ5, brown EZ6, and green EZ8. In (**a**–**f**), the dotted line is to “guide the eye”.

**Figure 22:**
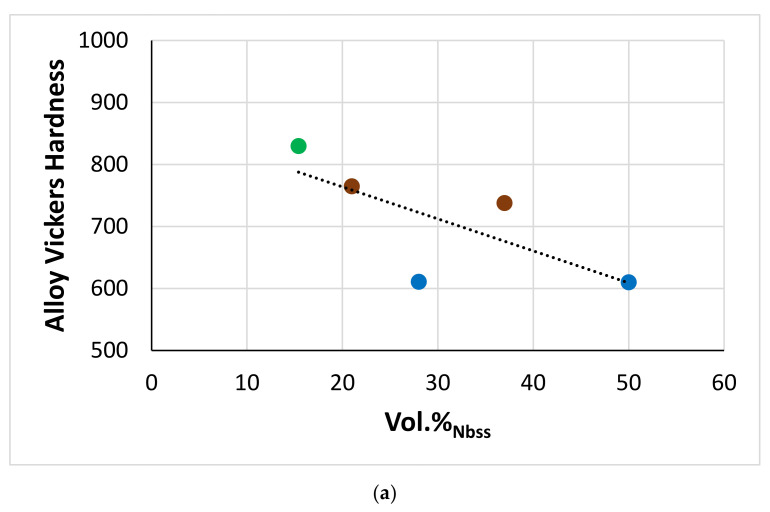

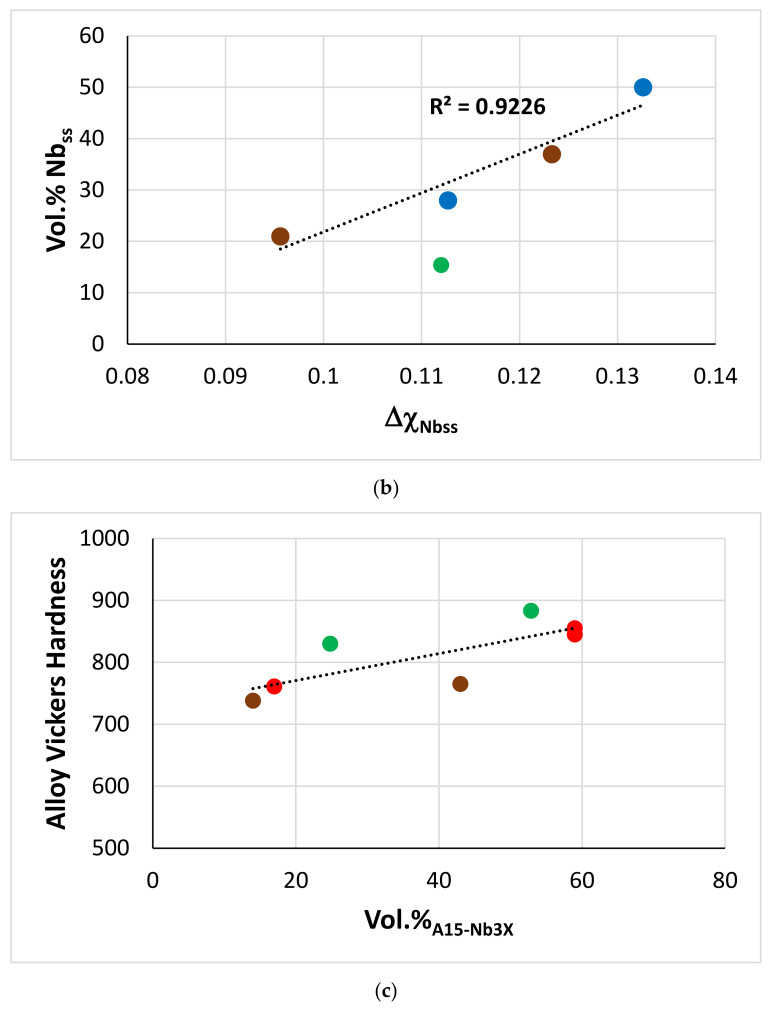

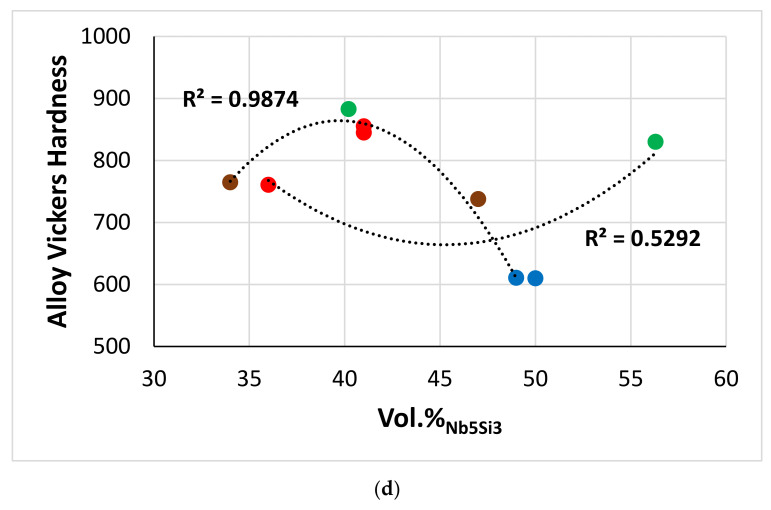
Alloy Vickers hardness versus vol.% (**a**) of Nb_ss_, (**c**) of A15-Nb_3_X, and (**d**) of Nb_5_Si_3_, and (**b**) vol.% Nb_ss_ versus Δχ_Nbss_. In (**d**), R^2^ = 0.5292 for the as-cast alloys and R^2^ = 0.9874 for the heat-treated alloys. In (**b**), the R^2^ value is for the alloys where the Nb_ss_ is a stable phase. Colors as follows: blue alloy EZ2, red EZ5, brown EZ6, and green EZ8. In (**a**,**c**), the dotted line is given to “guide the eye”.

The hardness of alloys and their phases as a function of alloy or phase parameters and volume fraction of phases is considered in Figure 21 and Figure 22. The hardness and room-temperature-specific strength calculated from hardness (σ_y_ = HV/(3ρ), where HV is alloy hardness and ρ is alloy density, see Table 1, [60]) of the alloys of this work increased with decreasing VEC_alloy_, in agreement with [1]. Figure 21c shows (1) that there is a correlation between the alloy parameter VEC and the hardness of Nb_5_Si_3_ (note that also there are relationships between VEC_alloy_ and VEC_Nb5Si3_ and VEC_alloy_ and Δχ_Nb5Si3_ for RM(Nb)ICs and RM(Nb)ICs/RCCAs, see [3]) and (2) that the hardness of the Nb_5_Si_3_ increased with the parameter VEC_alloy_ for the alloys of this work.

The hardness of the A15-Nb_3_X increased with its parameter Δχ and was higher for the alloys EZ5 and EZ8 (Figure 21d). The hardness of the Nb_ss_ increased with its parameters δ and Δχ (Figure 21e,f). The trend in Figure 21f is in agreement with previous work for RM(Nb)ICs and RM(Nb)ICs/RCCAs [3]. Compared with the alloy EZ2, the addition of Al and/or Cr in EZ5, EZ6, and EZ8 increased the hardness significantly. The hardness was highest for the as-cast alloys EZ5, EZ6, and EZ8.

Note the following: (i) the remarkable fit of data in Figure 21d, where the trend is in agreement with [13]; (ii) for the alloys where the Nb_ss_ was not stable, namely the alloys EZ5 and EZ8, the hardness of the Nb_ss_ was essentially similar, even though the δ_Nbss_ and Δχ_Nbss_ changed (Figure 21e,f); and (iii) the δ_Nbss_ was highest for the Nb_ss_ in EZ8-AC. The specific strength of the alloys was in the range 271.7 to 416.5 MPa cm^3^g^−1^. The specific strength of the alloys EZ5 and EZ8 (i.e., the two Al-containing alloys of this work), was higher than the room-temperature-specific strength of RCCAs and RHEAs reviewed in [2]. Furthermore, the specific strength of EZ8 was higher than that of B containing RM(Nb)ICs and RM(Nb)ICs/RCCAs [1,36,61].

The hardness of the alloys increased and decreased with increasing vol.% of A15-Nb_3_X and Nb_ss_, respectively (Figure 22). Note that the vol.% Nb_ss_ increased with increasing Δχ_Nbss_. The trend of the hardness of the AC and HT alloys versus their vol.% Nb_5_Si_3_ exhibited, respectively, a minimum and maximum, which correspond to about 45% and 40% Nb_5_Si_3_, respectively. The alloy EZ8-HT, in which the Nb_ss_ was not stable and the Nb_5_Si_3_ had the lowest hardness of the alloys of this work (Table 4), had the highest hardness and room-temperature-specific strength with vol.% Nb_5_Si_3_ = 40%, vol.% A15-Nb_3_X = 53%, and with VEC_alloy_ = 4.435 and Δχ_A15-Nb3X_ = 0.905.

#### 4.2.14. Comparison of the Synergy of Hf with B, Ge, or Sn in RM(Nb)ICs/RCCAs

In this section, we compare the effects of the synergy of solute additions, namely Al, Cr, Hf, Si, and Ti (group A elements), with each of the metal/metalloid elements B, Ge, or Sn (group B elements), which the alloy designer can use to design/select metallic UHTMs with a balance of properties. The comparison uses data for the alloys EZ8, ZF9 [35], and TT7 [36] (see Table 10). Note that all three alloys are RM(Nb)ICs/RCCAs and are based on the RM(Nb)IC alloy KZ5 (Nb-24Ti-18Si-5Al-5Cr [21]), with Hf plus one of the group B elements, namely Hf plus B or Hf plus Ge or Hf plus Sn addition, respectively, in TT7, ZF9, and EZ8.

1. Note that with the aforementioned solute elements, it is possible to have metallic UHTM RM(Nb)ICs/RCCAs (a) with density less than 7 g/cm^3^ and (b) with room-temperature-specific strength higher than that of the RCCAs and RHEAs reviewed in [2] (with the exception of the specific strength of TT7-HT).
2. Notice (c) that the Nb_ss_, which is a key phase for meeting specific property goals (namely fracture toughness, creep, and oxidation) or having a balance of properties, can be “controlled” (regarding stability and volume fraction) with synergies of specific solutes. For example, only that with the addition of Sn the Nb_ss_ was not stable (EZ8).
3. Note (d) that in metallic UHTM RM(Nb)ICs/RCCAs with the group A elements plus one of the group B elements, hexagonal Nb_5_Si_3_ can be stable together with the tetragonal α and β Nb_5_Si_3_. Indeed, as shown in Table 10, in all three alloys, tetragonal α and β Nb_5_Si_3_ and hexagonal γNb_5_Si_3_ were stable: the α, β, and γNb_5_Si_3_ in EZ8 and ZF9, and the tetragonal T2 (isomorphous with αNb_5_Si_3_) and hexagonal D8_8_ (isomorphous with γNb_5_Si_3_) silicides in TT7. Note that properties (e.g., Young’s modulus, CTE anisotropy, hardness, creep) of said silicides differ significantly and depend on alloying [34,62], and they change with the precipitation of a second phase in the silicide (see 7).
4. Notice (e) that in metallic UHTM RM(Nb)ICs/RCCAs with the group A elements plus Ge or Sn, the Nb_3_Si cannot be used to “engineer” the alloy microstructure, and that the same was the case with B addition [36].
5. Note (f) that for the alloys with the group A elements plus Sn, (i) the A15-Nb_3_X and C14-NbCr_2_ Laves phase, both of which play a key role in oxidation, can be stable, and (ii) the C14-NbCr_2_ can form a eutectic with the Nb_ss_ (data for EZ8).
6. Notice (g) that the chemical inhomogeneity of the microstructure can be significant in cast alloys depending on the choice of the group B solute element; (i) that with Sn, the MACSi is highest and MACX (X = Al,Hf,Sn) low; (ii) that with Ge, the MACSi is decreased, but MACTi and MACAl increased, while MACGe is low and similar to MACSn; and (iii) that with boron, the MACX (X = Cr,Si,Ti) is lowest, but MACB is increased compared with MACGe and MACSn. In other words, (iv) with boron as the group B solute element, chemical inhomogeneity in cast alloys is significantly reduced compared with Ge or Sn, even though the characterisation of the microstructure can be challenging with each of the group B elements [35,36].
7. Note (h) that mechanical properties and the oxidation of Nb_5_Si_3_ can be affected by the precipitation of second phase in Nb_5_Si_3_ when the chosen group B element is Sn or Ge, the second phase being A15-Nb_3_X with the former and Nb_ss_ with the latter addition (see data for EZ8 and ZF9 in Table 10).
8. Notice (i) that in metallic UHTM RM(Nb)ICs/RCCAs with the group A elements plus one of the group B elements, pest oxidation and scale spallation in the pest oxidation regime can be prevented, and (vi) that only with the choice of boron as a group B element was the scale spallation at 1200 °C avoided.

**Table 10:** Comparison of the RM(Nb)ICs/RCCAs EZ8, ZF9 (38Nb-24Ti-18Si-5Al-5Ge-5Hf-5Cr [35,63]), and TT7 (38Nb-24Ti-17Si-5Al-6B-5Cr-5Hf [36]).

Phase or Property	Solute	EZ8-AC	EZ8-HT	ZF9-AC	ZF9-HT	TT7-AC	TT7-HT
Nb_ss_		X	-	X	X	X	X
Ti rich Nb_ss_		-	-	-	-	X	-
Nb_5_Si_3_		X (t,h) **	X (t,h)	X (t,h)	X (t,h)	X (t,h)	X (t,h)
Ti rich Nb_5_Si_3_		X	X	X	X	X	-
Nb_3_Si		-	-	-	-	X	-
A15-Nb_3_X		X	X	-	-	-	-
Laves		X	X	X	-	-	-
ρ (g/cm^3^)		6.89		6.96		6.8	
Vol.% Nb_ss_		15	-	23 *	20 *	10	37
MACX (at.%)	Si	7.7		3.1		1.7	
	Ti	2.9		3.4		2.4	
	Al	1.1		2		-	
	Cr	2.3		2.1		2.2	
	Hf	1.1		-		-	
	B	-		-		2.8	
	Ge	-		1.1		-	
	Sn	1.4		-		-	
HV_alloy_		830	883	801	779	776	658
HV_Nb5Si3_		1258	1150	1495	1391	1340 (T2)	1200 (T2)
(σ_y_/ρ)_RT_ (MPa cm^3^g^−1^)		393.8	416.5	376	365.6	373	315
Precipitation in silicide			X Nb_3_X		X Nb_ss_		-
Pesting and scale spallation		No	No	No	No	No	No
Scale spallation at 1200 °C		X	X	X	X	No	No

* Calculated, ** t = tetragonal, h = hexagonal.

Furthermore, note that our research group has shown that the synergy of B and Sn, and Ge and Sn in KZ5-based but Hf-free alloys prevented both pest oxidation and scale spallation in the pest temperature range and scale spallation at high temperatures in the RM(Nb)ICs/RCCAs TT6 (Nb-24Ti-18Si-4Al-6B-5Cr-4Sn [36]) and OHS1 (Nb-24Ti-18Si-5Al-5Cr-5Ge-5Sn [41]).

## 5. Conclusions

A systematic study of the as-cast and heat-treated microstructures of three RM(Nb)ICs, namely the alloys EZ2, EZ5, and EZ6, and one RM(Nb)IC/RCCA, namely the alloy EZ8, and the hardness of alloys and phases was presented in this work. The four alloys were Nb-24Ti-18Si-based and had additions of Hf, Sn, and Ti that were in synergy simultaneously on their own (alloy EZ2) or with Al (EZ5) or Cr (EZ6) or Al and Cr (EZ8). All four alloys had density less than 7.3 g/cm^3^. The Nb_ss_ was stable in EZ2 and EZ6 and the C14-NbCr_2_ Laves phase in EZ6 and EZ8. In all four alloys, the A15-Nb_3_X (X = Al,Si,Sn), and tetragonal and hexagonal Nb_5_Si_3_ were stable. Eutectics of Nb_ss_ + Nb_5_Si_3_ and Nb_ss_ + C14-NbCr_2_ formed respectively in the cast alloys without and with Cr addition. In all four alloys, Nb_3_Si was not formed. In the heat-treated alloys EZ5 and EZ8, in which the Nb_ss_ was not stable, A15-Nb_3_X precipitated in Nb_5_Si_3_ grains. The chemical compositions of Nb_ss_ + C14-NbCr_2_ eutectics and some Nb_5_Si_3_ silicides and lamellar microstructures corresponded to high-entropy or complex concentrated phases (compositionally complex phases).

Microstructures and properties were considered from the perspective of the alloy design methodology NICE. The vol.% Nb_ss_ increased with increasing Δχ_Nbss_. The hardness of the alloys increased and decreased with increasing vol.% of A15-Nb_3_X and Nb_ss_, respectively. The hardness of the A15-Nb_3_X increased with its parameter Δχ, and the hardness of the Nb_ss_ increased with its parameters δ and Δχ. The room-temperature-specific strength of the alloys was in the range 271.7 to 416.5 MPa cm^3^g^−1^, and for EZ5 and EZ8, was higher than that of RCCAs and RHEAs. The effect of the synergy of Hf and Sn, or Hf and B or Hf and Ge on the macrosegregation of solutes, microstructures, and properties of RM(Nb)ICs/RCCAs was compared.

Comparison of the microstructures of the alloys of this work with those of alloys studied previously would suggest (i) that in RM(Nb)ICs and RM(Nb)ICs/RCCAs with Al, Cr, Hf, Si, Sn, and Ti addition, phase transformations that employ the Nb_3_Si silicide to engineer the microstructure cannot be used; (ii) that in Nb-18Si based RM(Nb)ICs where Hf, Sn, and Ti were in synergy, the vol.% of the Nb_ss_ + Nb_5_Si_3_ eutectic was controlled by Sn; (iii) that the synergy of Al with Sn in the presence of Hf made the formation of the Nb_ss_+Nb_5_Si_3_ eutectic sensitive to solidification conditions; (iv) that in the presence of Hf, the dominant elements that controlled the partitioning of solutes between the Nb_5_Si_3_ and the melt were Al and Ti, of which the former was the most potent; (v) that the synergy of Sn with Hf and Ti promoted the stability of the hexagonal Nb_5_Si_3_; and (vi) that regarding the stability of Nb_ss_, (a) Hf was part of the cause, (b) the concentration of Sn in the alloy was important in alloys where Al, Sn, and Ti were in synergy, and (c) the effect of the synergy of Al, Hf, and Sn with/without Ti on the stability of the Nb_ss_ could not be reversed with the addition of Cr.

## Figures and Tables

**Table 1:** Data for the density, hardness, and % area of phases for the alloys EZ2, EZ5, EZ6, and EZ8.

Alloy and Condition	Density (g/cm^3^)	Hardness (HV10)	% Area				
			Nb_5_Si_3_	Nb_3_Sn	Nb_ss_	Eutectic	NbCr_2_
EZ2-AC	7.34 ± 0.02 7.31–7.36	610 ± 27 570–653	50 ± 2	-	50 ± 2	-	-
EZ2-HT	7.34 ± 0.01 7.32–7.35	611 ± 20 579–645	49 ± 3	23 ± 5	28 ± 2	-	-
EZ5-AC *	6.9 ± 0.01 6.85–6.96	761 ± 19 740–792	36 ± 2	17 ± 2	-	46 ± 3	-
EZ5-HT1 *	7.12 ± 0.03 7.08–7.17	845 ± 21 819–870	41 ± 2	59 ± 2	-	-	-
EZ5-HT2 *	7.09 ± 0.02 7.07–7.15	855 ± 27 821–897	41 ± 2	59 ± 2	-	-	-
EZ6 AC	7.17 ± 0.01 7.14–7.20	738 ± 57 634–813	47 ± 2	14 ± 1	37 ± 2	-	2 ± 1
EZ6 HT1 *	7.29 ± 0.02 7.26–7.31	723 ± 48 661–802	-	-	-	-	-
EZ6 HT2 *	7.30 ± 0.01 7.29–7.33	765 ± 13 743–787	34 ± 1	43 ± 1	21 ± 1	-	2 ± 1
EZ8-AC	6.89 ± 0.01 6.84–6.92	830 ± 79 799–894	56.3 ± 2.4	24.8 ± 0.9	15.4 ± 2.1	-	3.5 ± 0.2
EZ8-HT	6.93 ± 0.01 6.89–7.03	883 ± 37 803–939	40.2 ± 1.1	52.9 ± 1.3	-	-	6.9 ± 0.2

* see text.

**Table 2:** Macrosegregation (at.%) MACX of addition X (X = Al, Cr, Hf, Si, Sn, Ti) in the AC alloys EZ2, EZ5, EZ6, and EZ8.

Alloy	MACX					
	Al	Cr	Hf	Si	Sn	Ti
EZ2	-	-	-	5	1	2.9
EZ5	-	-	-	3.7	1	1.3
EZ6	-	3.8	1.3	7	-	6.8
EZ8	1.1	2.3	1.1	7.7	1.4	2.9

**Table 3:** Summary of phases in the AC and HT alloys EZ2, EZ5, EZ6, and EZ8. Note that α, β, and γ refer to the structure of Nb_5_Si_3_ [38]: α and β, tetragonal Nb_5_Si_3_; γ, hexagonal Nb_5_Si_3_.

As-Cast Alloys				
Phase	EZ2	EZ5	EZ6	EZ8
Nb_ss_	X	X	X	X
Ti rich Nb_ss_	X	-	X	-
A15-Nb_3_X (X = Al,Si,Sn)	X	X	X	X
Nb_5_Si_3_	X, α, β	X, α, β, γ	X, α, β, γ	X, α, β, γ
Hf-rich Nb_5_Si_3_	X	X	X	X
Nb_ss_ + Nb_5_Si_3_ eutectic	X	X	-	-
C14-NbCr_2_ Laves	-	-	X	X
Nb_ss_ + NbCr_2_ eutectic	-	-	X	X
**Heat-Treated alloys**				
Phase	EZ2-HT	EZ5-HT1 *	EZ6-HT1 *	EZ8-HT
Nb_ss_	X	-	X	-
A15-Nb_3_X (X = Al,Si,Sn)	X	X	X	X
Nb_5_Si_3_	X, α, β, γ	X, α, β, γ	X, α, β, γ	X, α, β, γ
Hf-rich Nb_5_Si_3_	X	X	X	X
C14-NbCr_2_ Laves	-	-	X	X
		EZ5-HT2 *	EZ6-HT2 *	
Nb_ss_	-	-	X	-
A15-Nb_3_X (X = Al,Si,Sn)	-	X	X	-
Nb_5_Si_3_	-	X, α, β, γ	X, α, β, γ	-
Hf-rich Nb_5_Si_3_	-	X	X	-
C14-NbCr_2_ Laves	-	-	X	-

* See text.

**Table 4:** Hardness data of the phases in the alloys EZ2, EZ5, EZ6, and EZ8.

Alloy and Condition	Phase		
	Nb_5_Si_3_	A15-Nb_3_X	Nb_ss_
EZ2-AC	1269 ± 53 1123–1422	-	540 ± 26 519–582
EZ2-HT	1230 ± 46 1106–1394	741 ± 48 644–814	464 ± 31 395–511
EZ5-AC	1197 ± 41 1157–1247	792 ± 38 723–839	667 ± 78 543–720
EZ5-HT1	1230 ± 92 1037–1425	897 ± 25 849–930	-
EZ5-HT2	1242 ± 73 1058–1390	888 ± 21 843–915	-
EZ6 AC	1372 ± 68 1231–1552	791 ± 25 764–830	686 ± 14 648–713
EZ6 HT2	1299 ± 41 1128–1368	843 ± 21 804–895	507 ± 12 480–533
EZ8-AC	1258 ± 37 1190–1370	802 ± 25 764–830	677 ± 9 651–710
EZ8-HT	1150 ± 41 1089–1368	899 ± 21 816–930	-

**Table 5:** Lattice parameter (Å) of the bcc Nb_ss_ in Nb-18Si silicide-based alloys.

Alloy, Condition, and HT Temperature	Lattice Parameter (Å)
EZ1 AC (Nb-18Si-5Hf-5Sn) [26]	3.299
EZ1 HT1 1500 °C	3.325
EZ1 HT2 1500 °C	3.310
KZ3-AC (Nb-24Ti-18Si) [21]	3.253
KZ3-HT 1500 °C	3.296
NV6-AC (Nb-24Ti-18Si-5Sn) [39]	3.295
NV6-HT 1200 °C	3.293
EZ2-AC (Nb-24Ti-18Si-5Hf-5Sn)	3.292
EZ2-HT 1500 °C	3.287
KZ7 AC (Nb-24Ti-18Si-5Al) [21]	3.273
EZ5 AC (Nb-24Ti-18Si-5Al-5Hf-5Sn)	3.284
KZ4 AC (Nb-24Ti-18Si-5Cr) [21]	3.251
KZ4 HT 1500 °C	3.257
EZ6 AC (Nb-24Ti-18Si-5Cr-5Hf-5Sn)	3.256
EZ6 HT2 1200 °C	3.228

**Table 6:** Effect of alloying addition(s) (nominal composition, at.%) on the macrosegregation of Si (MACSi = C^Si^_max_ − C^Si^_min_, at.% [40]) in as-cast Nb-18Si silicide-based alloys with/without Ti addition.

Nominal Composition	Alloy	MACSi	Reference
Nb-18Si-24Ti-5Al-5Cr-5Sn	ZX8	10	[23]
Nb-18Si-24Ti-5Al-5Cr-5Hf-5Sn	EZ8	7.7	This work
Nb-18Si-24Ti-5Cr-5Sn	ZX4	7.3	[23]
Nb-18Si-24Ti-5Sn	NV6	7.1	[39]
Nb-18Si-24Ti-5Cr-5Hf-5Sn	EZ6	7	This work
Nb-18Si-24Ti-5Al-2Sn Nb-18Si-24Ti-5Al-5Sn	ZX5 ZX6	5.5 5.5	[22] [23]
Nb-18Si-24Ti-5Hf-5Sn	EZ2	5	This work
Nb-18Si-5Cr-5Hf-5Sn	EZ3	4.1	[26]
Nb-18Si-24Ti-5Al-5Cr-5Hf	JN1	4	[20]
Nb-18Si-5Al-5Hf-5Sn	EZ4	3.9	[26]
Nb-18Si-24Ti-5Al-5Hf-5Sn	EZ5	3.7	This work
Nb-18Si-24Ti-5Cr-2Sn	ZX3	3.6	[22]
Nb-18Si-24Ti-5Hf	YG3	3.3	[25]
Nb-18Si-24Ti-5Al-5Cr-2Sn	ZX7	3	[22]
Nb-18Si-5Hf-5Sn Nb-18Si-5Al-5Hf	EZ1 YG2	2.8 2.8	[26] [25]
Nb-18Si-5Al-5Sn Nb-18Si-5Sn Nb-18Si-5Cr-5Hf	EZ7 NV9 YG1	2.5 2.5 2.5	[26] [39] [25]
Nb-18Si-24Ti-5Al	KZ7	2.3	[21]
Nb-18Si-24Ti-5Cr	KZ4	1.9	[21]
Nb-18Si-24Ti-5Al-5Cr	KZ5	1.4	[21]

**Table 7:** Effect of the different alloying elements on the formation and composition (at.%) of Nb_ss_ + Nb_5_Si_3_ eutectic or Nb_ss_ + NbCr_2_ Laves phase eutectic in various as-cast RM(Nb)ICs.

Alloy Designation and Nominal Composition	Area of the AC Ingot Where Eutectic Formed			Composition of the Eutectic *
	Top	Bulk	Bottom	
				**Nb_ss_ + Nb_5_Si_3_**
**NV9** (Nb-18Si-5Sn) [41]	X	X	X	79.5M-20.5(Si + Sn)
**NV6** (Nb-24Ti-18Si-5Sn) [39]	X	X	X	79.5M-20.5(Si + Sn)
**YG1** (Nb-18Si-5Cr-5Hf) [25]	-	-	X	75.4M-24.6Si
**YG2** (Nb-18Si-5Al-5Hf) [25]	X	X	X	79M-21(Si + Al)
**YG3** (Nb-24Ti-18Si-5Hf) [25]	-	X	-	79M-21(Si + Sn)
**EZ1** (Nb-18Si-5Hf-5Sn) [26]	X	X	X	78.7M-21.3(Si + Sn)
**EZ2** (Nb-24Ti-18Si-5Hf-5Sn)	X	X	X	78.3M-21.7(Si + Sn)
**EZ4** (Nb-18Si-5Al-5Hf-5Sn) [26]	X	X	-	78.3M-21.7(Si + Sn + Al)
**EZ5** (Nb-24Ti-18Si-5Al-5Hf-5Sn)	-	X	-	76.7M-23.2(Si + Sn + Al)
				**Nb_ss_ + NbCr_2_**
**EZ3** (Nb-18Si-5Cr-5Hf-5Sn) [26]	X	X	X	50.4M-49.6(Cr + Si + Sn)
**EZ6** (Nb-24Ti-18Si-5Cr-5Hf-5Sn)	X	X	-	54M-46(Cr + Si + Sn)
**EZ8** (Nb-24Ti-18Si-5Al-5Cr-5Hf-5Sn)	X	X	X	50.9M-49.1(Cr + Si + Sn)

* M represents the total concentration of transition and refractory metals in the alloy.

**Table 8:** Comparison of compositions (at.%) of Nb_ss_, A15-Nb_3_X, Sn-rich A15-Nb_3_X, Nb_5_Si_3_, and Hf-rich Nb_5_Si_3_ in different as-cast Nb silicide-based alloys. For alloy nominal compositions and references, see the Table 6.

Phase	Solute Function	Alloy								
		EZ1	EZ2	EZ3	EZ4	EZ5	EZ6	EZ7	EZ8	NV9
Nb_ss_	Si/Sn	0.3	0.3	0.3			0.3			0.3
	Si/(Sn + Al)				0.2	0.2			0.2	
A15-Nb_3_X	Si + Sn (at.%)	17.2	18.8	18.2			18.6			18.2
	Si + Sn + Al				19.5	20.4		19.6	20.9	
Sn rich A15-Nb_3_X	Si + Sn + Al							19.9		
Nb_5_Si_3_	Si + Sn	38.4	38	38.6			37.8			36.8
	Si + Sn + Al				37.7	37.4		38.4	37.2	
Hf-rich Nb_5_Si_3_	Si + Sn	38.4	38.5	38.8			38.2			
	Si + Sn + Al				38.3	37			37.5	
Nb_ss_ + Nb_5_Si_3_ eutectic	Si + Sn	21.3	21.2							20.5
	Si + Sn + Al				21.7	23.2				

**Table 9:** Effect of the different alloying elements on the formation and composition (at.%) of the A15- Nb_3_X compound in various as-cast RM(Nb)ICs.

Alloy Designation and Nominal Composition	Area of the AC Ingot where the A15-Nb_3_X Formed			Composition of the A15-Nb_3_X *
	Top	Bulk	Bottom	
**NV9** (Nb-18Si-5Sn) [39]	X	X	X	82.2M-17.8 (Si + Sn)
**NV6** (Nb-24Ti-18Si-5Sn) [39]	X	X	X	81.8M-18.2 (Si + Sn)
**EZ1** (Nb-18Si-5Hf-5Sn) [26]	-	XX	-	82.6M-17.4 (Si + Sn) 81.9M-18.1 (Si + Sn)
**EZ2** (Nb-24Ti-18Si-5Hf-5Sn)	-	-	X	81.2M-18.8 (Si + Sn)
**EZ3** (Nb-18Si-5Cr-5Hf-5Sn) [26]	X	X	X	81.8M-18.2 (Si + Sn)
**EZ4** (Nb-18Si-5Al-5Hf-5Sn) [26]	X	X	X	80.5M-19.5 (Si + Sn + Al)
**EZ5** (Nb-24Ti-18Si-5Al-5Hf-5Sn)	-	X	X	79.6M-20.4 (Si + Sn + Al)
**EZ6** (Nb-24Ti-18Si-5Cr-5Hf-5Sn)	X	X	X	81.4M-18.6 (Si + Sn + Al)
**EZ7** (Nb-18Si-5Al-5Sn) [26]	X	X	X	80.4M-19.6 (Si + Sn + Al)
**EZ8** (Nb-24Ti-18Si-5Al-5Cr-5Hf-5Sn)	X	X	X	79.1M-20.9 (Si + Sn + Al)

* M is the total concentration of transition and refractory metals in the alloy.

## Data Availability

All the data for this work is given in the paper, other data cannot be made available to the public.

## Supplementary Materials

The following supporting information can be downloaded at: https://www.mdpi.com/article/10.3390/ma15134596/s1. Table S1: EPMA data (at%) of the as-cast and heat-treated alloy EZ2, Table S2: EPMA data (at%) of the as-cast and heat-treated alloy EZ5, Table S3: EPMA data (at%) of the as-cast and heat-treated alloy EZ6, Table S4: EPMA data (at%) of the as-cast and heat-treated alloy EZ8, Figure S1: X-ray diffractograms of the as-cast and heat-treated alloy EZ2, Figure S2: X-ray diffractograms of the as-cast and heat-treated alloy EZ5, Figure S3: X-ray diffractograms of the as-cast and heat-treated alloy EZ6, Figure S4: X-ray diffractograms of the as-cast and heat-treated alloy EZ8.

## References

[1] Tsakiropoulos P. (2020). Alloys for application at ultra-high temperatures: Nb-silicide in situ composites. Prog. Mater. Sci..

[2] Senkov O.N., Miracle D.B., Chaput K.J. (2018). Development and exploration of refractory high entropy alloys-A review. J. Mater. Res..

[3] Tsakiropoulos P. (2021). Refractory Metal (Nb) Intermetallic Composites, High Entropy Alloys, Complex Concentrated Alloys and the Alloy Design Methodology NICE: Mise-en-scène Patterns of Thought and Progress. Materials.

[4] Pickering E.J., Jones N.G. (2016). High-Entropy Alloys: A Critical Assessment of Their Founding Principles and Future Prospects. Int. Mater. Rev..

[5] Murty B.S., Yeh J.-W., Ranganathan S., Bhattacharjee P.P. (2019). High-Entropy Alloys.

[6] Cantor B., Chang I.T.H., Knight P., Vincent A.J.B. (2004). Microstructural development in equiatomic multicomponent alloys. Mater. Sci. Eng. A.

[7] Yeh J.-W., Chen S.K., Lin S.-J., Gan J.-Y., Chin T.-S., Shun T.-T., Tsau C.-H., Chang S.-Y. (2004). Nanostructured High-Entropy Alloys with Multiple Principal Elements: Novel Alloy Design Concepts and Outcomes. Adv. Eng. Mater..

[8] Shah D.M., Antolovich S.D., Stusrud R.W., MacKay R.A., Anton D.L., Khan T., Kissinger R.D., Klastrom D.L. (1992). MoSi_2_ and Other Silicides as High Temperature Structural Materials.

[9] Hemker K.J., Dimiduk D.M., Clemens H., Darolia R., Inui H., Larsen J.M., Sikka V.K., Thomas M., Whittenberger J.D. (2001). Structural Intermetallics.

[10] (2021). Defining Pathways for Realizing the Revolutionary Potential of High Entropy Alloys, A Study Organised by The Minerals, Metals & Materials Society (TMS). www.tms.org/HEApathways.

[11] Tsakiropoulos P. (2022). Refractory metal intermetallic composites, High entropy alloys and Complex concentrated alloys: A route to selecting substrate alloys, and bond coat alloys for environmental coatings. Materials.

[12] Tsakiropoulos P. (2018). On Nb Silicide Based Alloys: Alloy Design and Selection. Materials.

[13] Tsakiropoulos P. (2018). Alloying and properties of C14-NbCr_2_ and A15-Nb_3_X (X = Al, Ge, Si, Sn) in Nb-silicide based alloys. Materials.

[14] Vellios N., Tsakiropoulos P. (2007). The role of Fe and Ti additions in the microstructure of Nb–18Si–5Sn silicide-based alloys. Intermetallics.

[15] Vellios N., Tsakiropoulos P. (2010). Study of the role of Fe and Sn additions in the microstructure of Nb–24Ti–18Si–5Cr silicide based alloys. Intermetallics.

[16] Knittel S., Mathieu S., Vilasi M. (2014). Effect of tin addition on Nb–Si-based in situ composites. Part I: Structural modifications. Intermetallics.

[17] Li X., Chen H., Sha J., Hu Z. (2010). The effects of melting technologies on the microstructures and properties of Nb–16Si–22Ti–2Al–2Hf–17Cr alloy. Mater. Sci. Eng. A.

[18] Yuan S., Jia L., Su L., Ma L., Zhang H. (2013). The microstructure evolution of directionally solidified Nb-22Ti-14Si-4Cr-2Al-2Hf alloy during heat treatment. Intermetallics.

[19] Fei D., Lina J., Sainan Y., Linfen S., Junfei W., Hu Z. (2014). Microstructure evolution of a hypereutectic Nb–Ti–Si–Cr–Al–Hf alloy processed by directional solidification. Chin. J. Aeronaut..

[20] Nelson J., Ghadyani M., Utton C., Tsakiropoulos P. (2018). A Study of the Effects of Al, Cr, Hf, and Ti Additions on the Microstructure and Oxidation of Nb-24Ti-18Si Silicide Based Alloys. Materials.

[21] Zelenitsas K., Tsakiropoulos P. (2005). Study of the role of Cr and Al additions in the microstructure of Nb-Ti-Si in situ composites. Intermetallics.

[22] Xu Z., Utton C., Tsakiropoulos P. (2018). A study of the effect of 2 at.% Sn on the microstructure and isothermal oxidation at 800 and 1200 °C of Nb-24Ti-18Si based alloys with Al and/or Cr additions. Materials.

[23] Xu Z., Utton C., Tsakiropoulos P. (2020). A study of the effect of 5 at.% Sn on the microstructure and isothermal oxidation at 800 and 1200 °C of Nb-24Ti-18Si based alloys with Al and/or Cr additions. Materials.

[24] Bewlay B.P., Sitzman S.D., Brewer L.N., Jackson M.R. (2004). Analyses of eutectoid phase transformations in Nb-silicide in-situ composites. Microsc. Microanal..

[25] Grammenos I., Tsakiropoulos P. (2010). Study of the role of Al, Cr and Ti additions in the microstructure of Nb-18Si-5Hf silicide based alloys. Intermetallics.

[26] Zacharis E., Utton C., Tsakiropoulos P. (2018). A study of the effects of Hf and Sn on the microstructure, hardness and oxidation of Nb-18Si silicide based alloys without Ti addition. Materials.

[27] Jackson M.R., Bewlay B.P., Zhao J.-C. (2002). Niobium Silicide Based Composites Resistant to Low Temperature Pesting. U.S. Patent.

[28] Geng J., Tsakiropoulos P., Shao G. (2007). A Study of the Effects of Hf and Sn Additions on the micro-structure of Nb_ss_/Nb_5_Si_3_ based in situ composites. Intermetallics.

[29] Cheng G., He L. (2011). Microstructure evolution and room temperature deformation of a unidirectionally solidified Nb-22Ti-16Si-3Ta-2Hf-7Cr-3Al-0.2Ho (at.%) alloy. Intermetallics.

[30] Kang Y., Qu S., Song J., Huang Q., Han Y. (2012). Microstructure and mechanical properties of Nb–Ti–Si–Al–Hf–xCr–yV multi-element in situ composite. Mater. Sci. Eng. A.

[31] Vellios N., Keating P., Tsakiropoulos P. (2021). On the Microstructure and Properties of the Nb-23Ti-5Si-5Al-5Hf-5V-2Cr-2Sn (at.%) Silicide-Based Alloy—RM(Nb)IC. Metals.

[32] Bewlay B.P., Jackson M.R., Gigliotti M.F.X. (2002). Niobium Silicide High Temperature In Situ Composites. Intermetallic Compounds—Principles and Practice.

[33] Jackson M.R., Bewlay B.P., Briant C.L. (2002). Creep Resistant Nb-Silicide Based Two Phase Composites. U.S. Patent.

[34] Tsakiropoulos P. (2018). On the Alloying and Properties of Tetragonal Nb5Si3 in Nb-Silicide Based Alloys. Materials.

[35] Li Z., Tsakiropoulos P. (2019). On the microstructure and hardness of the Nb-24Ti-18Si-5Al-5Cr-5Ge and Nb-24Ti-18Si-5Al-5Cr-5Ge-5Hf (at.%) silicide based alloys. Materials.

[36] Thandorn T., Tsakiropoulos P. (2021). On the microstructure and properties of Nb-Ti-Cr-Al-B-Si-X (X = Hf, Sn, Ta) refractory complex concentrated alloys. Materials.

[37] Cullity B.D. (1978). Elements of X-ray Diffraction.

[38] Schlesinger M.E., Okamoto H., Gokhale A.B., Abbaschian R. (1993). The Nb-Si (Niobium-Silicon) System. J. Phase Equilibria.

[39] Vellios N., Tsakiropoulos P. (2007). The role of Sn and Ti additions in the microstructure of Nb–18Si base alloys. Intermetallics.

[40] Tsakiropoulos P. (2014). On the macrosegregation of silicon in niobium silicide based alloys. Intermetallics.

[41] Hernandez-Negrete O., Tsakiropoulos P. (2020). On the microstructure and isothermal oxidation at 800 and 1200 °C of the Nb-24Ti-18Si-5Al-5Cr-5Ge-5Sn (at.%) silicide based alloy. Materials.

[42] Subramanian P.R., Mendiratta M.G., Dimiduk D.M. (1994). Microstructures and mechanical behaviour of Nb-Ti base beta + silicide alloys. Mat. Res. Soc. Symp. Proc..

[43] Liang H., Chang Y.A. (1999). Thermodynamic modelling of the Nb-Si-Ti ternary system. Intermetallics.

[44] Geng T., Li C., Bao J., Zhao X., Du Z., Guo C. (2009). Thermodynamic assessment of the Nb-Si-Ti system. Intermetallics.

[45] Gigolotti J.C.J., Coelho G.C., Nunes C.A., Suzuki P.A., Joubert J.-M. (2017). Experimental evaluation of the Nb Si Ti system from as-cast alloys. Intermetallics.

[46] Hunt C.R., Raman A. (1968). Alloy chemistry of SIGMA-BETA-U-related phases. PT. 1. Extension of μ and occurrence of μ prime-phases in the ternary systems Nb-Ta-X-Al (X equals Fe, Co, Ni, Cu, Cr, Mo). Z. Metallkd..

[47] McCaughey C., Tsakiropoulos P. (2018). Type of Primary Nb_5_Si_3_ and Precipitation of Nb_ss_ in αNb_5_Si_3_ in a Nb-8.3Ti-21.1Si-5.4Mo-4W-0.7Hf (at.%) Near Eutectic Nb-Silicide-Based Alloy. Materials.

[48] Zhao J., Utton C., Tsakiropoulos P. (2020). On the Microstructure and Properties of Nb-12Ti-18Si-6Ta-5Al-5Cr-2.5W-1Hf (at.%) Silicide-Based Alloys with Ge and Sn Additions. Materials.

[49] Zhao J., Utton C., Tsakiropoulos P. (2020). On the Microstructure and Properties of Nb-18Si-6Mo-5Al-5Cr-2.5W-1Hf Nb-Silicide Based Alloys with Ge, Sn and Ti Additions (at.%). Materials.

[50] Geng J., Tsakiropoulos P. (2007). A study of the microstructures and oxidation of Nb-Si-Cr-Al-Mo in-situ composites alloyed with Ti, Hf and Sn. Intermetallics.

[51] Jackson M.R. (1994). NbTiAlCrHf Alloys and Structures. U.S. Patent.

[52] Davidson D.L., Chan K.S. (1999). The fatigue and fracture resistance of a Nb-Cr-Ti-Al alloy. Met. Mater. Trans. A.

[53] Davidson D.L., Chan K.S., Loloee R., Crimp M.A. (2000). Fatigue and fracture toughness of a Nb-Ti-Cr-Al-X single-phase alloy at ambient temperature. Met. Mater. Trans. A.

[54] Murayama Y., Hanada S. (2002). High temperature strength, fracture toughness and oxidation resistance of Nb-Si-Al-Ti multiphase alloys. Sci. Technol. Adv. Mater..

[55] Bewlay B.P., Jackson M.R., Zhao J.-C., Subramanian P., Mendiratta M.G., Lewandowski J. (2003). Ultrahigh-Temperature Nb-Silicide-Based Composites. MRS Bull..

[56] Chan K.S. (1996). The fracture toughness of niobium-based,in situ composites. Met. Mater. Trans. A.

[57] Cheng G., Tian Y., He L., Guo J. (2009). Orientation relationship and interfacial structure betweenα-Nb5Si3and Nb solid solution in the eutectic lamellar structure. Philos. Mag..

[58] Chan K.S. (2002). Modelling creep behaviour of niobium silicide in-situ composites. Mater. Sci. Eng. A.

[59] Henshall G.A., Subramanian P.R., Strum M.J., Mendiratta M.G. (1997). Continuum predictions of deformation in composites with two creping phases–II: Nb_5_Si_3_/Nb composites. Acta Mater..

[60] Galanov B.A., Milman Y.V., Chugunova S.I., Goncharova I.V., Voskoboinik I.V. (2017). Application of the Improved Inclusion Core Model of the Indentation Process for the Determination of Mechanical Properties of Materials. Crystals.

[61] Thandorn T., Tsakiropoulos P. (2021). The effect of Boron on the microstructure and properties of refractory metal intermetallic composites (RM(Nb)ICs) based on Nb-24Ti-xSi (x = 16, 17 or 18 at.%) with additions of Al, Cr or Mo. Materials.

[62] Papadimitriou I., Utton C., Tsakiropoulos P. (2017). The impact of Ti and temperature on the stability of Nb_5_Si_3_ phases: A first-principles study. Sci. Technol. Adv. Mater..

[63] Li Z., Tsakiropoulos P. (2019). The Effect of Ge Addition on the Oxidation of Nb-24Ti-18Si Silicide Based Alloys. Materials.

