# Supplementary material for: A Study of the Effects of Hf and Sn on the Microstructure, Hardness and Oxidation of Nb-18Si Silicide-Based Alloys-RM(Nb)ICs with Ti Addition and Comparison with Refractory Complex Concentrated Alloys (RCCAs)
Source: Materials (Basel). 2022 Jun 30;15(13):4596. doi: 10.3390/ma15134596 (PMC9267193; doi:10.3390/ma15134596)
Supplement: Supplementary file 1 [file materials-15-04596-s001.zip › materials-1761138-supplementary.pdf]

**Table S1:** EPMA data (at%)of the as-cast and heat-treated alloy EZ2.

| Condition, area in the ingot and phase | Nb | Ti | Si | Hf | Sn |
|---|---|---|---|---|---|
| **As cast** | | | | | |
| Top | 44.8–46.7<br>45.8 ± 0.7 | 23.4–25.9<br>24.4 ± 0.9 | 16.5–21.2<br>19.5 ± 1.8 | 5.2–5.3<br>5.3 ± 0.1 | 4.9–5.6<br>5.0 ± 0.4 |
| Bulk | 46.1–47.4<br>46.5 ± 0.5 | 23.0–25.3<br>24.3 ± 0.9 | 16.3–21.0<br>18.8 ± 1.6 | 5.1–5.3<br>5.2 ± 0.1 | 4.7–5.7<br>5.2 ± 0.4 |
| Bottom | 45.8–46.0<br>45.9 ± 0.1 | 23.0–25.3<br>23.5 ± 0.9 | 18.0–21.3<br>20.4 ± 1.3 | 5.1–5.3<br>5.3 ± 0.1 | 4.8–5.6<br>4.9 ± 0.4 |
| $Nb_{ss}$ | 47.3–56.6<br>53.4 ± 2.8 | 29.3–35.9<br>31.8 ± 2.2 | 1.9–3.1<br>2.4 ± 0.3 | 4.2–5.1<br>4.5 ± 0.3 | 7.0–8.9<br>7.9 ± 0.5 |
| Ti rich $Nb_{ss}$ | 31.4–39.7<br>35.9 ± 4.3 | 43.2–49.1<br>45.7 ± 3.3 | 2.0–3.0<br>2.5 ± 0.3 | 6.1–7.4<br>6.7 ± 0.5 | 8.5–10.2<br>9.2 ± 0.5 |
| A15-$Nb_3X$ (X=Si,Sn) | 48.1–55.5<br>50.5 ± 2.0 | 23.8–29.5<br>27.7 ± 1.6 | 5.0–6.4<br>5.6 ± 0.4 | 2.7–3.2<br>3.0 ± 0.2 | 11.5–14.3<br>13.2 ± 0.8 |
| $Nb_5Si_3$ | 42.2–44.1<br>43.2 ± 0.5 | 13.1–17.0<br>14.2 ± 1.1 | 34.9–37.5<br>36.4 ± 0.5 | 3.7–5.0<br>4.6 ± 0.4 | 1.3–1.9<br>1.6 ± 0.1 |
| Hf rich $Nb_5Si_3$ | 25.5–40.3<br>35.8 ± 0.6 | 17.8–25.8<br>18.8 ± 0.3 | 35.6–38.5<br>36.7 ± 0.2 | 5.4–9.5<br>6.9 ± 0.2 | 0.8–2.3<br>1.8 ± 0.1 |
| **Heat-treated - 1500 °C/100 h** | | | | | |
| Bulk | 44.1–47.0<br>45.8 ± 0.8 | 22.0–24.6<br>23.6 ± 0.8 | 18.0–22.2<br>20.3 ± 1.4 | 5.1–5.7<br>5.3 ± 0.2 | 4.5–5.6<br>5.0 ± 0.3 |
| $Nb_{ss}$ | 56.5–60.3<br>58.3 ± 0.9 | 29.6–32.2<br>31.1 ± 0.7 | 1.1–1.6<br>1.3 ± 0.1 | 3.7–4.2<br>4.0 ± 0.1 | 4.7–6.0<br>5.3 ± 0.3 |
| A15-$Nb_3X$ (X=Si,Sn) | 4.8–6–51.0<br>49.5 ± 0.7 | 27.7–30.2<br>29.0 ± 0.7 | 4.1–4.9<br>4.5 ± 0.2 | 2.6–3.1<br>2.8 ± 0.1 | 13.8–14.6<br>14.2 ± 0.2 |
| $Nb_5Si_3$ | 37.4–43.5<br>41.5 ± 2.0 | 13.3–17.3<br>14.9 ± 1.4 | 36.3–37.2<br>36.9 ± 0.3 | 3.9–6.3<br>5.1 ± 0.6 | 1.4–1.9<br>1.6 ± 0.2 |
| Hf rich $Nb_5Si_3$ | 24.0–25.3<br>24.6 ± 0.3 | 24.4–25.7<br>25.0 ± 0.3 | 38.9–39.6<br>39.2 ± 0.2 | 10.3–11.3<br>10.8 ± 0.2 | 0.3–0.6<br>0.4 ± 0.1 |

**Table S2:** EPMA data (at%) of the as-cast and heat-treated alloy EZ5.

| Condition, area in the ingot and phase | Nb | Ti | Si | Al | Hf | Sn |
|---|---|---|---|---|---|---|
| **As cast** | | | | | | |
| Top | 41.5–41.9<br>41.6 ± 0.1 | 23.5–23.7<br>23.6 ± 0.1 | 20.0–21.0<br>20.2 ± 0.2 | 4.7–4.8<br>4.7 ± 0.1 | 4.9–5.1<br>5 ± 0.1 | 4.9–5.0<br>4.9 ± 0.1 |
| Bulk | 41.6–42.7<br>42.0 ± 0.3 | 23.6–24.6<br>24.2 ± 0.2 | 17.3–20.1<br>18.8 ± 0.3 | 4.5–5.0<br>4.8 ± 0.1 | 5.0–5.4<br>5.2 ± 0.1 | 4.6–5.6<br>5 ± 0.1 |
| Bottom | 42.3–42.6<br>42.4 ± 0.1 | 23.4–24.7<br>24 ± 0.2 | 17.5–20.2<br>18.8 ± 0.4 | 4.4–5.1<br>4.7 ± 0.1 | 4.9–5.5<br>5.2 ± 0.1 | 4.7–5.2<br>4.9 ± 0.1 |
| $Nb_{ss}$ | 39.7–51.2<br>46.0 ± 2.1 | 29.5–37.7<br>33.9 ± 1.1 | 1.4–6.6<br>2.5 ± 0.9 | 5.4–7.2<br>6.2 ± 0.4 | 3.6–7.1<br>5.2 ± 0.6 | 3.6–10.2<br>6.2 ± 1.0 |
| A15-$Nb_3X$ (X=Al,Si,Sn) | 49.3–55.6<br>53.3 ± 0.8 | 21.2–26.9<br>23.6 ± 0.6 | 3.8–5.6<br>4.4 ± 0.3 | 4.4–5.9<br>5.3 ± 0.3 | 2.2–3.5<br>2.7 ± 0.4 | 9.1–11.5<br>10.7 ± 0.5 |
| $Nb_5Si_3$ | 37.1–43.9<br>41.0 ± 0.7 | 16.1–21.4<br>17.5 ± 0.9 | 29.7–33.3<br>31.4 ± 0.7 | 2.3–4.7<br>3.5 ± 0.6 | 3.5–4.6<br>4.1 ± 0.3 | 1.5–3.4<br>2.5 ± 0.5 |
| Ti & Hf rich $Nb_5Si_3$ | 25.6–39.1<br>31.4 ± 1.4 | 20.0–26.7<br>23.1 ± 0.5 | 28.9–34.2<br>32.1 ± 0.6 | 3.6–4.7<br>3.5 ± 0.1 | 4.6–10.5<br>8.5 ± 0.5 | 0.7–4.1<br>1.4 ± 0.7 |
| $Nb_{ss}$-Ti & Hf rich $Nb_5Si_3$ Eutectic | 37.1–40.3<br>38.9 ± 0.4 | 29.9–31.3<br>30.6 ± 0.2 | 11.1–15.3<br>13.4 ± 0.6 | 5.2–5.7<br>5.4 ± 0.1 | 6.7–8.0<br>7.3 ± 0.2 | 3.9–5.0<br>4.4 ± 0.3 |
| **Heat-treated - 1500 °C/100 h (EZ5-HT1)** | | | | | | |
| Bulk | 41.5–43.5<br>42.5 ± 0.3 | 24.0–24.5<br>24.3 ± 0.1 | 17.3–19.2<br>18.3 ± 0.4 | 4.4–4.8<br>4.6 ± 0.1 | 5.1–5.8<br>5.4 ± 0.1 | 4.6–5.3<br>4.9 ± 0.1 |
| A15-$Nb_3X$ (X=Al,Si,Sn) | 51.4–54.2<br>52.6 ± 0.4 | 24.6–26.8<br>25.6 ± 0.4 | 3.9–4.9<br>4.5 ± 0.3 | 5.9–6.6<br>6.2 ± 0.1 | 1.6–2.6<br>2.1 ± 0.2 | 8.5–9.4<br>9.0 ± 0.2 |
| $Nb_5Si_3$ | 38.4–40.9<br>39.1 ± 0.4 | 16.9–18.5<br>17.4 ± 0.3 | 33.7–35.6<br>34.9 ± 0.3 | 1.9–2.3<br>2.1 ± 0.1 | 4.1–6.0<br>5.3 ± 0.3 | 1.0–1.6<br>1.2 ± 0.2 |
| Ti & Hf rich $Nb_5Si_3$ | 24.7–27.2<br>26.0 ± 0.5 | 23.7–25.2<br>24.6 ± 0.4 | 34.2–35.9<br>35.4 ± 0.4 | 2.9–3.7<br>3.4 ± 0.2 | 9.4–11.3<br>10.1 ± 0.4 | 0.3–0.8<br>0.5 ± 0.1 |
| **Heat-treated - 1500 °C/200 h (EZ5-HT2)** | | | | | | |
| Bulk | 41.6–42.1<br>41.9 ± 0.1 | 24.1–24.5<br>24.3 ± 0.1 | 18.2–18.7<br>18.4 ± 0.1 | 4.6–4.3<br>5.0 ± 0.1 | 5.2–5.5<br>5.4 ± 0.1 | 5.0–5.1<br>5.0 ± 0.0 |
| A15-$Nb_3Sn$ (X=Al,Si,Sn) | 50.9–53.4<br>52.2 ± 0.8 | 25.5–26.0<br>25.8 ± 0.1 | 3.4–5.3<br>4.4 ± 0.5 | 5.9–7.0<br>6.2 ± 0.2 | 2.0–2.9<br>2.4 ± 0.3 | 8.6–9.5<br>9.0 ± 0.2 |
| $Nb_5Si_3$ | 38.0–38.9<br>38.7 ± 0.3 | 17.4–18.7<br>17.9 ± 0.4 | 32.8–34.3<br>33.6 ± 0.5 | 2.3–3.5<br>2.8 ± 0.4 | 5.3–6.1<br>5.7 ± 0.2 | 1.1–1.5<br>1.3 ± 0.1 |
| Ti & Hf rich $Nb_5Si_3$ | 26.4–27.7<br>27.1 ± 0.4 | 24.4–25.0<br>24.8 ± 0.2 | 34.1–35.3<br>34.6 ± 0.3 | 3.0–3.8<br>3.4 ± 0.2 | 9.5–9.8<br>9.7 ± 0.1 | 0.0–0.8<br>0.4 ± 0.2 |

**Table S3:** EPMA data (at%) of the as-cast and heat-treated alloy EZ6.

| **Condition, area in the ingot and phase** | **Nb** | **Ti** | **Si** | **Cr** | **Hf** | **Sn** |
|---|---|---|---|---|---|---|
| **As cast *** | | | | | | |
| Top | 41.3–42.0<br>41.5 ± 0.3 | 22.7–24.5<br>23.5 ± 0.5 | 18.9–21.8<br>20.6 ± 0.5 | 3.8–5.0<br>4.4 ± 0.3 | 5.1–5.3<br>5.2 ± 0.0 | 4.5–5.0<br>4.8 ± 0.1 |
| Bulk | 40.8–42.3<br>41.4 ± 0.4 | 22.9–25.4<br>24.1 ± 0.5 | 17.7–21.4<br>19.6 ± 0.6 | 3.9–5.5<br>4.7 ± 0.3 | 5.0–5.5<br>5.2 ± 0.1 | 4.8–5.4<br>5.0 ± 0.2 |
| Bottom | 37.0–41.5<br>40.3 ± 0.4 | 22.5–29.3<br>24.9 ± 0.7 | 14.8–21.7<br>18.9 ± 0.9 | 4.3–7.6<br>5.3 ± 0.5 | 5.2–6.3<br>5.5 ± 0.3 | 4.9–5.2<br>5.1 ± 0.1 |
| $Nb_{ss}$ | 46.9–56.1<br>50.5 ± 0.5 | 26.8–31.9<br>29.8 ± 0.6 | 1.5–2.4<br>1.9 ± 0.2 | 4.9–8.9<br>7.2 ± 0.4 | 3.4–4.6<br>3.9 ± 0.2 | 6.3–7.3<br>6.7 ± 0.2 |
| Ti-rich $Nb_{ss}$ | 35.2–47.4<br>42.0 ± 0.8 | 31.7–42.4<br>35.0 ± 0.9 | 1.2–2.5<br>1.7 ± 0.3 | 7.8–12.0<br>10.0 ± 0.3 | 3.5–5.3<br>4.2 ± 0.3 | 6.0–8.5<br>7.1 ± 0.4 |
| A15-$Nb_3X$ (X=Si,Sn) | 50.4–55.4<br>53.4 ± 0.2 | 22.0–25.2<br>23.0 ± 0.6 | 5.1–5.8<br>5.4 ± 0.2 | 1.9–3.4<br>2.6 ± 0.4 | 2.1–2.6<br>2.4 ± 0.1 | 12.9–13.7<br>13.2 ± 0.2 |
| $Nb_5Si_3$ | 40.8–42.8<br>41.7 ± 0.3 | 14.1–17.3<br>14.8 ± 0.4 | 33.9–36.7<br>36.0 ± 0.5 | 0.0–2.0<br>0.9 ± 0.2 | 3.8–5.0<br>4.8 ± 0.2 | 1.4–2.2<br>1.8 ± 0.1 |
| Ti & Hf-rich $Nb_5Si_3$ | 22.7–38.5<br>29.2 ± 1.1 | 16.2–26.6<br>22.7 ± 0.8 | 31.4–38.6<br>37.0 ± 0.6 | 0.0–2.4<br>1.0 ± 0.5 | 6.2–10.5<br>8.9 ± 0.6 | 0.0–2.6<br>1.2 ± 0.6 |
| Cr-rich C14 Laves phase | 15.8–22.6<br>19.1 ± 1.2 | 14.9–26.8<br>18.3 ± 0.7 | 6.8–13.0<br>9.8 ± 0.7 | 34.4–48.7<br>44.8 ± 0.8 | 5.6–8.2<br>7.0 ± 0.6 | 0.0–3.4<br>1.0 ± 0.8 |
| **Heat-treated - 1500 °C/100 h (EZ6-HT1)** | | | | | | |
| Bulk | 41.3–42.7<br>42.2 ± 0.3 | 22.1–24.2<br>23.2 ± 0.4 | 19.2–21.3<br>20.3 ± 0.3 | 3.5–4.8<br>4.2 ± 0.2 | 4.9–5.4<br>5.1 ± 0.1 | 4.4–5.6<br>5.0 ± 0.2 |
| $Nb_{ss}$ | 49.7–50.5<br>50.1 ± 0.2 | 31.3–33.6<br>32.4 ± 0.4 | 0.8–1.0<br>0.9 ± 0.1 | 5.8–8.5<br>7.2 ± 0.3 | 2.8–3.2<br>3.0 ± 0.1 | 6.2–6.6<br>6.4 ± 0.1 |
| A15-$Nb_3X$ (X=Si,Sn) | 47.5–47.9<br>47.7 ± 0.1 | 26.5–27.7<br>26.9 ± 0.2 | 3.8–4.6<br>4.3 ± 0.2 | 4.4–4.7<br>4.6 ± 0.1 | 2.3–2.4<br>2.3 ± 0.0 | 14.0–14.3<br>14.2 ± 0.1 |
| $Nb_5Si_3$ | 38.9–42.5<br>40.7 ± 0.4 | 14.1–17.1<br>15.6 ± 0.2 | 36.4–36.6<br>36.5 ± 0.1 | 0.3 | 4.9–5.7<br>5.3 ± 0.1 | 1.6–1.6<br>1.6 ± 0.0 |
| Ti & Hf-rich $Nb_5Si_3$ | 25.4–25.7<br>25.5 ± 0.1 | 24.1–24.3<br>24.2 ± 0.1 | 38.2–38.3<br>38.2 ± 0.1 | 1.0–1.0<br>1.0 ± 0.0 | 10.4–10.5<br>10.5 ± 0.0 | 0.4–0.7<br>0.6 ± 0.1 |
| Cr-rich C14 Laves phase | 27.1–30.1<br>28.1 ± 0.4 | 14.2–14.7<br>14.4 ± 0.1 | 5.4–5.7<br>5.6 ± 0.1 | 43.4–44.4<br>44.7 ± 0.1 | 4.2–4.5<br>4.4 ± 0.1 | 2.0–3.4<br>2.8 ± 0.3 |
| **Heat-treated - 1200 °C/100 h (EZ6-HT2)** | | | | | | |
| Bulk | 40.2–42.4<br>41.7 ± 0.5 | 23.3–24.5<br>23.8 ± 0.3 | 17.9–21.0<br>19.3 ± 0.7 | 3.7–5.7<br>4.7 ± 0.4 | 5.1–5.6<br>5.4 ± 0.3 | 4.7–5.7<br>5.1 ± 0.4 |
| $Nb_{ss}$ | 53.5–57.2<br>55.2 ± 0.5 | 28.9–31.6<br>30.7 ± 0.6 | 0.0–0.9<br>0.6 ± 0.2 | 5.9–8.2<br>7.2 ± 0.5 | 2.2–2.5<br>2.3 ± 0.1 | 3.5–4.6<br>4.0 ± 0.3 |
| A15-$Nb_3X$ (X=Si,Sn) | 44.7–47.1<br>45.7 ± 0.5 | 30.2–31.7<br>30.9 ± 0.5 | 2.4–4.1<br>3.0 ± 0.4 | 2.7–3.7<br>3.2 ± 0.3 | 1.8–2.5<br>2.0 ± 0.2 | 14.2–15.9<br>15.2 ± 0.3 |
| $Nb_5Si_3$ | 41.9–44.9<br>42.5 ± 0.6 | 13.6–17.1<br>14.5 ± 0.5 | 30.2–36.9<br>35.5 ± 0.6 | 0.0–1.0<br>0.6 ± 0.2 | 4.7–5.2<br>5.0 ± 0.1 | 1.5–2.3<br>1.9 ± 0.2 |
| Ti & Hf-rich $Nb_5Si_3$ | 18.2–26.0<br>21.8 ± 0.5 | 25.3–27.8<br>26.4 ± 0.4 | 31.5–40.0<br>37.3 ± 0.7 | 0.5–1.4<br>0.8 ± 0.3 | 10.4–14.7<br>12.7 ± 0.8 | 0.0–3.4<br>1.0 ± 0.7 |
| Cr-rich C14 Laves phase | 21.4–23.8<br>21.9 ± 0.4 | 11.4–13.5<br>12.2 ± 0.4 | 8.0–13.3<br>9.6 ± 0.6 | 44.4–52.9<br>49.7 ± 0.6 | 5.2–6.9<br>5.8 ± 0.3 | 0.0–1.2<br>0.8 ± 0.2 |

* For the $Nb_{ss}$+$NbCr_2$ eutectic see text

**Table S4:** EPMA data (at%) of the as-cast and heat-treated alloy EZ8.

| **Condition, area in the ingot and phase** | **Nb** | **Ti** | **Si** | **Al** | **Cr** | **Hf** | **Sn** |
|---|---|---|---|---|---|---|---|
| **As cast** | | | | | | | |
| Top | 36.9–37.5<br>37.1 ± 0.1 | 23.5–24.3<br>23.8 ± 0.2 | 19.0–21.0<br>20.0 ± 0.3 | 4.4–4.7<br>4.6 ± 0.1 | 4.2–4.8<br>4.4 ± 0.2 | 5.1–5.4<br>5.2 ± 0.1 | 4.9–5.0<br>4.9 ± 0.0 |
| Bulk | 36.9–38.0<br>37.2 ± 0.3 | 23.6–24.3<br>24.1 ± 0.2 | 17.9–19.7<br>18.8 ± 0.4 | 4.6–5.0<br>4.8 ± 0.1 | 4.3–5.6<br>4.7 ± 0.2 | 5.1–5.3<br>5.2 ± 0.0 | 4.7–5.3<br>5.2 ± 0.1 |
| Bottom | 35.5–37.0<br>36.3 ± 0.3 | 24.3–26.4<br>25.9 ± 0.4 | 13.3–17.9<br>14.7 ± 0.7 | 5.0–5.5<br>5.4 ± 0.1 | 5.6–6.5<br>6.1 ± 0.2 | 5.1–6.2<br>5.7 ± 0.2 | 5.1–6.1<br>5.9 ± 0.2 |
| $Nb_{ss}$ | 30.7–37.2<br>35.9 ± 0.5 | 34.2–37.9<br>36.4 ± 0.4 | 0.9–6.0<br>1.7 ± 0.8 | 5.9–7.4<br>6.7 ± 0.4 | 9.2–14.4<br>10.9 ± 0.7 | 2.9–5.0<br>3.7 ± 0.6 | 3.8–5.6<br>4.7 ± 0.4 |
| A15-$Nb_3X$<br>X=Al,Si,Sn | 42.1–51.9<br>48.0 ± 0.9 | 22.1–27.1<br>25.0 ± 0.5 | 3.6–7.7<br>4.3 ± 0.7 | 4.7–6.8<br>5.5 ± 0.5 | 2.1–5.5<br>3.6 ± 0.6 | 2.0–3.4<br>2.5 ± 0.4 | 8.8–12.3<br>11.1 ± 0.8 |
| $Nb_5Si_3$ | 35.7–40.5<br>38.9 ± 0.9 | 17.1–20.5<br>18.2 ± 0.5 | 28.4–33.7<br>30.4 ± 0.5 | 2.0–4.5<br>3.7 ± 0.6 | 0.4–2.3<br>1.2 ± 0.2 | 4.0–5.3<br>4.5 ± 0.3 | 1.7–4.2<br>3.1 ± 0.5 |
| Ti & Hf-rich $Nb_5Si_3$ | 23.2–29.3<br>25.8 ± 06 | 22.0–26.2<br>24.6 ± 0.4 | 23.4–34.6<br>32.0 ± 0.8 | 3.5–5.2<br>4.1 ± 0.4 | 0.7–4.6<br>1.8 ± 0.6 | 9.4–11.7<br>10.3 ± 0.5 | 0.5–2.9<br>1.4 ± 0.6 |
| $NbCr_2$ - $Nb_{ss}$ Eutectic | 20.4–26.7<br>23.2 ± 0.5 | 14.3–26.5<br>21.7 ± 0.9 | 4.8–10.3<br>7.0 ± 0.6 | 3.1–5.2<br>4.4 ± 0.3 | 29.2–45.5<br>35.8 ± 1.1 | 4.9–6.7<br>6.0 ± 0.4 | 0.5–2.8<br>1.9 ± 0.4 |
| $NbCr_2$ Laves | 19.0–19.8<br>19.4 ± 0.2 | 14.0–15.1<br>14.6 ± 0.2 | 9.1–10.8<br>10.3 ± 0.2 | 3.1–3.6<br>3.4 ± 0.1 | 44.3–47.0<br>45.6 ± 0.5 | 6.0–6.7<br>6.4 ± 0.1 | 0.3 |
| **Heat-treated - 1300 °C/100 h** | | | | | | | |
| Bulk | 37.7–39.2<br>38.1 ± 0.2 | 22.6–24.2<br>23.6 ± 0.4 | 17.7–20.3<br>19.1 ± 0.3 | 4.3–4.8<br>4.5 ± 0.1 | 3.8–6.8<br>4.7 ± 0.4 | 4.9–5.4<br>5.3 ± 0.1 | 4.3–5.0<br>4.7 ± 0.1 |
| A15-$Nb_3X$<br>X=Al,Si,Sn | 46.8–47.9<br>47.4 ± 0.2 | 26.3–27.1<br>26.7 ± 0.2 | 2.9–4.1<br>3.2 ± 0.2 | 6.3–6.7<br>6.5 ± 0.1 | 4.3–4.8<br>4.6 ± 0.1 | 1.4–1.9<br>1.6 ± 0.1 | 9.6–10.2<br>10.0 ± 0.1 |
| $Nb_5Si_3$ | 40.0–41.6<br>41.1 ± 0.2 | 17.0–18.6<br>17.5 ± 0.3 | 31.1–33.1<br>32.3 ± 0.2 | 2.2–2.9<br>2.5 ± 0.1 | 0.4–1.0<br>0.6 ± 0.2 | 3.9–4.6<br>4.1 ± 0.1 | 1.7–2.1<br>1.9 ± 0.1 |
| Ti&Hf-rich $Nb_5Si_3$ | 22.8–25.8<br>24.4 ± 0.4 | 24.7–26.2<br>25.6 ± 0.3 | 31.5–34.1<br>33.3 ± 0.3 | 3.7–5.0<br>4.0 ± 0.2 | 0.9–2.1<br>1.3 ± 0.3 | 9.7–12.6<br>10.6 ± 0.4 | 0.5–1.2<br>0.8 ± 0.2 |
| $NbCr_2$ Laves phase | 22.3–22.6<br>22.4 ± 0.1 | 10.4–11.0<br>10.7 ± 0.1 | 9.1–9.6<br>9.3 ± 0.1 | 2.6–2.9<br>2.7 ± 0.1 | 49.5–50.3<br>50.0 ± 0.2 | 4.8–5.0<br>4.9 ± 0.0 | – |

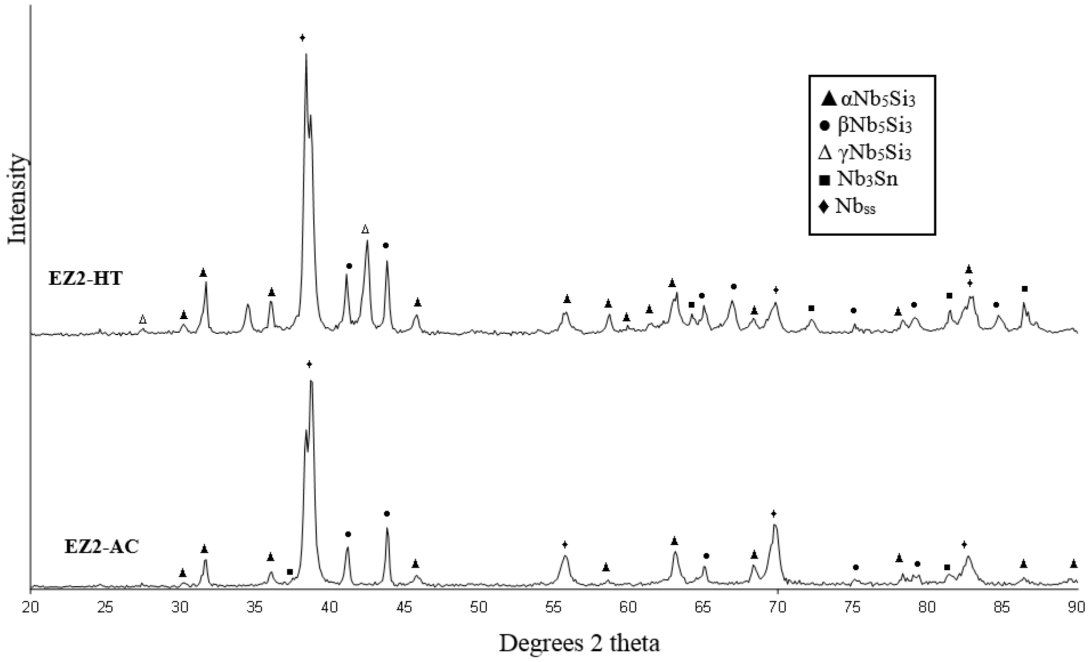


**Figure S1:** X-ray diffractograms of the as-cast and heat-treated alloy EZ2.

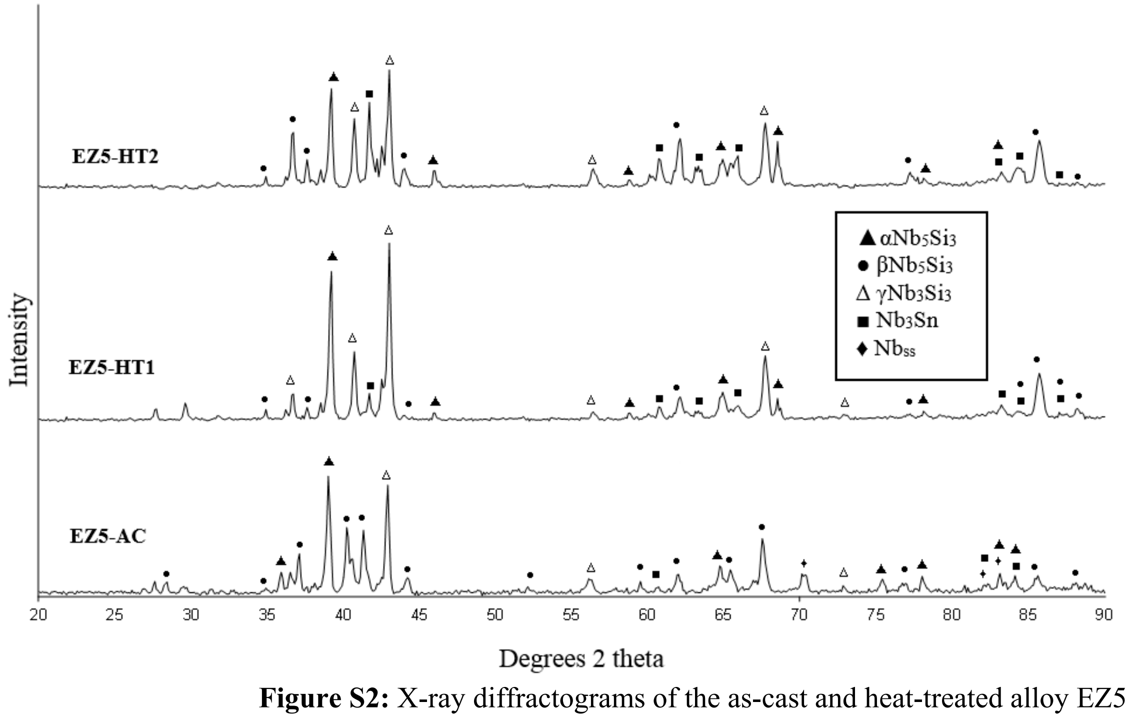


**Figure S2:** X-ray diffractograms of the as-cast and heat-treated alloy EZ5.

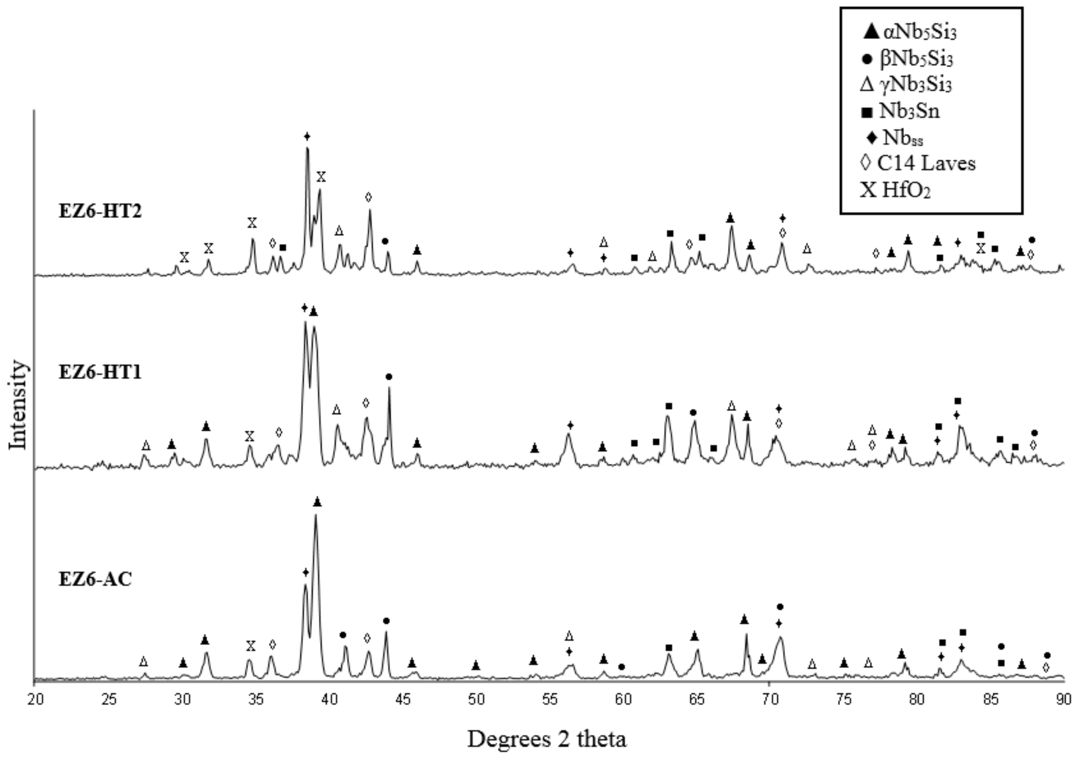


**Figure S3:** X-ray diffractograms of the as-cast and heat-treated alloy EZ6.

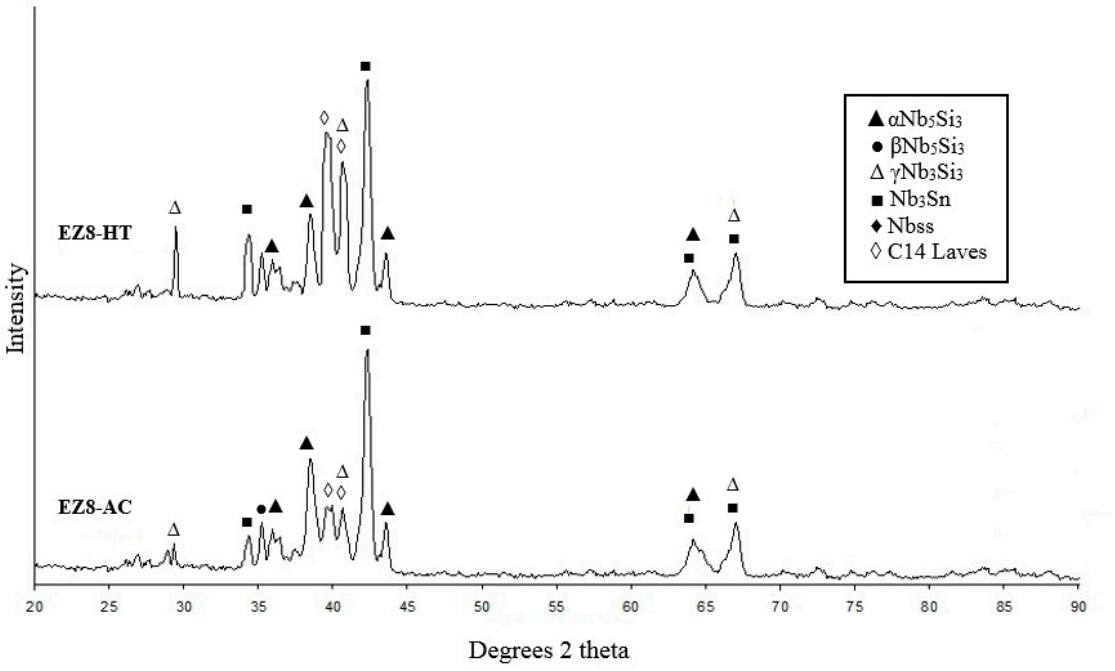


**Figure S4:** X-ray diffractograms of the as-cast and heat-treated alloy EZ8.
